# Tuning the Molecular Packing of Self‐Assembled Amphiphilic Pt^II^ Complexes by Varying the Hydrophilic Side‐Chain Length

**DOI:** 10.1002/chem.202003445

**Published:** 2021-02-03

**Authors:** Lorena Herkert, Philipp Selter, Constantin G. Daniliuc, Nils Bäumer, Jasnamol P. Palakkal, Gustavo Fernández, Michael Ryan Hansen

**Affiliations:** ^1^ Organisch-Chemisches Institut Westfälische Wilhelms-Universität Münster Corrensstraße, 40 48149 Münster Germany; ^2^ Institut für Physikalische Chemie Westfälische Wilhelms-Universität Münster Corrensstraße 28/30 48149 Münster Germany; ^3^ Department of Materials and Earth Sciences Technische Universität Darmstadt Alarich-Weiss-Straße 2 64287 Darmstadt Germany

**Keywords:** amphiphilic molecules, Pt^II^ complexes, Self-assembly, weak non-covalent interactions, pi-conjugated systems

## Abstract

Understanding the relationship between molecular design and packing modes constitutes one of the major challenges in self‐assembly and is essential for the preparation of functional materials. Herein, we have achieved high precision control over the supramolecular packing of amphiphilic Pt^II^ complexes by systematic variation of the hydrophilic side‐chain length. A novel approach of general applicability based on complementary X‐ray diffraction and solid‐state NMR spectroscopy has allowed us to establish a clear correlation between molecular features and supramolecular ordering. Systematically increasing the side‐chain length gradually increases the steric demand and reduces the extent of aromatic interactions, thereby inducing a gradual shift in the molecular packing from parallel to a long‐slipped organization. Notably, our findings highlight the necessity of advanced solid‐state NMR techniques to gain structural information for supramolecular systems where single‐crystal growth is not possible. Our work further demonstrates a new molecular design strategy to modulate aromatic interaction strengths and packing arrangements that could be useful for the engineering of functional materials based on Pt^II^ and aromatic molecules.

## Graphical abstract

**X‐ray diffraction** and advanced solid‐state NMR studies were combined as a new, general tool for detailed structural and supramolecular elucidation, using a series of amphiphilic Pt^II^ complexes as model compounds. Our approach provides an alternative for supramolecular systems where single‐crystal growth is not possible, which can be potentially applied to many other molecular and macromolecular platforms.

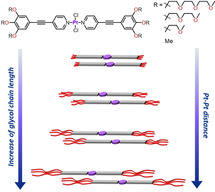

## Introduction

Self‐assembled structures of π‐conjugated systems have long been an area of active research in materials science, where molecular assemblies in solution, in the solid state and in the liquid crystalline state can be created.^[1]^ It is widely known that the functional properties of self‐assembled structures are highly dependent on the molecular structure of the monomers, and that minor changes in the molecular design can result in dramatic differences in the features and functionalities of the resulting materials.^[2]^ Therefore, tuning the balance of attractive noncovalent interactions (such as hydrogen bonding and aromatic interactions) and steric effects by molecular design is necessary to generate self‐assembled materials with distinct properties.^[2]^ Outside purely organic compounds, the introduction of a metal ion like Pt^II^ to π‐conjugated molecules can further extend the range of possible intermolecular interactions and enable additional applications in optoelectronics and biomedicine.^[3]^ Hence, control of the resulting structures through molecular design is desirable.

As previously shown, tuning the length of peripheral alkyl chains—which are commonly used to achieve solubility of the compounds—can dramatically affect the self‐assembly behavior of Pt^II^ complexes. For instance, we reported the self‐assembly of two oligophenyleneethynylene (OPE)‐based bispyridyldichlorido Pt^II^ complexes featuring terminal dodecyloxy versus methoxy chains.^[4]^ We found that shortening the side chains causes a drastic change from a slipped arrangement of the molecules, both in solution and the solid state, to almost parallel π‐stacks in the solid state with enhanced aromatic interactions and shortened Pt−Pt distances of ≈4.4 Å. In another elegant example, Yam and co‐workers showed that the hydrophobic tail length of amphiphilic Pt^II^ complexes can alter the morphologies and emission behavior of self‐assemblies in aqueous medium.^[5]^ Additionally, for different luminescent Pt^II^ mesogens, it has been demonstrated that the variation of alkyl chains can be used to modulate the mesophases with regards to their stability and photophysical behavior.^[6]^ Interestingly, modification of the alkyl tail length for some of these compounds has led to the emergence of multistimuli‐responsive polymorphism in the solid state,[^6a^, ^6b^] thus demonstrating the key impact of small structural changes (e.g., C_14_ vs. C_16_ chain^[6a]^) on the final properties of the systems.

Apart from nonpolar alkyl chains, polar oligo‐ or poly(ethylene glycol) chains (OEG, PEG) are commonly used as hydrophilic counterparts for achieving solubility in aqueous media.^[7]^ However, the effect of EG chain length variation on the self‐assembly of Pt^II^ complexes has been scarcely investigated.^[8]^ To the best of our knowledge, the only example of self‐assembling Pt^II^ complexes featuring PEG_*n*_ chains of different length was reported by the group of Manners.^[9]^ In this work, tetrazole‐based tridentate Pt^II^ complexes with ancillary ligands based on PEG_*n*_ were observed to form 1D fibers (*n*=16) or 2D platelets (*n*=7) in polar solvents, depending on the PEG chain length.

**Scheme 1:**
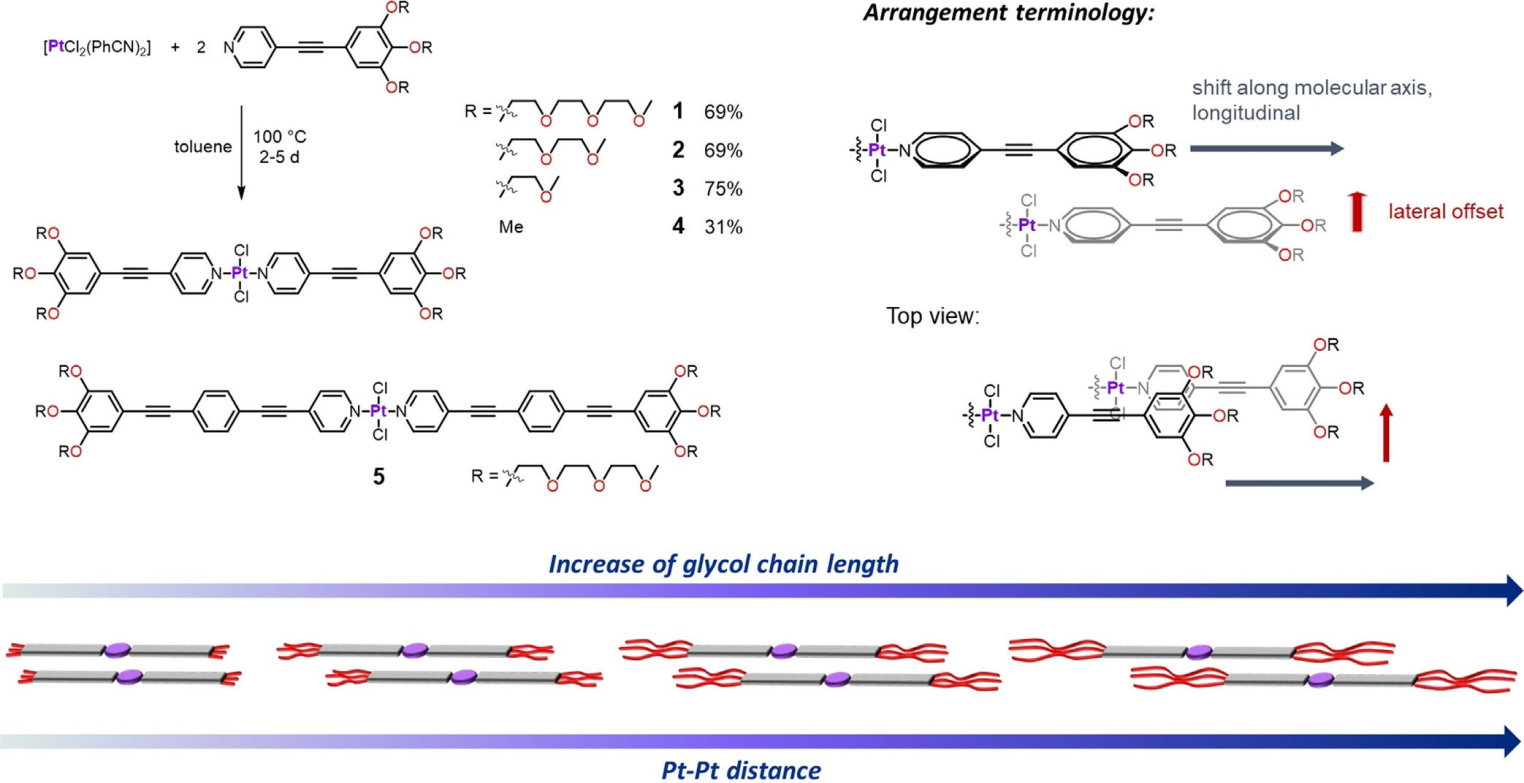
Top left: Synthesis of the Pt^II^ complexes **1** *–* **4** and molecular structures of **1** *–* **5**. Top right: Representation of longitudinal and lateral shift of two stacked molecules. Bottom: Schematic depiction of the side‐chain length–dependent packing modes of **1** *–* **4** in the solid state.

**Figure 1:**
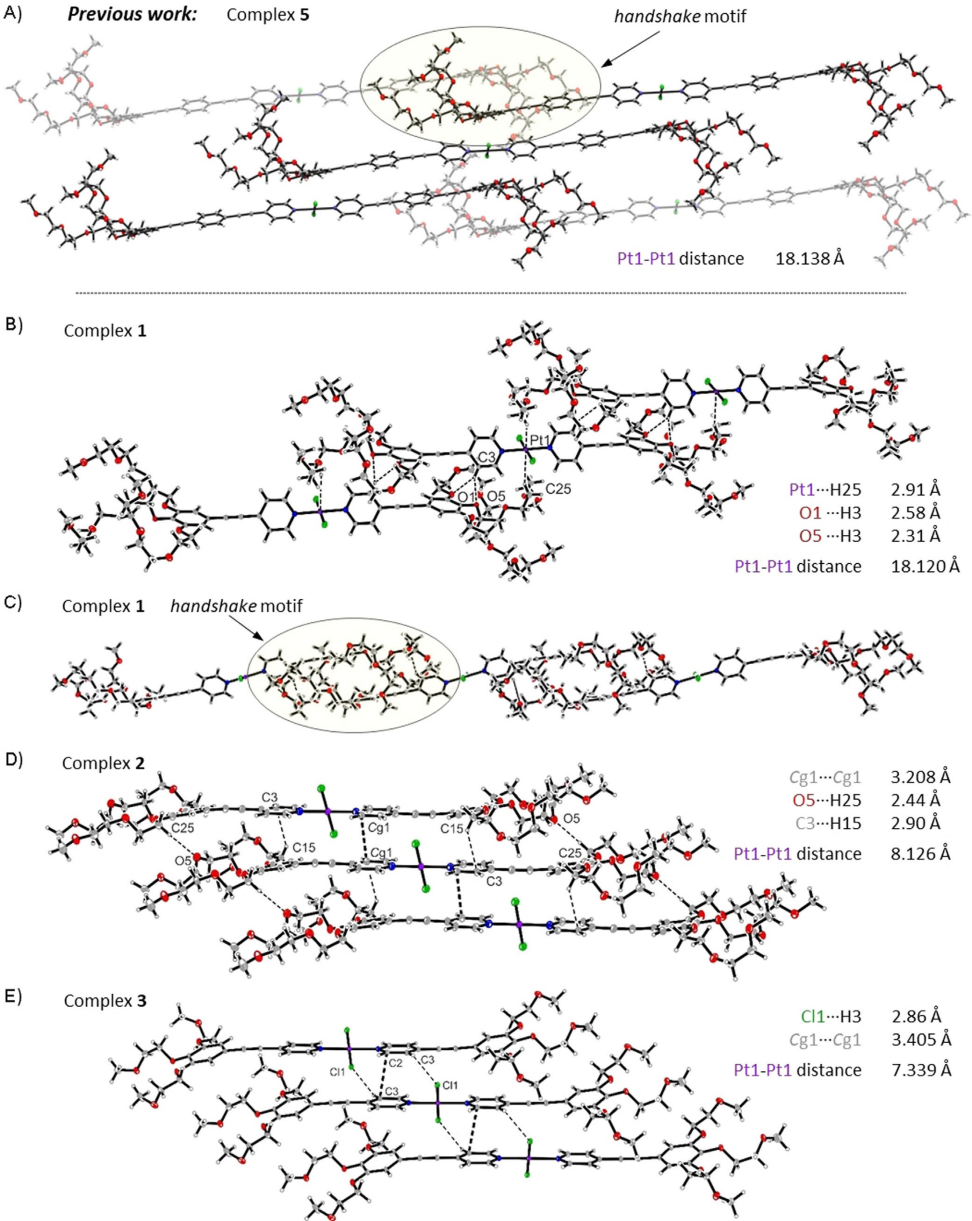
Excerpts of the packing diagrams from the crystal structures of A) **5**, B), C) **1**, D) **2**, and E) **3** showing the stacking of molecules and change from the handshake motif (C) between adjacent amphiphilic molecules (**1** and **5**) to the more parallel stacking of molecules (**2** and **3**) caused by the gradual shortening of the attached hydrophilic side chains from TEG (**1**), DEG (**2**) to EG (**3**).

In this work, we demonstrate precise control over the supramolecular packing of amphiphilic Pt^II^ complexes by systematic variation of the hydrophilic side‐chain length. This understanding could contribute to establishing a correlation between molecular design and packing modes, which remains one of the greatest challenges in the field of self‐assembly.^[10]^ To this end, we herein investigated the solid‐state structures of a series of rod‐like bispyridyldichlorido Pt^II^ complexes **1**–**4** featuring either three OEG chains on both termini, namely triethylene glycol (TEG, **1**), diethylene glycol (DEG, **2**), ethylene glycol (EG, **3**), or methoxy groups (**4**, Scheme 1). Complexes **1**–**4** are smaller structural analogues of a previously reported Pt^II^ complex **5** that was found to exhibit a unique molecular organization based on its crystal structure: the presence of voluminous TEG chains hinders a parallel molecular arrangement and ultimately enables the formation of a characteristic *handshake* motif, where the TEG groups of two adjacent Pt^II^ units are in close contact through multiple CH⋅⋅⋅O interactions (Scheme 1 and Figure 1 A).^[11]^ To simplify our system, we shortened the aromatic backbone of the ligand to two rings (pyridine‐ethynylphenylene) in order to reduce the amount of potential interactions, highlighting the influence of the glycol chains with identical π‐surface. We envisioned that a gradual decrease of the EG chain length while keeping the aromatic surface of the molecules unchanged could progressively alleviate the steric demand of the chains and, consequently, reinforce the extent of π‐overlap between the stacked molecules. This, in turn, is expected to lead to a gradual transformation from a more slipped to more parallel packing upon shortening the EG side chains. Thus, in this report, we focus on the self‐assembly structures in the solid state, whereas investigations on the processes in solution are still ongoing.

Usually, the solid‐state structure and molecular organization are determined by single‐crystal X‐ray diffraction, and we were able to obtain suitable single crystals for complexes **1** to **3**. Crystallization of complex **4**, however, resulted in polycrystalline powders unsuitable for X‐ray analysis. Therefore, a complementary method was required for obtaining a systematic comparison of all compounds. To this end, we employed solid‐state ^1^H and ^13^C magic‐angle spinning (MAS) NMR in combination with 2D, “through‐space” NMR correlation techniques based on the direct magnetic dipole–dipole (or dipolar) interaction between nuclear spins to elucidate the aggregation of the methoxy‐functionalized complex **4**.^[12]^ Our approach consisted in identifying through‐space ^1^H,^13^C correlations characteristic for certain structural motifs by comparing the ^1^H and ^13^C MAS NMR results obtained from complexes **1** to **3** with the crystallographic data. On this basis, we are able to readily identify or exclude previously observed packing structures for this specific series of OPE‐based Pt^II^ complexes, filling in the missing structural information from crystallographic data.

## Results and Discussion

### Synthesis

Pt^II^ complexes **1**–**4** were synthesized in moderate to good yields by complexation reaction of the precursor salt [PtCl_2_(PhCN)_2_] with the respective pyridine‐based ligands in toluene at 100 °C (Scheme 1 and the Supporting Information). Complexes **1**–**3** resulted in crystalline solids upon concentration from Et_2_O or pentane‐containing CH_2_Cl_2_ as well as EtOAc solutions, whereas all other used solvents led to highly amorphous structures with soft texture. Compound **4**, however, could only be obtained as a powdery solid, which is poorly soluble in most organic solvents due to the short side chains. All complexes could be characterized by ^1^H/^13^C NMR in solution, solid‐state NMR, and gave good results for elemental analyses.

### Crystal structure analysis

By slow vapor diffusion of Et_2_O into solutions of **1**–**3** in EtOAc, pale yellow single crystals suitable for X‐ray diffraction analysis could be grown. **1** and **2** both were solved and refined in the triclinic space group *P*‐1, whereas **3** could be solved in the monoclinic space group *P*2_1_/*n* (for details of crystal structure determinations see Figures S1–S6 and Tables S1–S3). In the following, the arrangements of the stacked molecules in the solid state will be characterized in terms of a longitudinal shift along the molecular axis as well as by a lateral offset as depicted in Scheme 1. Figure 1 shows the obtained molecular structures and packing of complexes **1** to **3** in their crystal structures. For comparison, an excerpt of the crystal structure of previously reported analogue **5** is also shown. Relevant close intermolecular contacts are highlighted. In the crystal structure of **1** (Figure 1 B), the packing can be viewed as an arrangement of longitudinally shifted molecular units mainly stabilized by C−H_arom_⋅⋅⋅O (2.31 Å, 2.58 Å) interactions and a close Pt⋅⋅⋅H contact (2.91 Å) between the central Pt atom of one molecule and the proton of the inner glycol chain of a neighboring molecule. Such Pt⋅⋅⋅H interactions have been observed in earlier reports and can be explained by donation of electron density from the d_z_^2^ orbital of the Pt to the σ* orbital of the C−H.^[13]^ Interestingly, no aromatic interactions are observed in the crystal structure of **1** (for a top view of the π‐system illustrating the orientation of the aromatic rings, see Figure S2). Instead, the structure formation is dominated by the TEG chains, which form a large, bulky structure surrounding the neighboring aromatic backbone in a so‐called *handshake* motif (Figure 1 C), similarly to that observed for complex **5** (Figure 1 A).^[11]^ The lack of close π–π contacts for **1** compared to the higher homologue **5** can be explained by the considerably larger aromatic surface for the latter (six *vs*. four aromatic rings), which enables a more efficient π‐overlap of the OPE backbone in the crystal. The relatively short π‐system of **1** must orient in a twisted manner with a torsion angle of the two aromatic rings (phenyl and pyridine) of 53.8° to form the present structure. Stabilization of the *handshake* motif is achieved by intermolecular C−H_arom_⋅⋅⋅O interactions exhibiting distances in the range of previously observed weak hydrogen bonding for such systems (2.41–2.56 Å).^[11]^ The closest Pt−Pt distance that can be found in the packing of **1** is 9.854 Å, whereas the Pt−Pt distance illustrating the longitudinal shift of two molecules is 18.120 Å (Figure 1 B).

In the single crystal structure of **2**, a distinct change in the arrangement compared to **1** is apparent (Figure 1 D). Although the molecules still arrange in a slipped manner, the change from TEG to DEG side chains facilitates a molecular stacking through π–π interactions, leading to an assembly with a decreased longitudinal molecular shift (inner stack Pt−Pt distance 8.126 Å vs. 18.120 Å in **1**) and a slight lateral offset (see also Figure S4). The parallel displaced π‐stacking of two pyridine units has a distance of ≈3.2 Å. In contrast to the structure of **1**, where the Pt^II^Cl_2_ fragment is surrounded by TEG chains, the Pt^II^Cl_2_ moiety in **2** is located on top of the C≡C bond of the next molecule in the stack. The structure is further stabilized by C−H_glycol_⋅⋅⋅O (2.44 Å) as well as C−H_glycol_⋅⋅⋅C_arom_ (2.90 Å) interactions. Thus, instead of a *handshake* motif as in **1**, the DEG side chains in **2** interdigitate with neighboring side chains (Figure S4). Furthermore, an intermolecular weak C−H_glycol_⋅⋅⋅Cl (2.84 Å) interaction is observed within adjacent stacks (Figure S4).

A shortening of the glycol chains to one unit (EG) in **3** results in a similar molecular stacking to complex **2** (Figure 1 E) with a Pt−Pt distance that is even slightly shorter for **3** (7.339 Å) compared to **2** (8.126 Å). Parallel displaced π–π interactions of the pyridine rings with a distance of ≈3.4 Å and C−H_arom_⋅⋅⋅Cl (2.86 Å) interactions stabilize the molecular packing. Additional C−H_glycol_⋅⋅⋅O (2.43 Å), C−H_glycol_⋅⋅⋅C_arom_ (2.41 Å) and C−H_glycol_⋅⋅⋅C≡C (2.87 Å) interactions of one stack with molecules of obliquely arranged neighboring stacks are also present (Figure S6).

In contrast to compounds **1**–**3**, single crystals of **4** could not be obtained. However, considering the trend followed by **1**–**3**, we would expect a more parallel arrangement of the molecules in **4** (shorter Pt−Pt distances) and enhanced aromatic interactions compared to **1**–**3** due to the lack of any side chains. Due to no available single crystal X‐ray diffraction data for complex **4**, a complementary technique (solid‐state NMR) was required to elucidate the molecular arrangement in the solid state.

### Insights into the molecular packing from solid‐state NMR

Our approach consisted in identifying *inter*molecular contacts characteristic for the packing motifs observed in **1**–**3** and **5**. By comparing these contacts with those potentially observed (or alternatively *not* observed) for **4**, a packing arrangement for this complex can be derived. All samples used for solid‐state NMR measurements were powdered samples and were obtained under identical conditions as those used for the preparation of the single crystals discussed in the previous section (EtOAc/Et_2_O solutions). We therefore assumed identical (or at the very least comparable) arrangements of the molecules in the samples for solid‐state NMR studies and in the crystalline state. Additionally, we have conducted powder XRD analysis of **1**–**4** to further probe this assumption. A detailed summary and analysis of the XRD results can be found in the Supporting Information. Compound **1** shows the lowest crystallinity of all samples obtained by the precipitation protocol (Figure S18). While the positions of the reflexes are in relatively good agreement with the predicted XRD pattern (based on the corresponding single‐crystal data), the overall intensity is greatly diminished in particular for the range corresponding to the long‐range order. The sterically demanding TEG chains, which adopt a highly ordered orientation in the single crystal, most likely prevent this long‐range order in the powder sample; nevertheless, a similar short‐range order could be confirmed. On the other hand, the higher crystallinity of **2** is reflected in the sharp reflexes, which match the predicted XRD pattern well (Figure S19). Yet, the observation of some minor discrepancies, in particular for the immediate environment of the Pt^II^ moiety, indicates that the high level of interdigitation of the DEG chains might not be perfectly transferred to the powdered sample used for solid‐state NMR studies. Nevertheless, based on the reasonably good agreement between the experimental and predicted XRD patterns, we infer that the orientation within the 1D stack is largely preserved. For the sterically least demanding compound for which single‐crystal analysis was possible (**3**), the predicted XRD pattern reproduces the experimental results without discrepancies (Figure S20). This result is logical considering the absence of significant steric effects when the chains are reduced to EG units. On this basis, we infer that the molecular orientation in the bulk as well as in the single crystal matches. For compound **4**, sharp reflexes could be appreciated as well, which suggests that the sample has a highly crystalline nature, which matches the observed trend for compounds **1**–**3** (Figure S21).

**Figure 2:**
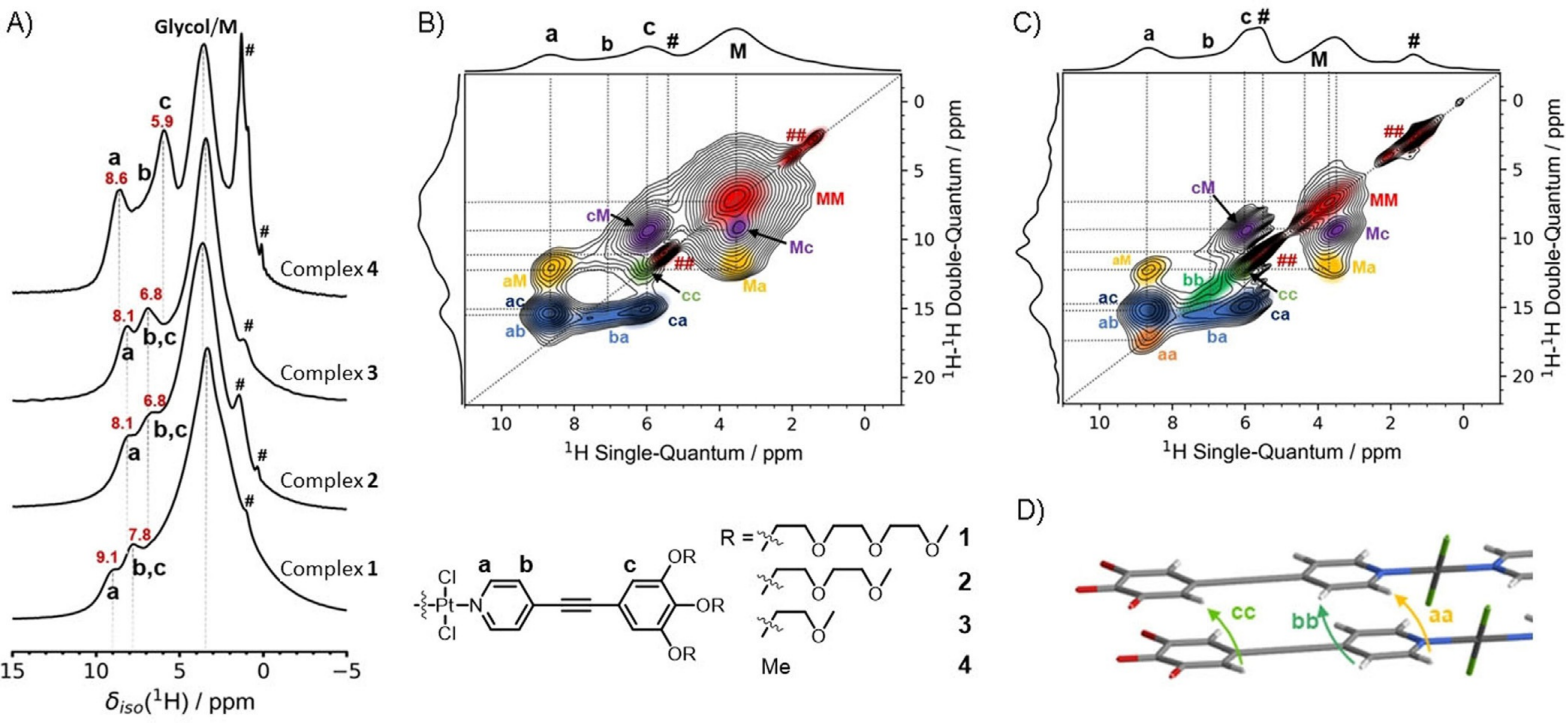
A) ^1^H MAS NMR spectra of complexes **1** to **4**, acquired at 11.7 T (500 MHz) using 29.762 kHz MAS. The ^1^H chemical shifts of the discernable aromatic protons (**a**–**c**) are highlighted in red; the dashed lines are guides to the eye. # indicates residual solvent and “Glycol/M” denotes the position of the oligoglycol and methoxy protons. ^1^H,^1^H DQ‐SQ correlation spectrum of complex **4** at 29.762 kHz MAS using B) one and C) four rotor periods of DQ recoupling. D) The proposed packing arrangement for **4**.

Figure 2 A summarizes the ^1^H MAS NMR spectra of **1**–**4**. Overall, broad ^1^H lines with few discernable details are observed due to the strong ^1^H,^1^H dipolar couplings present in the solid state. Nevertheless, apart from the broad peak around *δ*_iso_(^1^H)=3.5 ppm assigned to the protons of the EG moieties, two signals in the aromatic region are observed: while the signal at higher ppm values (*δ*_iso_(^1^H)=9.1 to 8.1 ppm) can be assigned to the aromatic protons adjacent to the pyridine nitrogen (H_α_, position **Ha** in Figure 2), the ^1^H signal at lower values (*δ*_iso_(^1^H)=7.8–6.8 ppm) corresponds to the aromatic protons **Hb** (H_β_‐pyridyl) and **Hc**. The exact position of the two peaks varies between complexes **1** to **4**, going from *δ*_iso_(^1^H_a_)=9.1 ppm and *δ*_iso_(^1^H_b,c_)=7.8 ppm in complex **1**, to *δ*_iso_(^1^H_a_)=8.1 ppm and *δ*_iso_(^1^H_b,c_)=6.8 ppm in complexes **2** and **3**. As the moieties are structurally identical, the difference can be solely attributed to packing effects, in this case to aromatic ring current effects caused by the neighboring aromatic backbone in **2** and **3**, or the lack thereof in **1**. The shift to lower ppm values is often indicative of π–π interactions as observed for example in π‐conjugated polymers and other conjugated systems.^[14]^

Replacing the EG moieties with methoxy groups as in complex **4** leads to a markedly different ^1^H MAS NMR spectrum, cf. Figure 2 A. The signal at *δ*_iso_(^1^H)=3.5 ppm is now significantly reduced in intensity and width, and strong shifts of the ^1^H signals in the aromatic region are observed. The upfield shift of proton **c** suggests an even stronger participation of the outer ring in π–π interactions compared to complexes **2** and **3**. In contrast, the ^1^H signal **a** is situated at a higher ppm value than that of **2** and **3**, which might lead to the wrong conclusion that the pyridyl ring of **4** undergoes weaker aromatic interactions than that of **2** and **3**. However, this effect is typical for bispyridyldichlorido Pt^II^ and Pd^II^ complexes exhibiting a pseudo‐parallel packing, and can be attributed to C−H_a_⋅⋅⋅Cl interactions between the α‐pyridyl protons of one molecule and the electron‐withdrawing Cl ligands of an adjacent molecule.^[4]^

To further probe the molecular packing of complex **4**, 2D ^1^H,^1^H double‐quantum single‐quantum correlation (DQ‐SQ) MAS NMR experiments were performed (Figure 2 B, C and S9). This kind of experiment allows to identify ^1^H,^1^H spin pairs in close spatial proximity to each other (up to ≈3.5 Å in rigid organic solids and longer distances if the systems are flexible or proton diluted).^[15]^ The 2D spectra of complexes **1** to **3** (Figures S8 and S9) are characterized by a broad auto‐correlation signal situated at *δ*_SQ_=3.5 ppm and *δ*_DQ_=7.0 ppm (attributed to the EG units) with some smaller auto‐correlation peaks visible at lower ppm values most likely originating from the residual trapped solvent. However, no autocorrelation signals in the aromatic region (around *δ*_SQ_=7 ppm) are observed.

In analogy to the ^1^H MAS NMR spectrum, replacing the EG units with methoxy groups leads to the appearance of three aromatic ^1^H signals in the DQ‐SQ correlation spectra (Figure 2 B, C). While the cross‐correlation signals of protons **Ha** and **Hc** are readily discernible at *δ*_SQ_=8.6 ppm and *δ*_SQ_=5.9 ppm (*δ*_DQ_=14.6 ppm, dark blue, marked **ac** and **ca**), the broad Gaussian peak associated with protons **Hb** at around *δ*_SQ_=7 ppm is mostly obscured. In addition to the cross‐correlation between the methoxy protons and aromatic proton **Hc** at *δ*_DQ_=9.4 ppm—which is expected due to the close spatial proximity of the methoxy groups on the same ring—a cross‐correlation between the methoxy protons and proton **Ha** is observed at *δ*_DQ_=12.2 ppm. Interestingly, no cross‐correlation between the aromatic proton **Hb** and the methoxy protons is observable, though this could be related to the broad nature and comparably low amplitude of the peak at *δ*_SQ_=7.0 ppm. These results suggest that the methoxy groups are placed in relatively close proximity to the complex center, though whether this is from above/below the aromatic backbone or from other neighboring complex is unclear at this point. The most significant difference between the spectra of **4** and the other three samples is the appearance of several auto‐correlation peaks in the aromatic chemical shift range (marked orange **aa**, blue‐green **bb**, and yellow‐green **cc**). While these are barely discernible after one rotor period of DQ excitation (Figure 2 B), their amplitude increases when going to longer (four rotor periods) DQ excitation times (Figure 2 C). This indicates a ^1^H–^1^H distance of around 3.5–4.0 Å, pointing to π–π stacking of the monomers with a parallel arrangement of the molecular units (Figure 2 D).

To summarize the results from ^1^H MAS and ^1^H,^1^H DQ‐SQ NMR correlation data, ^1^H MAS NMR revealed changes in chemical shift of the aromatic protons associated with differences in aromatic ring current effects, with **1** showing weaker and **2** and **3** showing stronger ring current effects. Protons **Ha** and **Hb** in **4** show a moderate effect of ≈0.5 ppm, which would indicate a packing motif where the protons are further away from the neighboring aromatic ring center pointing towards the edges of the said ring, though proximity of **Ha** to the electron‐withdrawing Cl (H_a_⋅⋅⋅Cl) could lead to the shift to higher ppm. These observations fit well with a packing model similar to the pseudo‐parallel molecular arrangement observed by Allampally et al. for a longer, methoxy‐functionalized OPE‐based Pt^II^ complex.^[4]^

**Figure 3:**
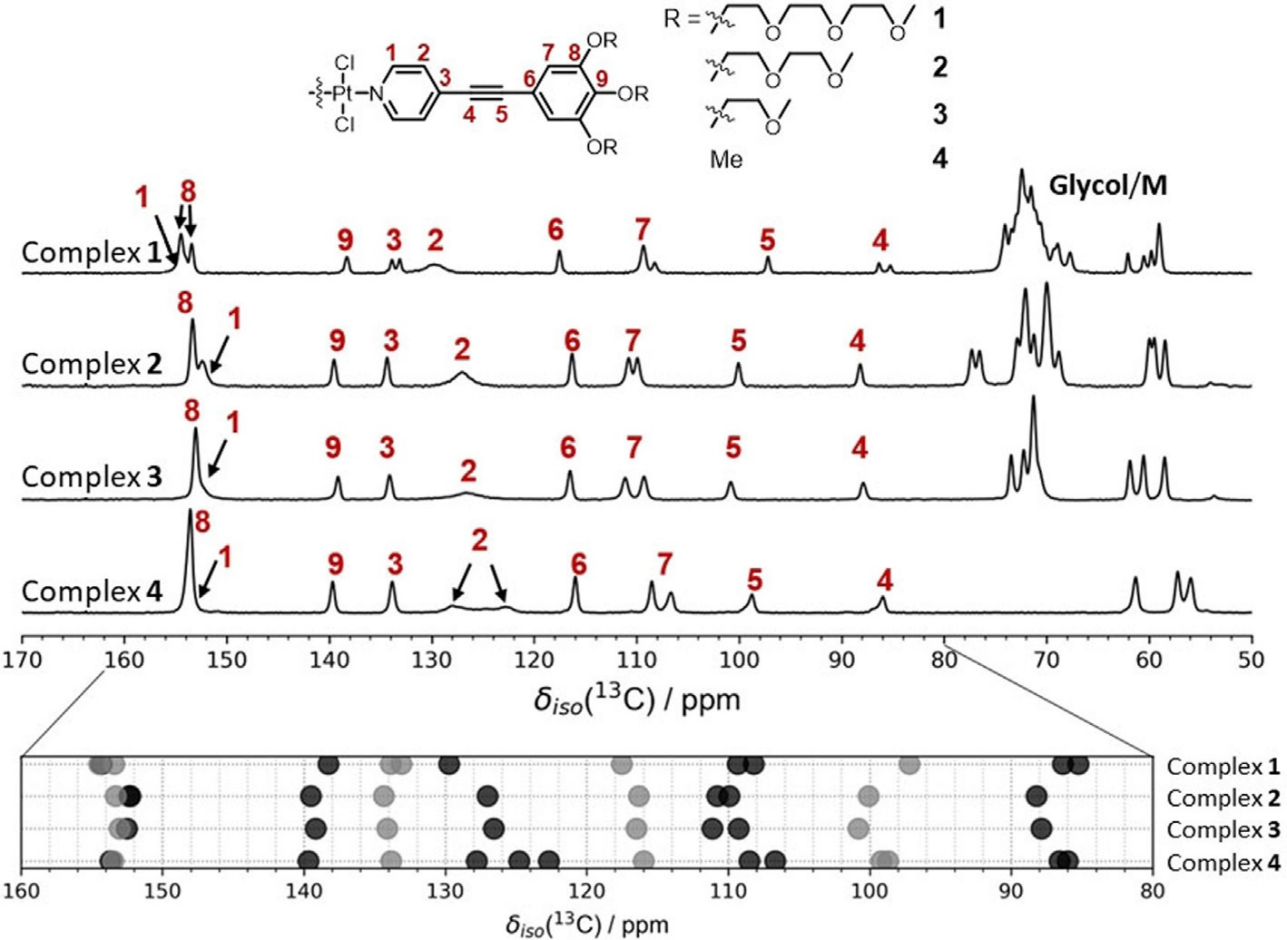
^13^C{^1^H} CP/MAS spectra of **1** to **4** acquired using a CP contact time of 2.0 ms at 9.4 T (**2**–**4**) and 7.05 T (**1**). The ^13^C assignment scheme (numbers) of the OPE backbone is shown at the top. Note that signal 1 is severely broadened under these CP conditions and overlaps with position 8. The plot at the bottom shows the variation of the ^13^C chemical shift between the complexes **1** to **4** highlighting changes in ^13^C chemical shift.

To confirm the results from ^1^H MAS and ^1^H,^1^H DQ‐SQ NMR correlation spectroscopy, ^13^C{^1^H} cross‐polarization (CP/MAS) and 2D ^13^C{^1^H} heteronuclear correlation (HETCOR) NMR experiments were performed (details are given in Figures S12–S17). Figure 3 summarizes the ^13^C{^1^H} CP/MAS NMR spectra of complexes **1**–**4** along with the peak assignment. Note that the assignment uses the numbering given in Figure 3, which is different from the numbering of the carbons in the crystal structures. The assignment is based on a combination of variable contact time CP measurements for complex **1** as well as the corresponding 2D ^13^C{^1^H} HETCOR NMR data (Figure S14). To better visualize the changes in isotropic ^13^C chemical shift for the different complexes, the ^13^C signals associated with the OPE backbone are plotted in the lower part of Figure 3. The most striking difference between the spectrum of complex **4** and those of complexes **1**–**3** is the splitting of the broad ^13^C signal at *δ*_iso_(^13^C)=129 −122 ppm, assigned to the *β*‐carbon of the pyridine moiety (C_β_; position **C2**). This carbon is directly bonded to proton **Hb**, which was previously attributed to a broad Gaussian‐shaped peak in the ^1^H MAS NMR spectra (vide supra). Thus, the doublet character of this ^13^C signal indicates a more complex ^1^H line shape, with the Gaussian distribution being merely an approximation, which further explains the complex cross‐correlation peaks observed in the ^1^H,^1^H DQ‐SQ NMR spectrum of Figure 2 B, C. As the substitution of the EG groups occurs on the outer phenyl ring, these changes observed for **C2** and **Hb** must be related to intermolecular packing effects. Another interesting feature of Figure 3 is the low‐field shift of the alkyne carbons **C4** and **C5** for complexes **2** and **3** compared to complex **1**. This low‐field shift is not observed for complex **4**. Because the packing mode switches from the *handshake* motif with a long‐shifted arrangement in complex **1** to a less shifted, *staircase* pattern in complexes **2** and **3** with the Pt^II^Cl_2_ center being close to the alkyne triple bond, we can attribute the low‐field shift to a change in packing.

**Figure 4:**
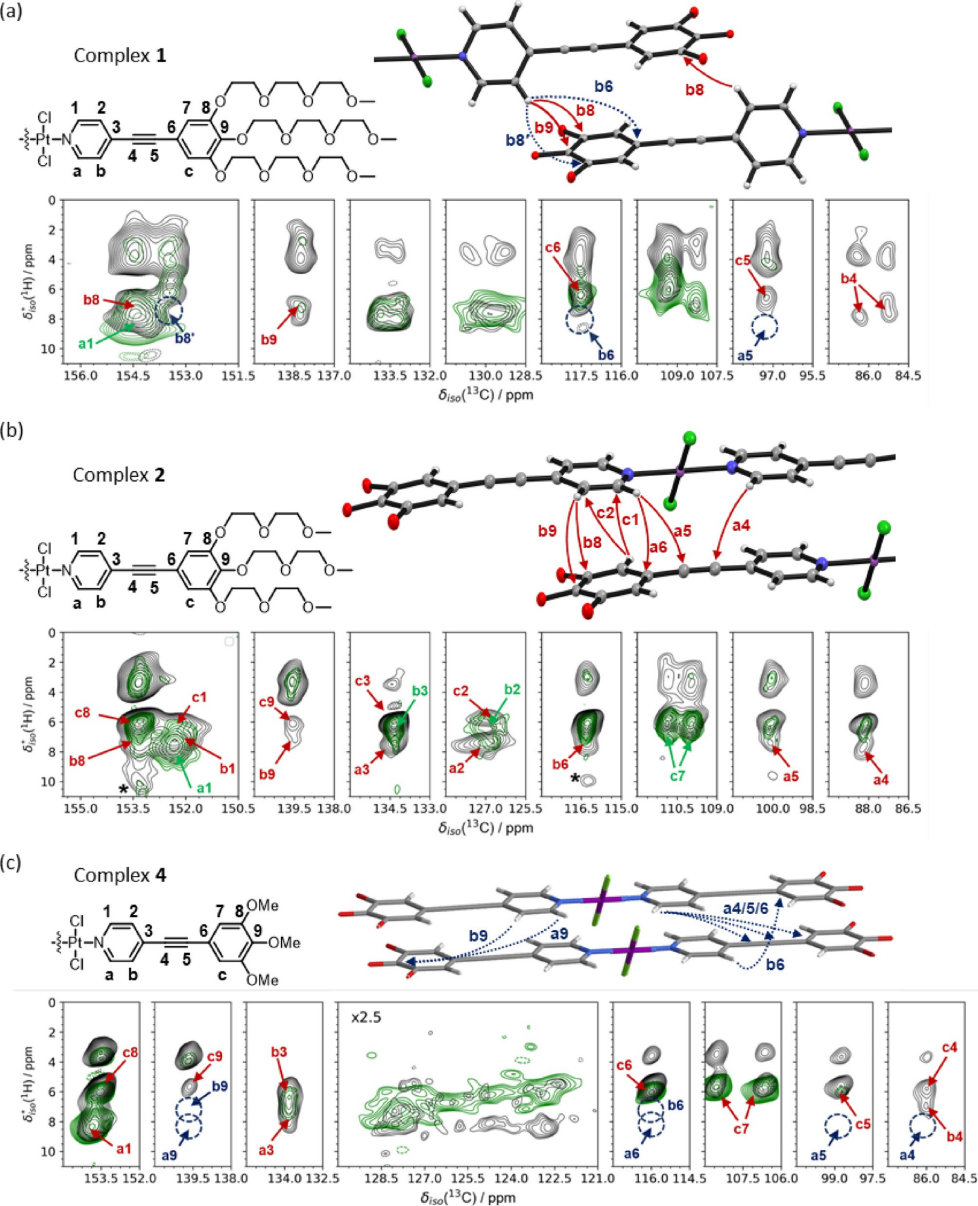
2D ^13^C{^1^H} HETCOR spectra and crystal structure fragments for (a) **1** and (b) **2** depicting the relative orientation of two successively stacked molecules. (c) Proposed molecular packing and 2D ^13^C{^1^H} HETCOR spectra for **4**. Side chains have been omitted for clarity. All 2D HETCOR spectra include two different CP contact times of 3.0 ms (black) and 0.5 ms (green) contour lines to differentiate between intermolecular ^1^H‐^13^C contacts and directly bonded ^1^H‐^13^C correlations, respectively. The assignment uses numbers for ^1^H and letters for ^13^C positions as indicated in each molecular fragment. Red/green arrows and assignments to specific ^1^H‐^13^C contacts indicate observed correlations; dark blue arrows and circles indicate correlations that are not observed as discussed in the main text. Asterisks indicate spinning side bands.

Several of the ^13^C signals across all four complexes in Figure 3 are split into doublets, most notably the ^13^C signals assigned to carbon **C7** at *δ*_iso_(^13^C)=∼109 ppm, as well as those for **C3**, **C4**, and **C8** in the case of complex **1**. The splitting of carbons **C7** and **C8** can be explained by two inequivalent carbon sites in the asymmetric unit of the crystal structure. However, according to the single‐crystal XRD data, carbons **C3** and **C4** only occur once in the asymmetric unit of complex **1**. Therefore, it is likely that the sample used for MAS NMR has a slightly different packing and additional disorder compared to the single crystal used for XRD (cf. Figures S18–S20). Figure 4 summarizes the 2D ^13^C{^1^H} HETCOR spectra for **1**, **2**, and **4** acquired with 0.5 ms (green contour lines) and 3.0 ms CP contact time (black contour lines) to differentiate between directly bonded ^1^H–^13^C and *inter*molecular ^1^H–^13^C contacts, respectively. A comparison of these 2D HETCOR NMR datasets (green and black) allows us to identify the packing structure for each complex as illustrated by the structural fragments and corresponding assignments of ^1^H,^13^C correlations below these spectra (see caption of Figure 4 for details of the assignment). The spectrum of complex **1** shows two ^13^C signals for carbon **C8** due to the existence of two inequivalent positions in the crystallographic asymmetric unit. As for complex **5** (data shown in Figures S12 and 13), this also results in different *inter*molecular ^1^H–^13^C distances for the two positions, manifested in the 2D HETCOR NMR spectra as correlations **HbC8** and **HbC8′** labeled as **b8** and **b8′**, respectively, in Figure 4 A (complex **1**). The large longitudinal shift between two molecules in **1** is also evident from the correlation **HbC9**, which links proton **Hb** (located at the complex center) to the outermost OPE carbon **C9**, a correlation that is only possible via *inter*molecular contacts. A further indicator for the magnitude of the longitudinal shift is the absence of potential *inter*molecular correlations **HbC6** and **HaC5**, which would be expected for an intermediate shift (these are present in the case of complexes **2** and **3**, see Figure 4 B).

Since the solid‐state 2D HETCOR NMR spectra of **2** and **3** are very similar, only those for **2** will be discussed in the following (Figure 4 B). As mentioned before, the crystal structures of **2** and **3** lack the characteristic TEG *handshake* motif and feature a moderately shifted aromatic π–π stacking arrangement. This packing can be confirmed from the 2D HETCOR datasets in Figure 4 B for **2** (see Figure S16 for **3**), which shows numerous *inter*molecular correlations, most notably **HbC6**, **HaC5**, **HaC4**, **HbC9**, **HcC2**, and **HcC1**. Although **HbC9** and **HbC8** were also observed for complex **1**, correlations **HcC1**, and **HcC2** place the pyridine moiety close to the second phenyl ring of the OPE backbone. Interestingly, the correlations between **C7** (bonded to proton **Hc**) and protons **Ha** or **Hb** (attached to carbons **C1** and **C2**) are not observed. In addition, correlations **HaC5** and **HaC4**—which were not observed for complex **1**—are indicators for the Pt^II^Cl_2_ complex center being in the proximity to the alkyne triple bond of the neighboring molecule.

The 2D HETCOR spectra of **4** together with the proposed molecular arrangement are shown in Figure 4 C. In contrast to the 2D HETCOR spectra for **1** and **2**, none of the *inter*molecular correlations such as **HbC9**, **HaC9**, **HaC6**, **HaC5**, and **HaC4** are observed. This can only be achieved for a structural arrangement with a parallel or nearly parallel π–π stacked arrangement of the OPE backbones, similar to the one described by Allampally et al. for the larger OPE‐based complex with methoxy functionalization.^[4]^ Such a structural arrangement is also supported by the ^1^H,^1^H DQ‐SQ NMR correlation data (Figure 2). Thus, through a rigorous comparison and careful assignment of observed and unobserved ^1^H,^13^C correlations for complexes **1**–**3** and **5** with known crystal structures, it can be concluded that the aromatic interactions for the OPE backbone of complex **4** occur in a nearly parallel fashion as illustrated by the molecular fragments in Figure 4 C.

## Conclusions

In summary, we have reported the synthesis and molecular packing of four new amphiphilic Pt^II^‐based complexes **1**–**4** with short aromatic OPE backbones and hydrophilic oligo(ethylene glycol) termini of different length. A systematic length variation was achieved by gradually removing one (**2**), two (**3**) or three (**4**) OCH_2_‐CH_2_ units from the TEG‐substituted derivative **1**. Data from single‐crystal X‐ray diffraction showed that complex **1** retained the characteristic *handshake* motif previously described for complex **5**, which features the same TEG side chains, but has a larger aromatic surface than **1**. The strong similarity between the packing motifs of **1** and **5** indicate that the TEG groups with their associated hydrophilic interactions are the primary forces dictating the solid‐state structure. However, in contrast to **5**, complex **1** shows no significant aromatic interactions due to the reduced aromatic surface. Shortening the hydrophilic side chains in complexes **2** and **3** resulted in structures with longitudinally shifted molecules involving more pronounced π–π interactions, though the bulky Pt^II^Cl_2_ moiety in the complex center prevents efficient π–π stacking between neighboring complexes. Two‐dimensional solid‐state ^1^H MAS and ^13^C MAS NMR experiments for complexes **1**, **2**, **3**, and **5** allowed us to identify ^1^H,^13^C through‐space correlations characteristic for each packing motif. These, in turn, led us to propose a packing motif for the only complex (**4**) for which single crystals could not be grown, emphasizing the potential of solid‐state NMR spectroscopy as a key tool for structural determination in the solid state. Complex **4** was found to exhibit a more parallel arrangement of the molecules stabilized by π–π interactions, most likely with a short longitudinal and lateral offset to accommodate the sterically demanding Pt^II^Cl_2_ moiety at the complex center. On this basis, we conclude that the steric demand of the central Pt^II^Cl_2_ moiety limits the effectiveness of π–π interactions; this is supported by the fact that even short mono(ethylene glycol) termini appear to prevent an efficient π‐overlap of the ligands aromatic surface. A more pronounced π‐overlap is only possible after eliminating hydrophilic interactions by replacing the ethylene glycol termini with methoxy groups. Therefore, if π–π interactions are frustrated by steric demands, hydrophilic interactions will become the dominant factor in the self‐assembly process, leading to structural aggregation. Thus, our investigations illustrate that even minor changes in the glycol chain length can be used to modulate and control aromatic interaction strengths and packing arrangements, which in turn might be useful for engineering electronic devices based on Pt^II^ and aromatic molecules.

## Experimental Section

See the Supporting Information for details on single‐crystal diffraction and solid‐state NMR experiments. Deposition numbers 2004413 (**1**), 2004414 (**2**), and 2004415 (**3**) contain the supplementary crystallographic data for this paper. These data are provided free of charge by the joint Cambridge Crystallographic Data Centre and Fachinformationszentrum Karlsruhe Access Structures service.

## Conflict of interest

The authors declare no conflict of interests.

## Supporting information

As a service to our authors and readers, this journal provides supporting information supplied by the authors. Such materials are peer reviewed and may be re‐organized for online delivery, but are not copy‐edited or typeset. Technical support issues arising from supporting information (other than missing files) should be addressed to the authors.

Supplementary

## References

[1a] K. Ariga , M. Nishikawa , T. Mori , J. Takeya , L. K. Shrestha , J. P. Hill , Sci. Technol. Adv. Mater. 2019, 20, 51–95; 30787960 10.1080/14686996.2018.1553108 PMC6374972

[1b] T. Kato , J. Uchida , T. Ichikawa , T. Sakamoto , Angew. Chem. Int. Ed. 2018, 57, 4355–4371; 10.1002/anie.201711163 29534321

[1c] M. V. Ivanov , D. Wang , T. S. Navale , S. V. Lindeman , R. Rathore , Angew. Chem. Int. Ed. 2018, 57, 2144–2149; 10.1002/anie.201712159 29327390

[1d] D. B. Amabilino , D. K. Smith , J. W. Steed , Chem. Soc. Rev. 2017, 46, 2404–2420; 28443937 10.1039/c7cs00163k

[1e] M. Gsänger , D. Bialas , L. Huang , M. Stolte , F. Würthner , Adv. Mater. 2016, 28, 3615–3645; 27028553 10.1002/adma.201505440

[1f] S. Rosenne , E. Grinvald , E. Shirman , L. Neeman , S. Dutta , O. Bar-Elli , R. Ben-Zvi , E. Oksenberg , P. Milko , V. Kalchenko , H. Weissman , D. Oron , B. Rybtchinski , Nano Lett. 2015, 15, 7232–7237; 26447786 10.1021/acs.nanolett.5b02010

[1g] C. Shahar , J. Baram , Y. Tidhar , H. Weissman , S. R. Cohen , I. Pinkas , B. Rybtchinski , ACS Nano 2013, 7, 3547–3556. 23521176 10.1021/nn400484y

[2a] C. Kulkarni , M. H. C. van Son , D. Di Nuzzo , S. C. J. Meskers , A. R. A. Palmans , E. W. Meijer , Chem. Mater. 2019, 31, 6633–6641;

[2b] C. Kulkarni , J. A. Berrocal , M. Lutz , A. R. A. Palmans , E. W. Meijer , J. Am. Chem. Soc. 2019, 141, 6302–6309; 30920829 10.1021/jacs.9b00452

[2c] A. Liess , A. Lv , A. Arjona-Esteban , D. Bialas , A.-M. Krause , V. Stepanenko , M. Stolte , F. Würthner , Nano Lett. 2017, 17, 1719–1726; 28165244 10.1021/acs.nanolett.6b04995

[2d] S. Y.-L. Leung , K. M.-C. Wong , V. W.-W. Yam , Proc. Natl. Acad. Sci. USA 2016, 113, 2845–2850; 26933213 10.1073/pnas.1601673113 PMC4801284

[2e] F. Aparicio , S. Cherumukkil , A. Ajayaghosh , L. Sánchez , Langmuir 2016, 32, 284–289; 26645962 10.1021/acs.langmuir.5b03771

[2f] A. Arjona-Esteban , J. Krumrain , A. Liess , M. Stolte , L. Huang , D. Schmidt , V. Stepanenko , M. Gsänger , D. Hertel , K. Meerholz , F. Würthner , J. Am. Chem. Soc. 2015, 137, 13524–13534. 26414767 10.1021/jacs.5b06722

[3a] A. Aliprandi , D. Genovese , M. Mauro , L. de Cola , Chem. Lett. 2015, 44, 1152–1169;

[3b] V. W.-W. Yam , V. K.-M. Au , S. Y.-L. Leung , Chem. Rev. 2015, 115, 7589–7728; 26158432 10.1021/acs.chemrev.5b00074

[3c] M. Mauro , A. Aliprandi , D. Septiadi , N. S. Kehr , L. de Cola , Chem. Soc. Rev. 2014, 43, 4144–4166; 24643393 10.1039/c3cs60453e

[3d] J. J. Wilson , S. J. Lippard , Chem. Rev. 2014, 114, 4470–4495. 24283498 10.1021/cr4004314 PMC3999256

[4] N. K. Allampally , M. J. Mayoral , S. Chansai , M. C. Lagunas , C. Hardacre , V. Stepanenko , R. Q. Albuquerque , G. Fernández , Chem. Eur. J. 2016, 22, 7810–7816. 27113990 10.1002/chem.201600176

[5] C. Po , A. Y.-Y. Tam , V. W.-W. Yam , Chem. Sci. 2014, 5, 2688.

[6a] C. Cuerva , J. A. Campo , M. Cano , C. Lodeiro , Chem. Eur. J. 2019, 25, 12046–12051; 31237959 10.1002/chem.201901763

[6b] M. Krikorian , S. Liu , T. M. Swager , J. Am. Chem. Soc. 2014, 136, 2952–2955; 24533433 10.1021/ja4114352

[6c] C.-T. Liao , H.-H. Chen , H.-F. Hsu , A. Poloek , H.-H. Yeh , Y. Chi , K.-W. Wang , C.-H. Lai , G.-H. Lee , C.-W. Shih , P.-T. Chou , Chem. Eur. J. 2011, 17, 546–556; 21207572 10.1002/chem.201000994

[6d] X. Wu , M. Zhu , D. W. Bruce , W. Zhu , Y. Wang , J. Mater. Chem. C 2018, 6, 9848–9860;

[6e] G. Qian , X. Yang , X. Wang , J. D. Herod , D. W. Bruce , S. Wang , W. Zhu , P. Duan , Y. Wang , Adv. Optical Mater. 2020, 8, 2000775;

[6f] X. Yang , X. Wu , D. Zhou , J. Yu , G. Xie , D. W. Bruce , Y. Wang , Dalton Trans. 2018, 47, 13368–13377. 30207369 10.1039/c8dt03017k

[7] E. Krieg , M. M. C. Bastings , P. Besenius , B. Rybtchinski , Chem. Rev. 2016, 116, 2414–2477. 26727633 10.1021/acs.chemrev.5b00369

[8a] V. Saez Talens , D. M. M. Makurat , T. Liu , W. Dai , C. Guibert , W. E. M. Noteborn , I. K. Voets , R. E. Kieltyka , Polym. Chem. 2019, 10, 3146–3153;

[8b] I. Gadwal , S. De , M. C. Stuparu , S. G. Jang , R. J. Amir , A. Khan , J. Polym. Sci. Part A 2012, 50, 2415–2420.

[9] M. E. Robinson , A. Nazemi , D. J. Lunn , D. W. Hayward , C. E. Boott , M.-S. Hsiao , R. L. Harniman , S. A. Davis , G. R. Whittell , R. M. Richardson , L. de Cola , I. Manners , ACS Nano 2017, 11, 9162–9175. 28836765 10.1021/acsnano.7b04069

[10a] I. Helmers , B. Shen , K. K. Kartha , R. Q. Albuquerque , M. Lee , G. Fernández , Angew. Chem. Int. Ed. 2020, 59, 5675–5682; 10.1002/anie.201911531 PMC7154731 31849157

[10b] N. M. Matsumoto , R. P. M. Lafleur , X. Lou , K.-C. Shih , S. P. W. Wijnands , C. Guibert , J. W. A. M. van Rosendaal , I. K. Voets , A. R. A. Palmans , Y. Lin , E. W. Meijer , J. Am. Chem. Soc. 2018, 140, 13308–13316. 30221520 10.1021/jacs.8b07697 PMC6194755

[11] C. Rest , M. J. Mayoral , K. Fucke , J. Schellheimer , V. Stepanenko , G. Fernández , Angew. Chem. Int. Ed. 2014, 53, 700–705; 10.1002/anie.201307806 24352814

[12a] L. A. Straasø , Q. Saleem , M. R. Hansen in Annual Reports on NMR Spectroscopy, Vol. 88 (Ed.: G. A. Webb ), Academic Press, London, 2016, pp. 307–383;

[12b] I. Schnell , S. P. Brown , H. Y. Low , H. Ishida , H. W. Spiess , J. Am. Chem. Soc. 1998, 120, 11784–11795;

[12c] C. Ochsenfeld , S. P. Brown , I. Schnell , J. Gauss , H. W. Spiess , J. Am. Chem. Soc. 2001, 123, 2597–2606; 11456929 10.1021/ja0021823

[12d] S. P. Brown , I. Schnell , J. D. Brand , K. Müllen , H. W. Spiess , J. Am. Chem. Soc. 1999, 121, 6712–6718.

[13] N. Komiya, T. Hosokawa, J. Adachi, R. Inoue, S. Kawamorita, T. Naota, *Eur. J. Inorg. Chem*. **2018**, 4771–4778.

[14a] A. Bohle , D. Dudenko , N. Koenen , D. Sebastiani , S. Allard , U. Scherf , H. W. Spiess , M. R. Hansen , Macromol. Chem. Phys. 2018, 219, 1700266;

[14b] A. Melnyk , M. J. N. Junk , M. D. McGehee , B. F. Chmelka , M. R. Hansen , D. Andrienko , J. Phys. Chem. Lett. 2017, 8, 4155–4160; 28809493 10.1021/acs.jpclett.7b01443

[14c] M. Wegner , D. Dudenko , D. Sebastiani , A. R. A. Palmans , T. F. A. de Greef , R. Graf , H. W. Spiess , Chem. Sci. 2011, 2, 2040;

[14d] L. Mafra , S. M. Santos , R. Siegel , I. Alves , F. A. A. Paz , D. Dudenko , H. W. Spiess , J. Am. Chem. Soc. 2012, 134, 71–74; 22118503 10.1021/ja208647n

[14e] J. Shu , D. Dudenko , M. Esmaeili , J. H. Park , S. R. Puniredd , J. Y. Chang , D. W. Breiby , W. Pisula , M. R. Hansen , J. Am. Chem. Soc. 2013, 135, 11075–11086; 23829567 10.1021/ja4029186

[14f] S. R. Chaudhari , J. M. Griffin , K. Broch , A. Lesage , V. Lemaur , D. Dudenko , Y. Olivier , H. Sirringhaus , L. Emsley , C. P. Grey , Chem. Sci. 2017, 8, 3126–3136. 28507688 10.1039/c7sc00053g PMC5413886

[15a] I. Schnell , H. W. Spiess , J. Magn. Reson. 2001, 151, 153–227; 11531343 10.1006/jmre.2001.2336

[15b] S. P. Brown , Prog. Nucl. Magn. Reson. Spectrosc. 2007, 50, 199–251;

[15c] M. R. Hansen , R. Graf , S. Sekharan , D. Sebastiani , J. Am. Chem. Soc. 2009, 131, 5251–5256; 19301900 10.1021/ja8095703

[15d] S. P. Brown , Solid State Nucl. Magn. Reson. 2012, 41, 1–27. 22177472 10.1016/j.ssnmr.2011.11.006

