# Supplementary material for: Tuning the Molecular Packing of Self‐Assembled Amphiphilic PtII Complexes by Varying the Hydrophilic Side‐Chain Length
Source: Chemistry. 2021 Feb 3;27(14):4617–26. doi: 10.1002/chem.202003445 (PMC7986126; doi:10.1002/chem.202003445)
Supplement: Supplementary file 1 — Supplementary [file CHEM-27-4617-s001.pdf]

Chemistry–A European Journal

Supporting Information

Tuning the Molecular Packing of Self-Assembled Amphiphilic Pt^{II} Complexes by Varying the Hydrophilic Side-Chain Length

Lorena Herkert+,[a] Philipp Selter+,[b] Constantin G. Daniliuc,[a] Nils Bäumer,[a] Jasnamol P. Palakkal,[c] Gustavo Fernández,*[a] and Michael Ryan Hansen*[b]

Index

A. Synthesis and Characterization 2

Materials and methods

Experimental procedures

B. Crystal Structure Analysis 35

X-Ray diffraction

X-Ray crystal structure analysis of **1**

X-Ray crystal structure analysis of **2**

X-Ray crystal structure analysis of **3**

C. Solid state NMR 42

Methods

Discussion of the chemical shift

Supplementary solid-state NMR data

D. XRD Analysis 52

Methods

XRD diffractogram of **1-4**

Discussion of XRD diffractograms

E. References 56

A. Synthesis and Characterization

Materials and Methods:

General. All solvents were dried according to standard procedures. Reagents were used as purchased. All air-sensitive reactions were carried out under argon atmosphere. Flash chromatography was performed using silica gel (*Merck Silica 60*, particle size 0.04-0.063 nm). Analytical thin layer chromatography (TLC) was performed on *Fluka silica gel 60 F254* coated aluminum foil.

NMR measurements. ^{1}H NMR and ^{13}C NMR spectra in solution were recorded on *Bruker Avance II 300* and *Bruker Avance II 400* (^{1}H: 300 MHz and 400 MHz, respectively) using partially deuterated solvents as internal standards. Coupling constants (J) are denoted in Hz and chemical shifts (δ) in ppm. Multiplicities are denoted as follows: s = singlet, d = doublet, t = triplet, m = multiplet, br = broad.

Mass spectrometry. All mass spectra were recorded on *Bruker Daltonics MicrOTof ESI* and *Thermo Fisher Scientific Orbitrap LTQ XL*.

Elemental Analysis. CHN analysis was carried out with a *vario EL III Element Analyzer* by *elementar Analysensysteme GmbH*.

Experimental procedures:

*Synthesis of Ligands **19**-**21***:

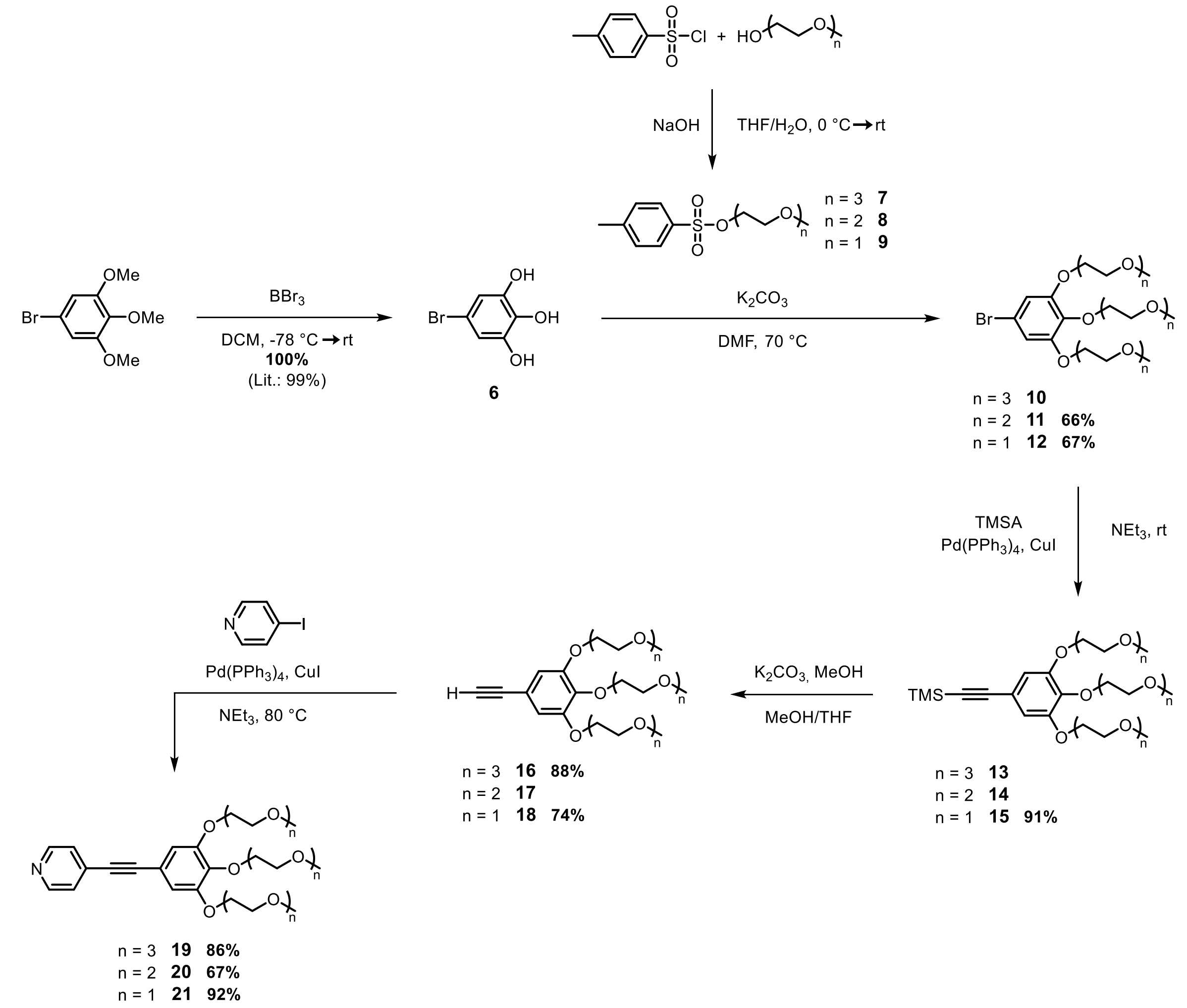

5-Bromo-1,2,3-trihydroxybenzene **6**,[1]
2-(2-(2-methoxyethoxy)ethoxy)ethyl-4-methylbenzenesulfonate **7**,[2]
2-(2-methoxyethoxy)ethyl-4-methylbenzenesulfonate **8**,[2]
2-methoxyethyl-4-methylbenzenesulfonate **9**,[2]
5-bromo-1,2,3-tris(2-(2-(2-methoxyethoxy)ethoxy)ethoxy)benzene **10**,[3]
trimethyl((3,4,5-tris(2-(2-(2-methoxyethoxy)ethoxy)ethoxy)phenyl)ethynyl)silane **13**,[4]
trimethyl((3,4,5-tris(2-(2-methoxyethoxy)ethoxy)phenyl)ethynyl)silane **14**,[5]
5-ethynyl-1,2,3-tris(2-(2-methoxyethoxy)ethoxy)benzene **17**[5] were prepared following reported synthetic procedures and showed identical spectroscopic properties to those reported therein.

*Synthesis of 5-bromo-1,2,3-tris(2-(2-methoxyethoxy)ethoxy)benzene (**11**):* **6** (700 mg, 3.41 mmol) and **8** (3.28, 12.0 mmol) were dissolved in dry DMF (20 mL). The mixture was subjected to five vacuum/argon cycles. K_2CO_3 (2.83 g, 20.5 mmol) was added and the mixture was stirred at 90 °C for 2 days. After cooling down to room temperature, water (40 mL) was added and the solution was extracted with DCM (3 × 80 mL). The combined organic layers were then washed with water (3 x 40 mL) and dried over Na_2SO_4. The product was purified by column chromatography (silica, neat DCM to DCM/MeOH 98:2). Product **11** was obtained as colorless oil (1.16 g, 2.27 mmol, 66%).

MS-ESI: m/z calculated for $[C_{21}H_{35}O_9BrNa]^+$ $[M+Na^+]$: 533.1357; found 533.1370.

^{1}H NMR (300 MHz, $CDCl_3$, 298 K): δ (in ppm) 6.73 (s, 2H, phenyl-*H*), 4.16 – 4.10 (m, 6H, OCH_2), 3.86 – 3.82 (m, 4H, OCH_2), 3.82 – 3.76 (m, 2H, OCH_2), 3.73 – 3.68 (m, 6H, OCH_2), 3.57 – 3.52 (m, 6H, OCH_2), 3.38 (s, 6H, OCH_3), 3.38 (s, 3H, OCH_3).
^{13}C NMR (75 MHz, $CDCl_3$, 298 K): δ (in ppm) 153.4, 137.8, 115.9, 111.4, 72.5, 72.2, 72.1, 70.9, 70.6, 70.6, 69.7, 69.2, 59.2, 59.2.

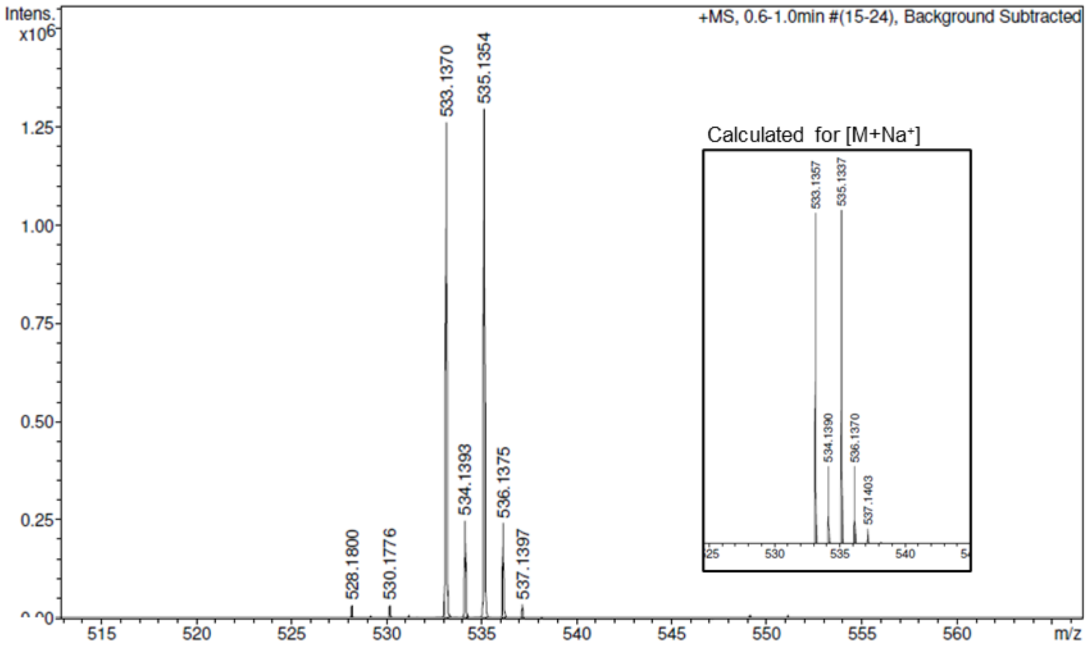

*MS-ESI of **11**.*

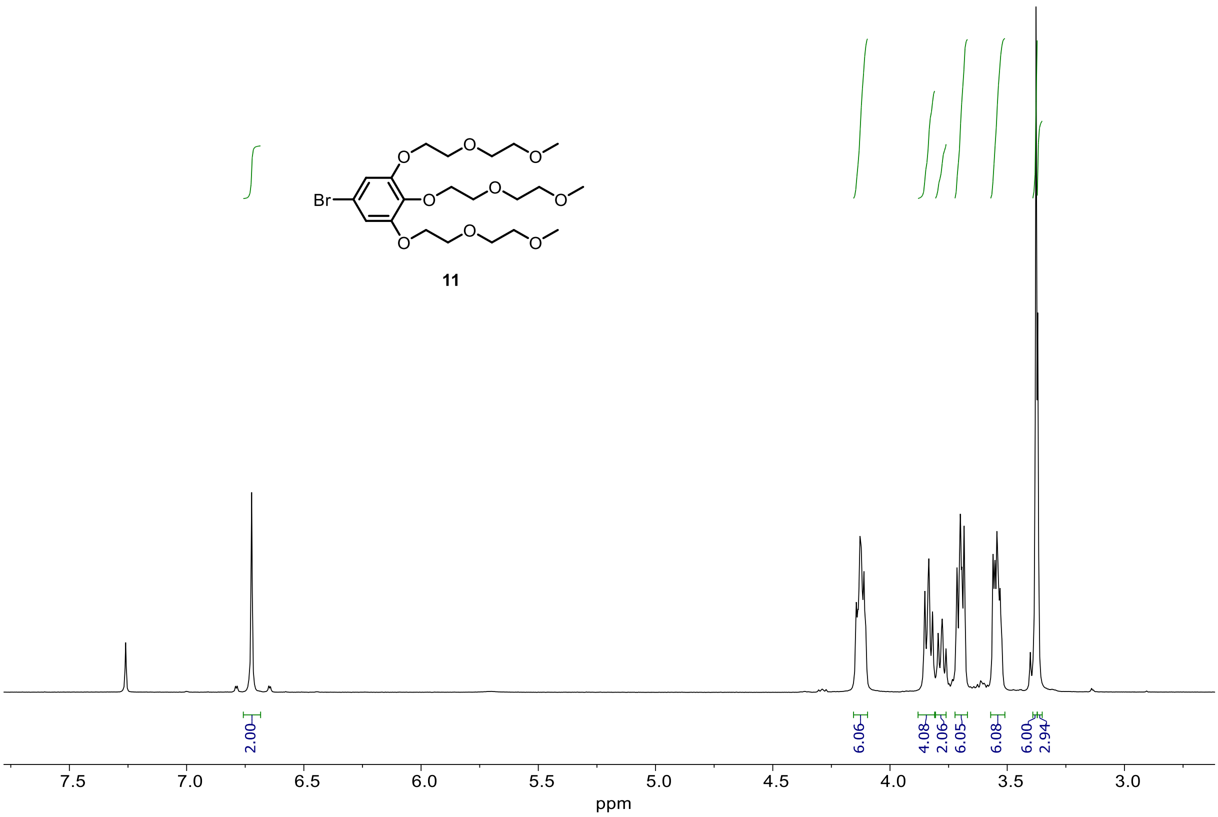

*^{1}H NMR of **11** in $CDCl_3$.*

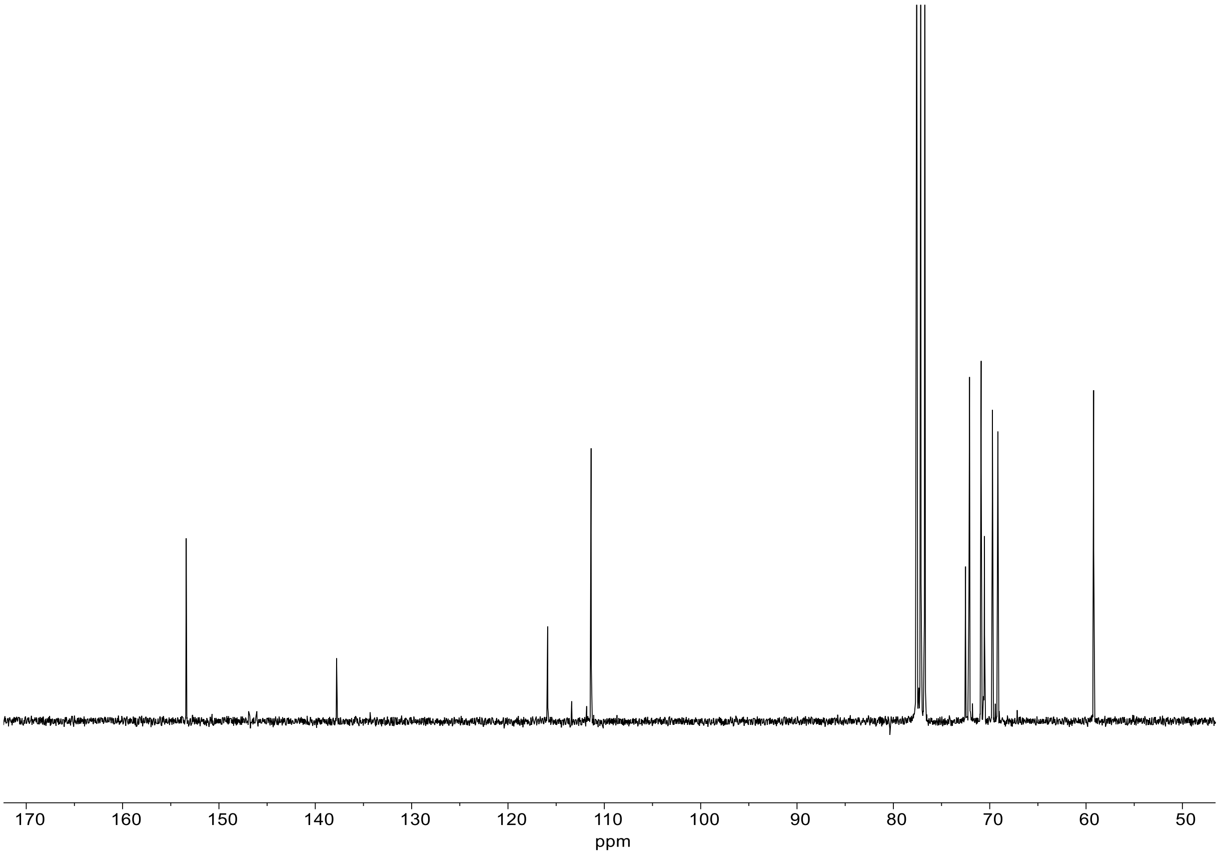

*^{13}C NMR of **11** in $CDCl_3$.*

*Synthesis of 5-bromo-1,2,3-tris(2-methoxyethoxy)benzene (**12**):* **6** (150 mg, 732 µmol) and **9** (590 mg, 2.56 mmol) were dissolved in dry DMF (10 mL). The mixture was subjected to five vacuum/argon cycles. K_2CO_3 (607 mg, 4.39 mmol) was added and the reaction was stirred at 90 °C for 2 days. After cooling to room temperature, water (30 mL) was added and the solution was extracted with DCM (3 × 30 mL). The combined organic layers were then washed with water (3 x 15 mL) and dried over Na_2SO_4. The product was purified by column chromatography (silica, neat DCM to DCM/Et_2O 67:33). Product **12** was obtained as colorless oil (198 mg, 492 µmol, 67%).

MS-ESI: m/z calculated for $[C_{15}H_{23}O_6BrNa]^+$ $[M+Na^+]$: 401.0570; found 401.0595.

^1H NMR (400 MHz, $CDCl_3$, 298 K): δ (in ppm) 6.74 (s, 2 H, phenyl-*H*), 4.15 – 4.08 (m, 6 H, OCH_2), 3.76 – 3.72 (m, 4 H, OCH_2), 3.71 – 3.66 (m, 2 H, OCH_2), 3.43 (s, 6 H, OCH_3), 3.43 (s, 3 H, OCH_3).
^{13}C NMR (101 MHz, $CDCl_3$, 298 K): δ (in ppm) 153.6, 138.2, 115.9, 111.8, 72.4, 71.9, 71.1, 69.3, 59.3, 59.0.

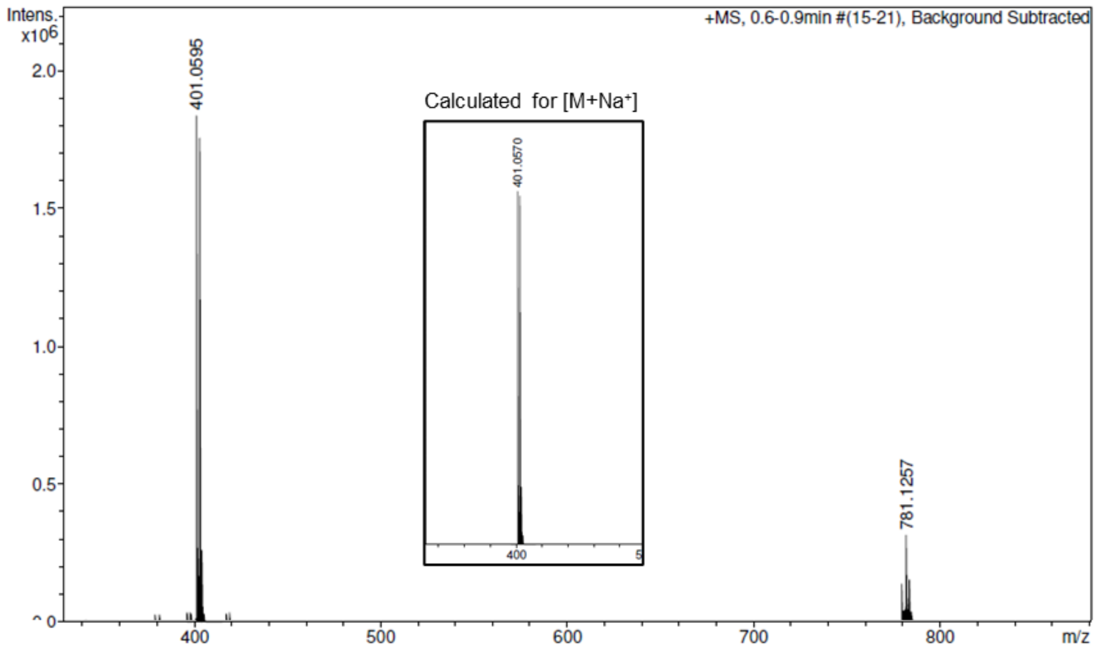

*MS-ESI of **12***.

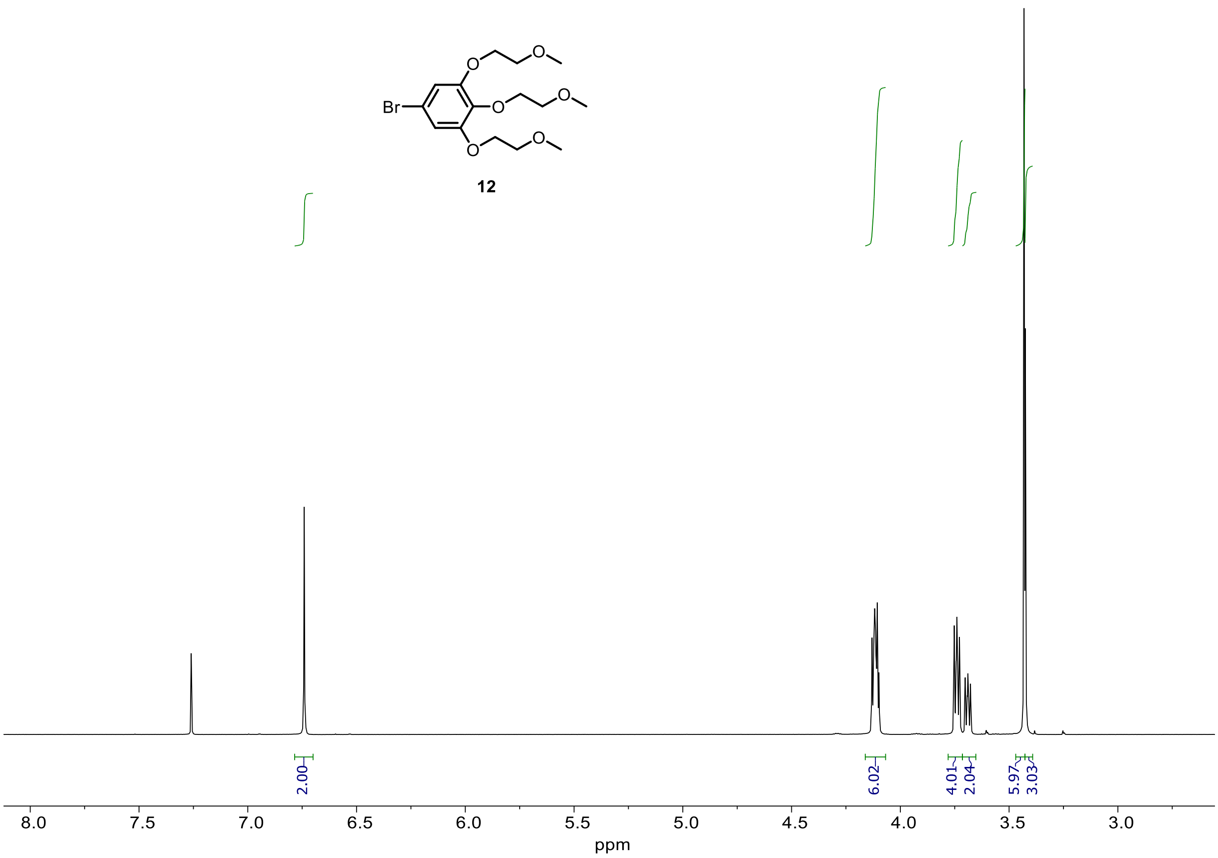

*^{1}H NMR of **12** in $CDCl_3$.*

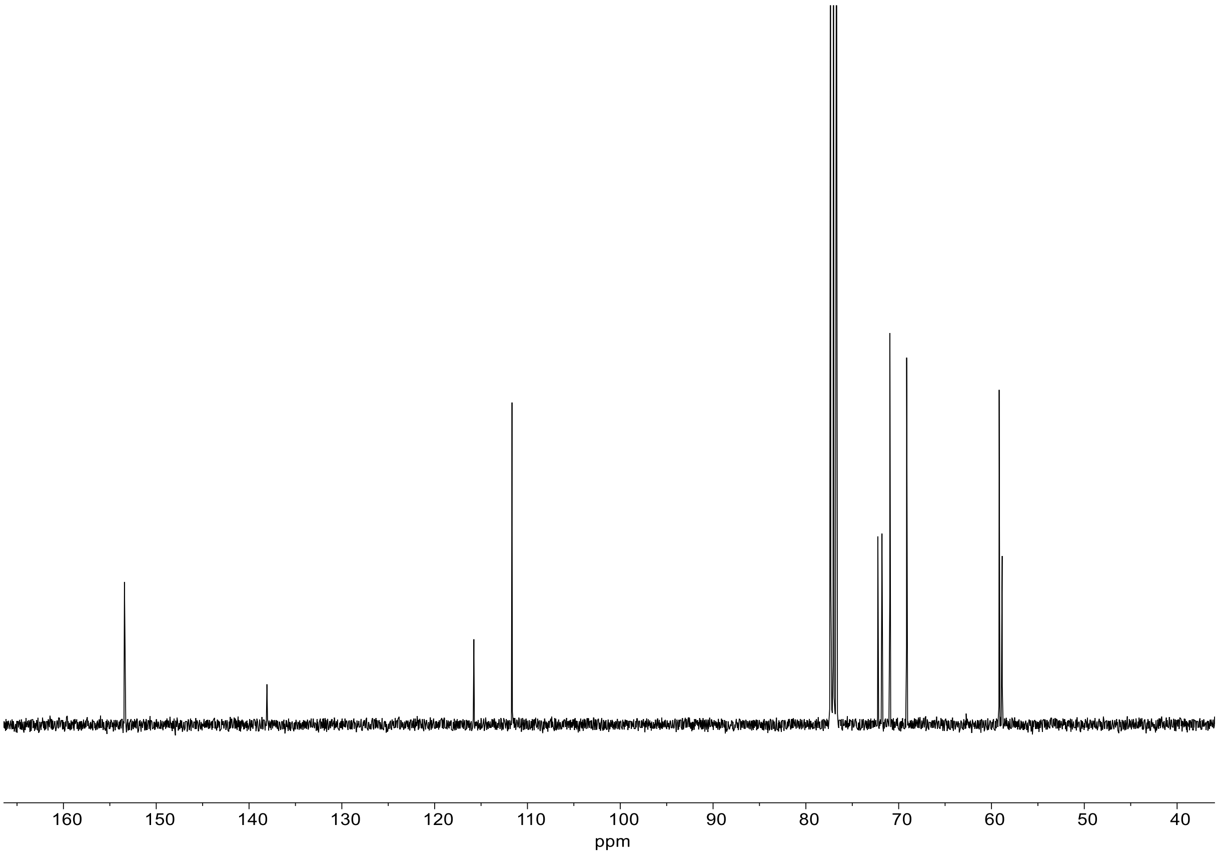

*^{13}C NMR of **12** in $CDCl_3$.*

*Synthesis of trimethyl((3,4,5-tris(2-methoxyethoxy)phenyl)ethynyl)silane (**15**):* **12** (960 mg, 2.53 mmol), $Pd(PPh_3)_4$ (87.8 mg, 75.9 *µ*mol) and CuI (14.5 mg, 75.9 *µ*mol) in degassed NEt_3 (15 mL) were subjected to five vacuum/argon cycles. The reaction was heated to 80 °C and trimethylsilylacetylene (497 mg, 710 *µ*L, 5.06 mmol) was added. The reaction was left to stir at 80 °C for 16 h. After evaporation of the solvent under reduced pressure, the product was purified by column chromatography (silica, neat DCM to DCM/MeOH 98:2) to yield **15** as a pale yellow oil (915 mg, 2.31 mmol, 91%).

MS-ESI: m/z calculated for $[C_{20}H_{32}O_6SiNa]^+$ $[M+Na^+]$: 419.1860; found 419.1876.

^1H NMR (300 MHz, $CDCl_3$, 298 K): δ (in ppm) 6.71 (s, 2H, phenyl-*H*), 4.18 – 4.09 (m, 6H, OCH_2), 3.78 – 3.72 (m, 4H, OCH_2), 3.72 – 3.67 (m, 2H, OCH_2), 3.43 (s, 6H, OCH_3), 3.42 (s, 3H, OCH_3), 0.23 (s, 9H, $SiCH_3$).
^{13}C NMR (75 MHz, $CDCl_3$, 298 K): δ (in ppm) 152.6, 139.8, 118.1, 111.9, 105.1, 93.3, 72.4, 71.9, 71.1, 69.0, 59.3, 59.0, 0.1.

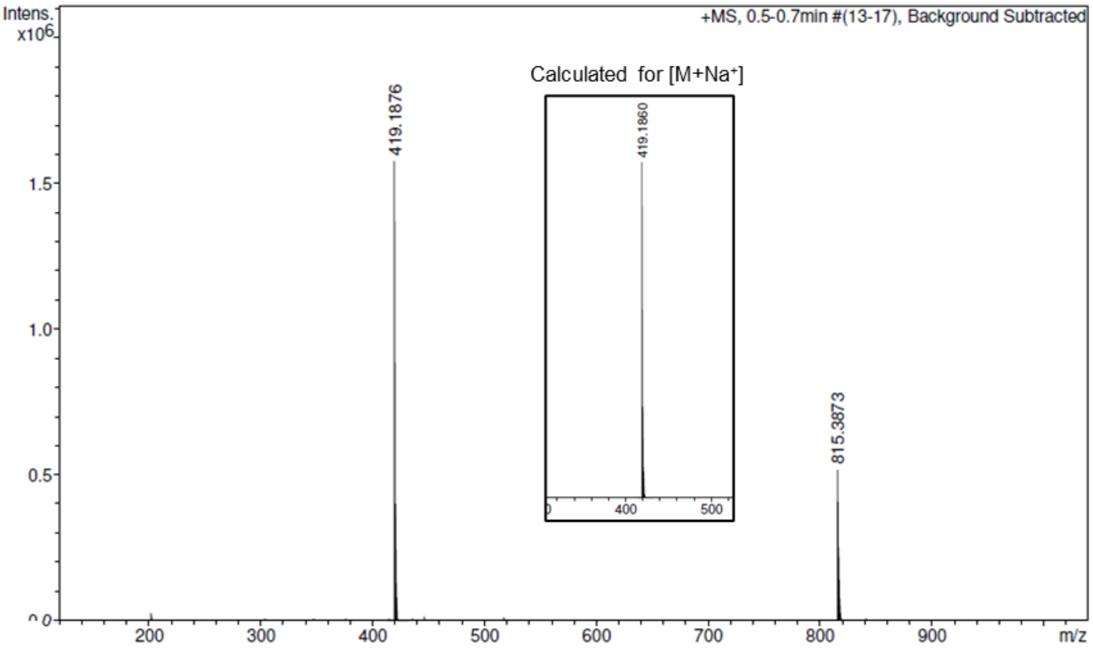

*MS-ESI of **15**.*

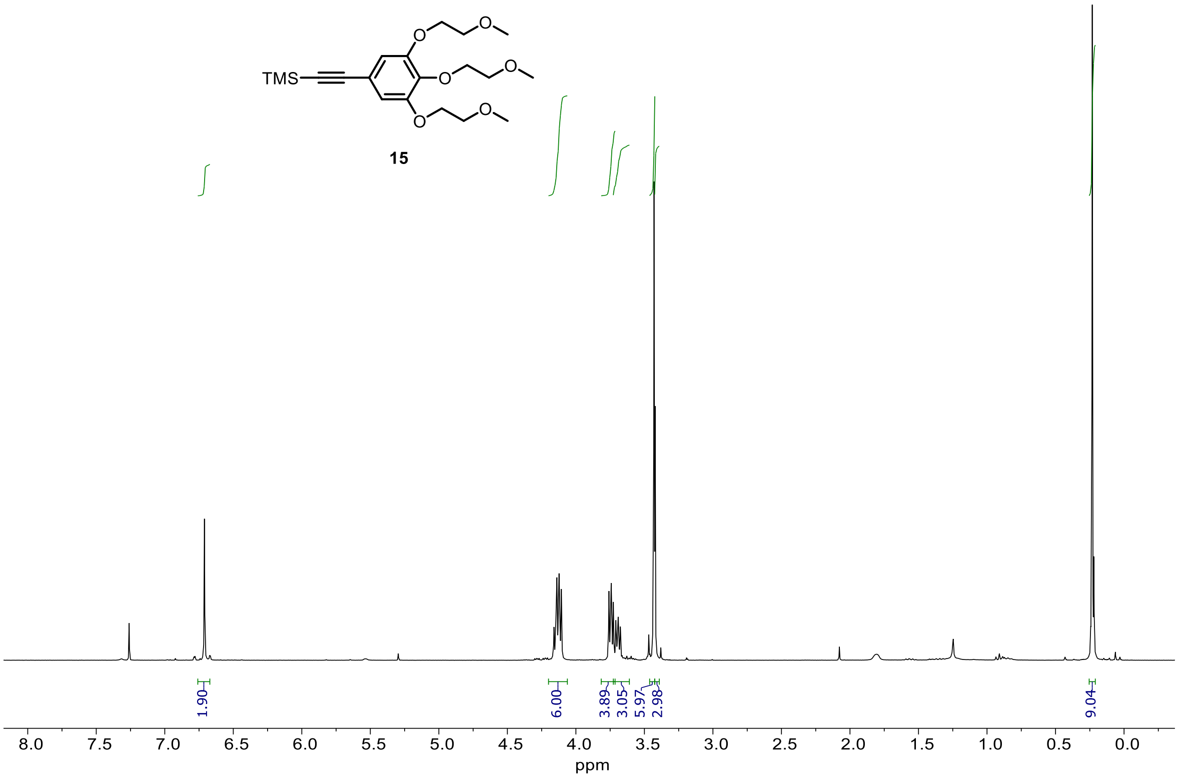

*^{1}H NMR of **15** in $CDCl_3$.*

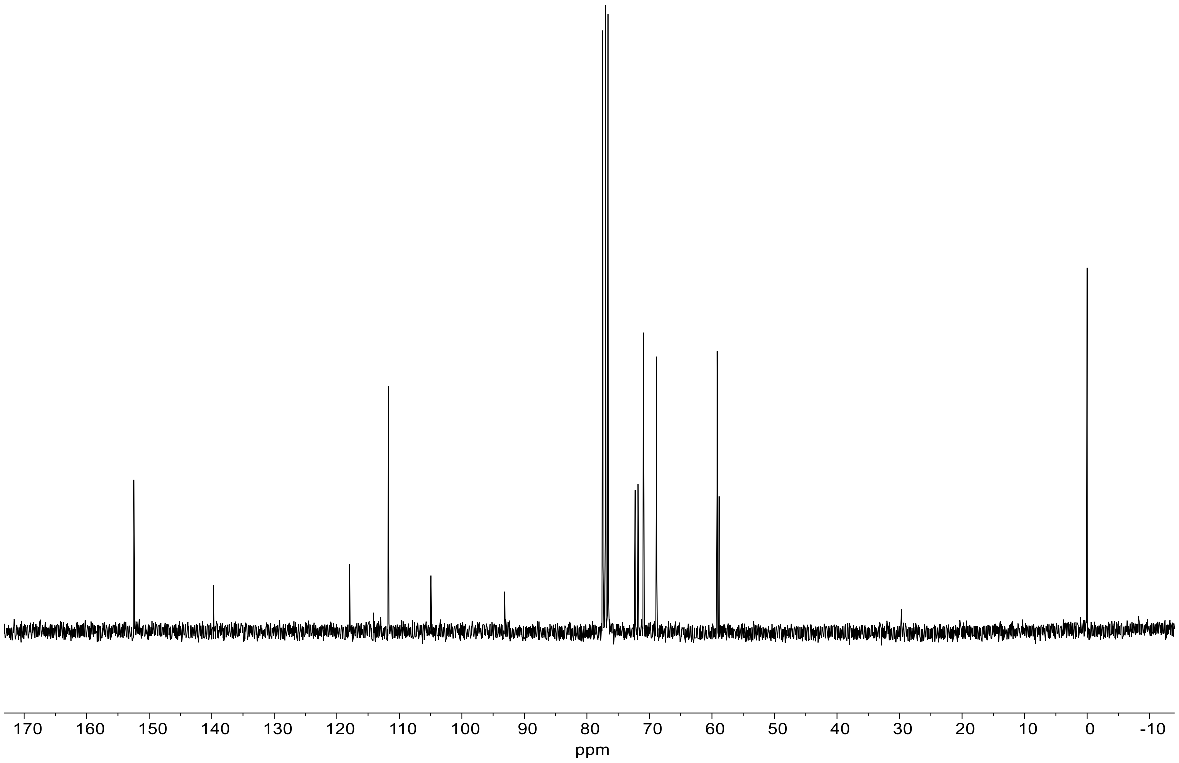

*^{13}C NMR of **15** in $CDCl_3$.*

*Synthesis of 5-ethynyl-1,2,3-tris(2-(2-(2-methoxyethoxy)ethoxy)ethoxy)benzene (**16**):* **13** (864 mg, 1.31 μmol) was dissolved in THF (20 mL) and MeOH (20 mL). K_2CO_3 (452 mg,

3.27 mmol) was added and the mixture was left to stir at room temperature for 1 h. The solvent was removed under reduced pressure. DCM (50 mL) was added and the mixture was washed with water (3 × 40 mL) and brine (1 × 40 mL). The organic layer was then dried over Na_2SO_4. After evaporation of the solvent under reduced pressure, the product was purified by column chromatography (silica, neat DCM to DCM/MeOH 97:3). Product **16** was obtained as yellow oil (675 mg, 1.15 μmol, 88%).

MS-ESI: m/z calculated for $[C_{29}H_{48}O_{12}Na]^+$ $[M+Na^+]$: 611.30380; found 611.30450.

^{1}H NMR (300 MHz, $CDCl_3$, 298 K): δ (in ppm) 6.72 (s, 2H, phenyl-*H*), 4.18 – 4.09 (m, 6H, OCH_2), 3.86 – 3.80 (m, 4H, OCH_2), 3.80 – 3.74 (m, 2H, OCH_2), 3.74 – 3.67 (m, 6H, OCH_2), 3.67 – 3.58 (m, 12H, OCH_2), 3.58 – 3.50 (m, 6H, OCH_2), 3.36 (s, 9H, OCH_3), 2.99 (s, 1H, CC*H*).

^{13}C NMR (75 MHz, $CDCl_3$, 298 K): δ (in ppm) 152.5, 139.8, 116.9, 111.8, 83.7, 76.3, 72.5, 72.2, 70.9, 70.8, 70.8, 70.7, 70.6, 70.6, 69.7, 69.0, 59.2.

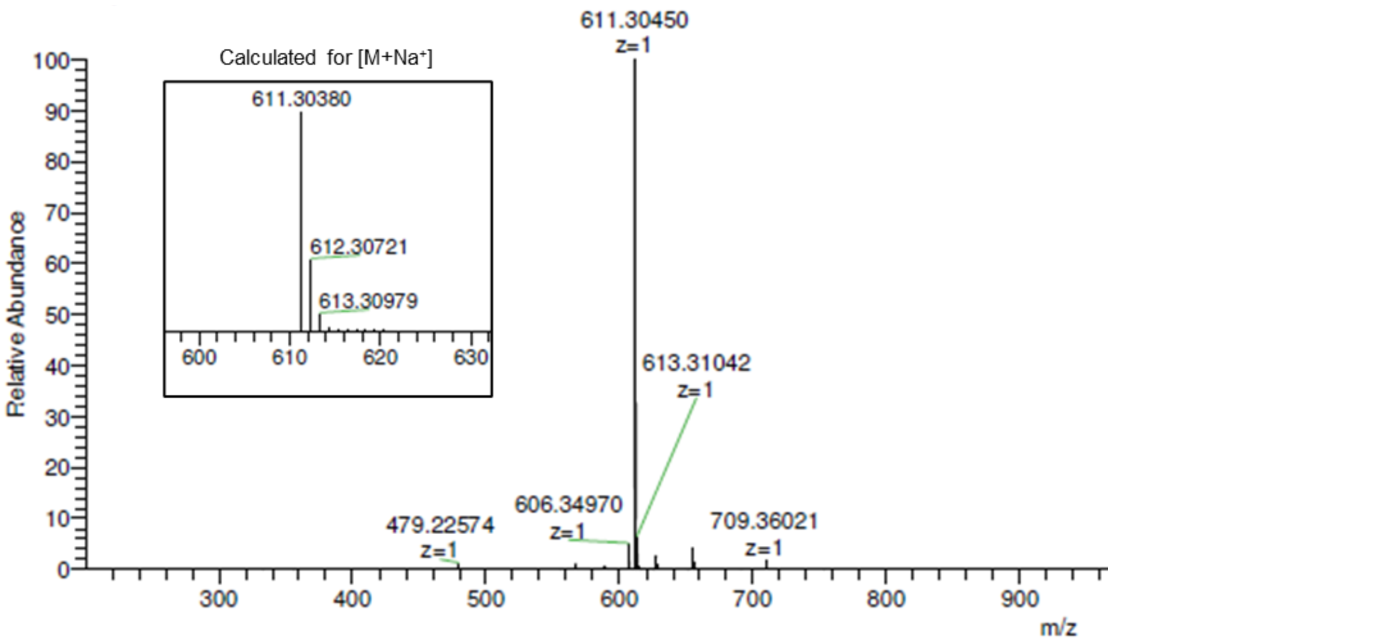

*MS-ESI of **16**.*

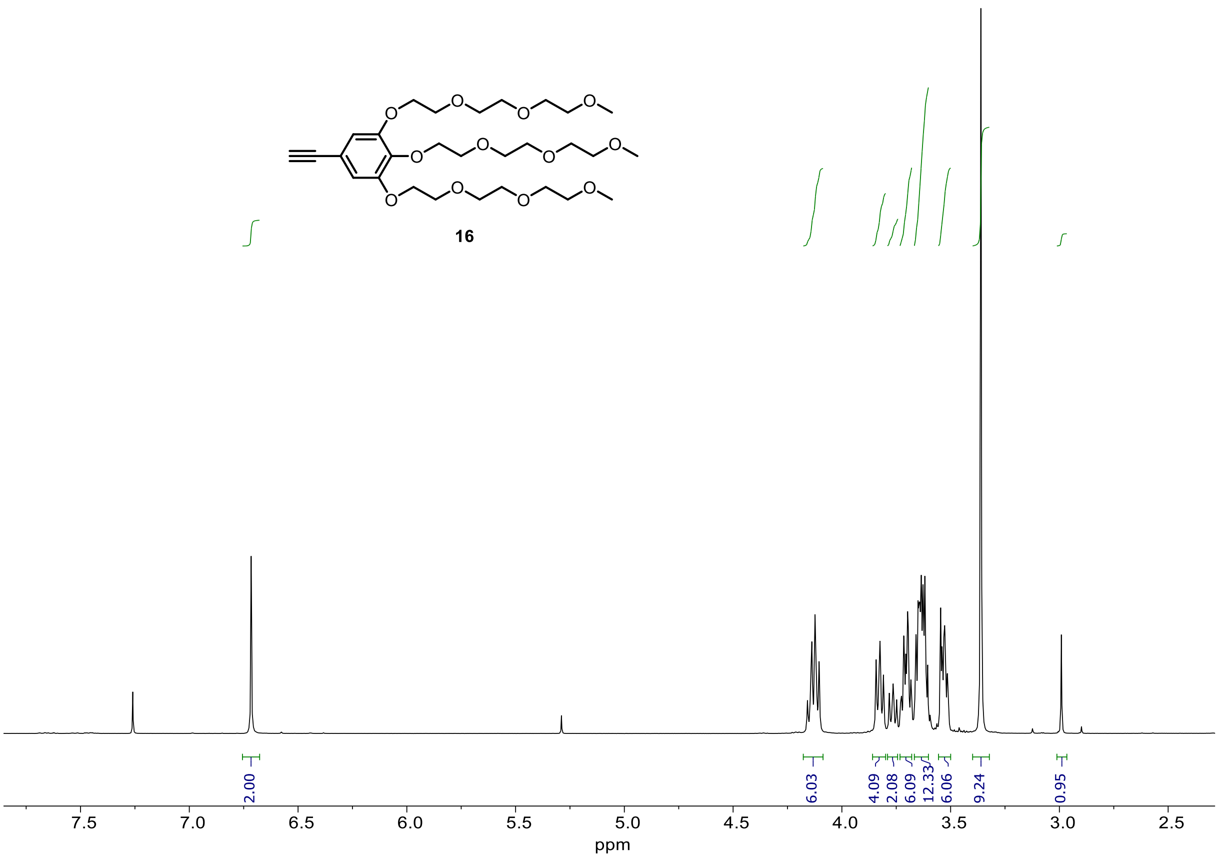

*^{1}H NMR of **16** in $CDCl_3$.*

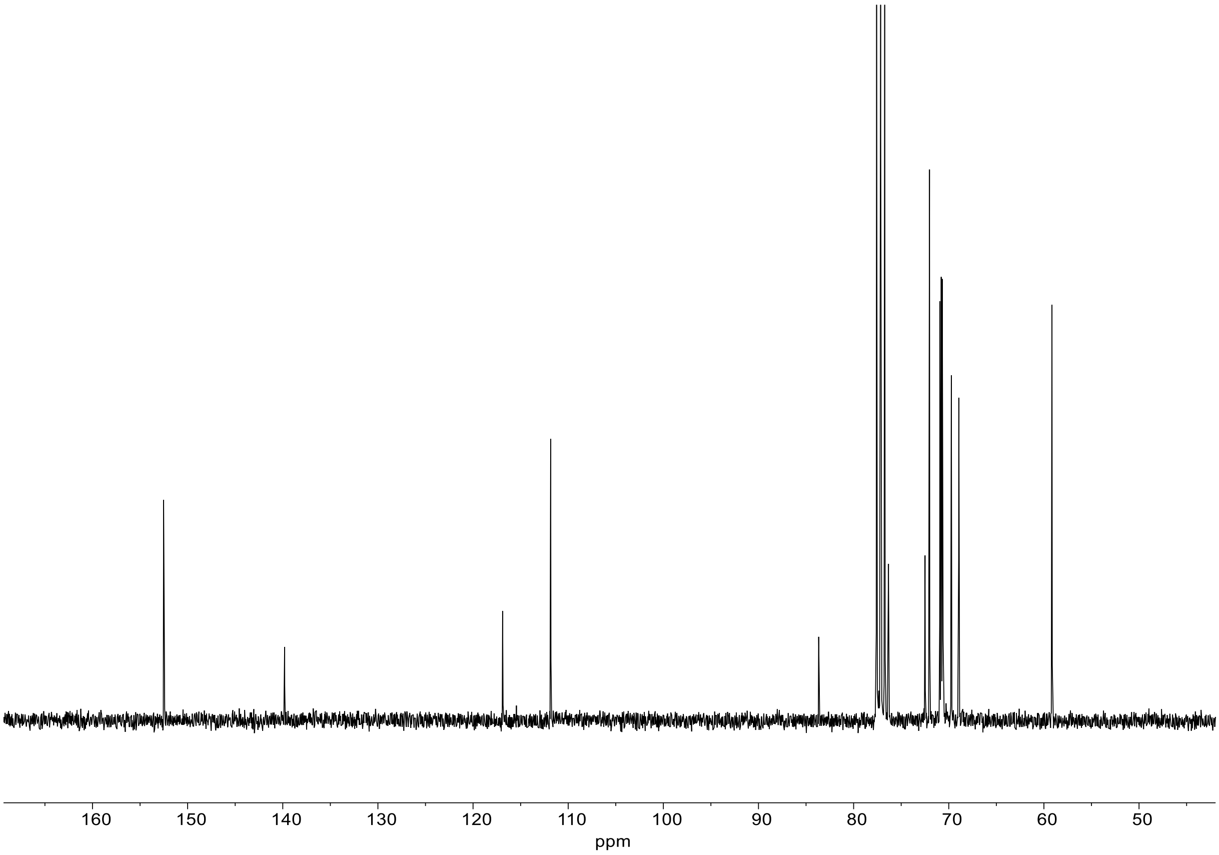

*^{13}C NMR of **16** in $CDCl_3$.*

*Synthesis of 5-ethinyl-1,2,3-tris(2-methoxyethoxy)benzene (**18**):* **15** (843 mg, 2.13 mmol) was dissolved in THF (15 mL) and MeOH (15 mL). K_2CO_3 (734 mg, 5.31 mmol) was added and the mixture was left to stir at room temperature for 1 h. The solvent was removed under reduced pressure. DCM (70 mL) was added and the mixture was washed with water (3 × 50 mL) and brine (1 × 50 mL). The organic layer was then dried over Na_2SO_4. After evaporation of the solvent the product was purified by column chromatography (neat DCM to DCM/MeOH 97:3) to afford **18** as a yellow oil (509 mg, 1.57 mmol, 74%).

MS-ESI: m/z calculated for $[C_{17}H_{24}O_6Na]^+$ $[M+Na^+]$: 347.1465; found 347.1483.

^{1}H NMR (300 MHz, $CDCl_3$, 298 K): δ (in ppm) 6.74 (s, 2H, phenyl-*H*), 4.18 – 4.10 (m, 6H, OCH_2), 3.77 – 3.72 (m, 4H, OCH_2), 3.72 – 3.67 (m, 2H, OCH_2), 3.43 (s, 6H, OCH_3), 3.43 (s, 3H, OCH_3), 3.00 (s, 1H, CC*H*).
^{13}C NMR (75 MHz, $CDCl_3$, 298 K): δ (in ppm) 152.7, 140.1, 117.0, 112.2, 83.7, 76.4, 72.4, 72.0, 71.1, 69.1, 59.3, 59.0.

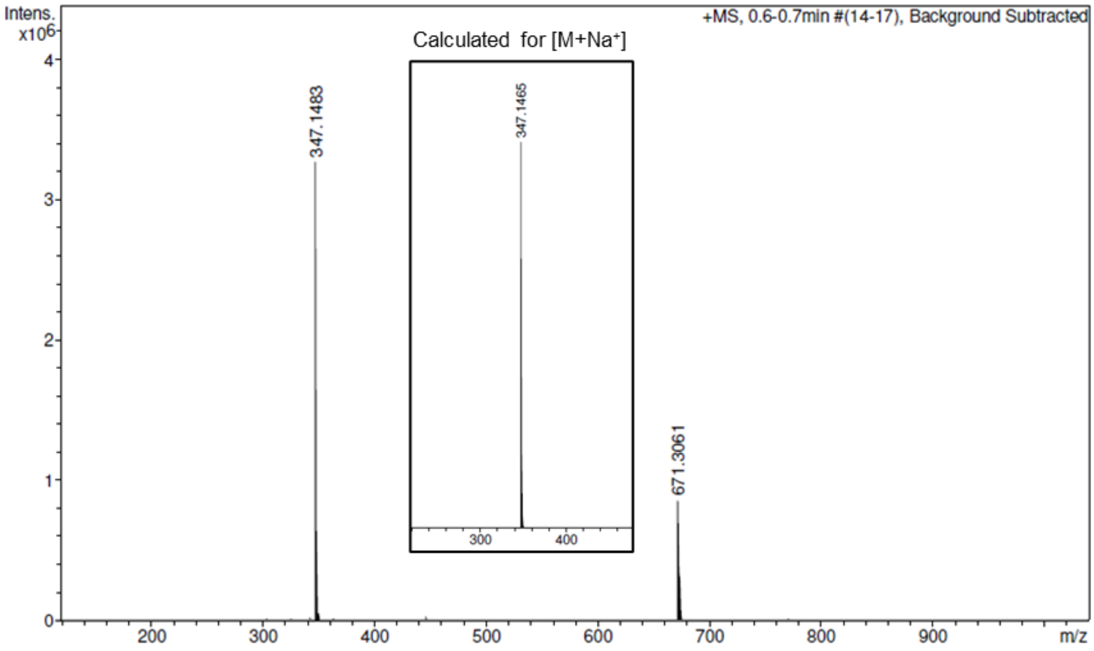

*MS-ESI of **18**.*

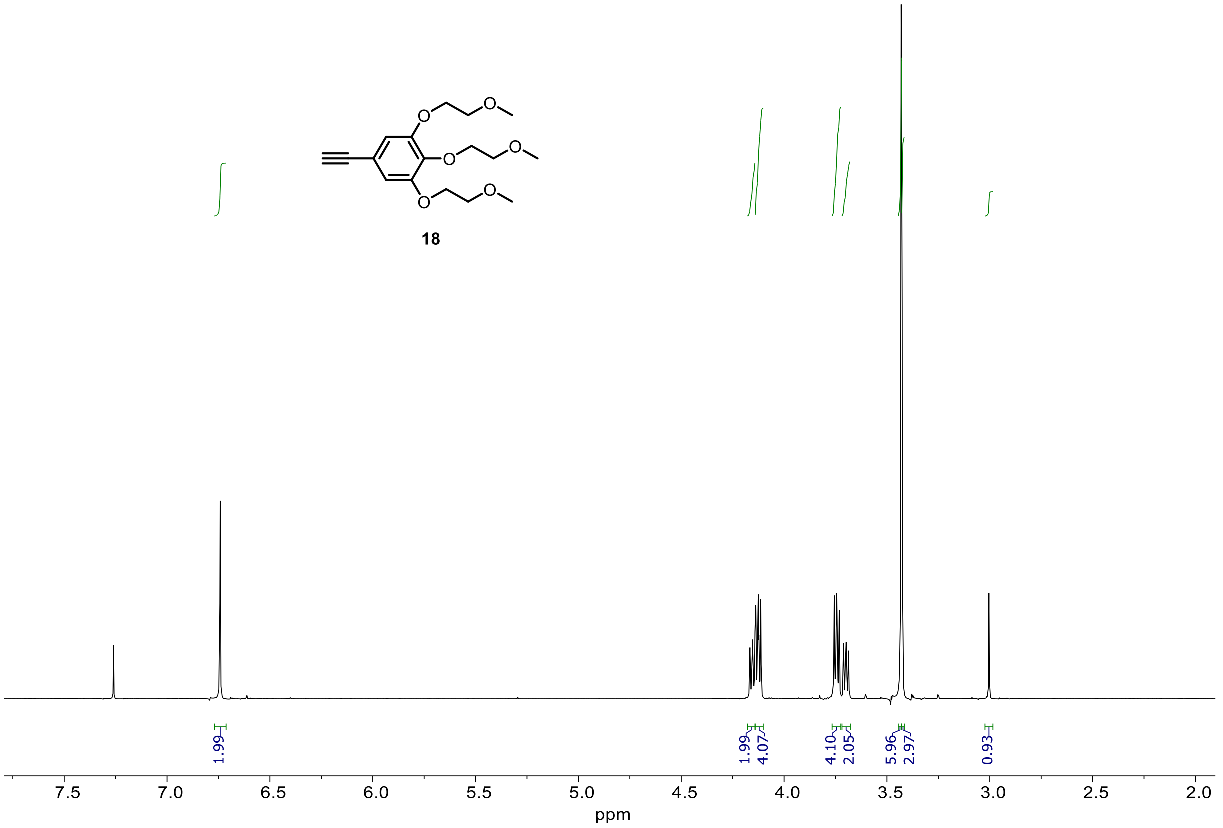

^{1}H NMR of **18** in $CDCl_3$.

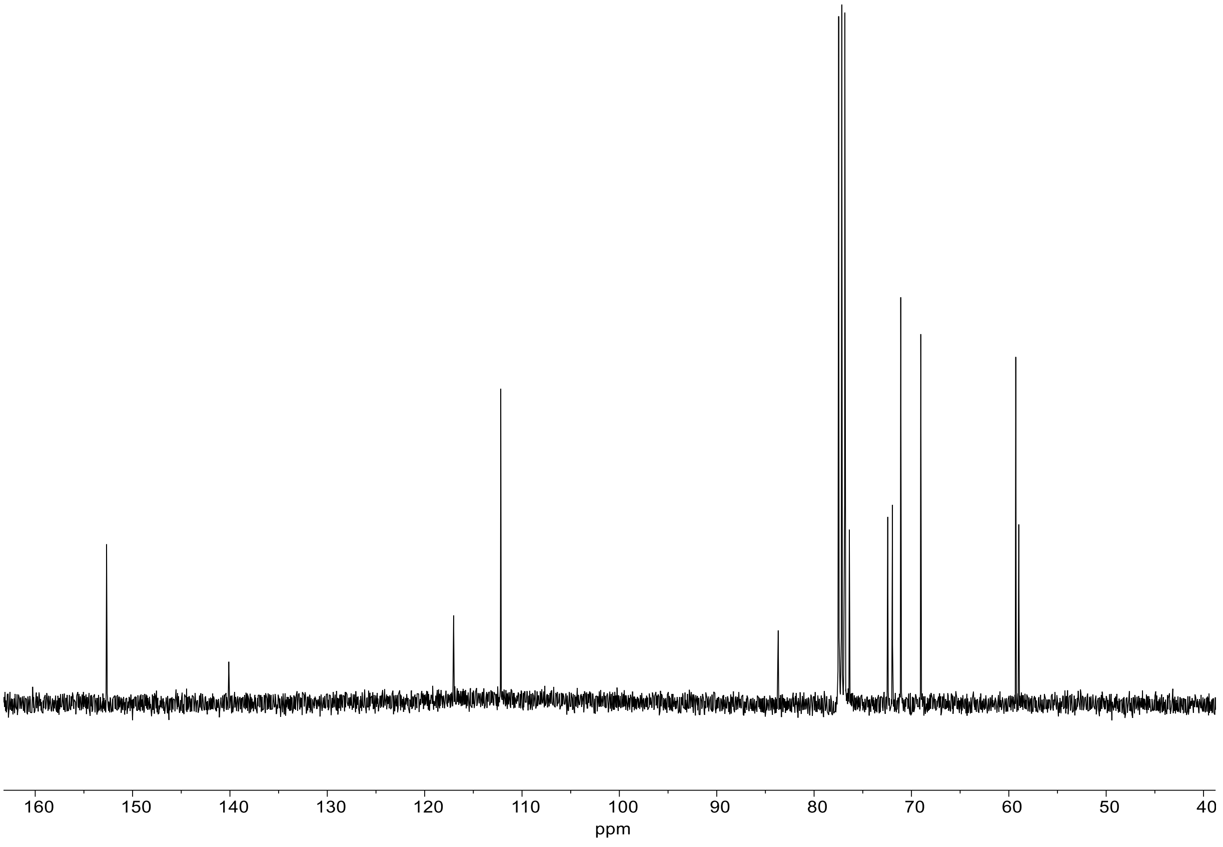

*^{13}C NMR of **18** in $CDCl_3$.*

*Synthesis of 4-((3,4,5-tris(2-(2-(2-methoxyethoxy)ethoxy)ethoxy)phenyl)ethynyl)pyridine (**19**):* 4-Iodopyridine (147 mg, 717 mmol), $Pd(PPh_3)_4$ (24.9 mg, 21.5 μmol) and CuI (4.1 mg, 22 μmol) in degassed NEt_3 (15 mL) were subjected to five vacuum/argon cycles and then heated to 80 °C. **16** (422 mg, 717 mmol) in toluene (5 mL) was added. The mixture was left to stir at 80 °C for 16 h. After evaporation of the solvent under reduced pressure, the residue was purified by column chromatography (silica, neat DCM to DCM/MeOH 97:3) to afford **19** as a pale yellow oil (410 mg, 615 μmol, 86%).

MS-ESI: m/z calculated for $[C_{29}H_{48}O_{12}Na]^+$ $[M+Na^+]$: 688.3303; found 688.3313.

^{1}H NMR (300 MHz, $CDCl_3$, 298 K): δ (in ppm) 8.59 (m, 2H, pyridine-*H*), 7.36 (m, 2H, pyridine-*H*), 6.80 (s, 2H, phenyl-*H*), 4.23 – 4.15 (m, 6H, OCH_2), 3.89 – 3.83 (m, 4H, OCH_2), 3.80 – 3.75 (m, 2H, OCH_2), 3.75 – 3.69 (m, 6H, OCH_2), 3.69 – 3.61 (m, 12H, OCH_2), 3.57 – 3.51 (m, 6H, OCH_2), 3.37 (s, 3H, OCH_3), 3.37 (s, 6H, OCH_3).

^{13}C NMR (75 MHz, $CDCl_3$, 298 K): δ (in ppm) 152.7, 149.3, 140.3, 132.2, 125.7, 116.7, 111.7, 94.9, 85.8, 72.6, 72.0, 71.0, 70.8, 70.8, 70.7, 70.7, 70.6, 69.7, 69.0, 59.1.

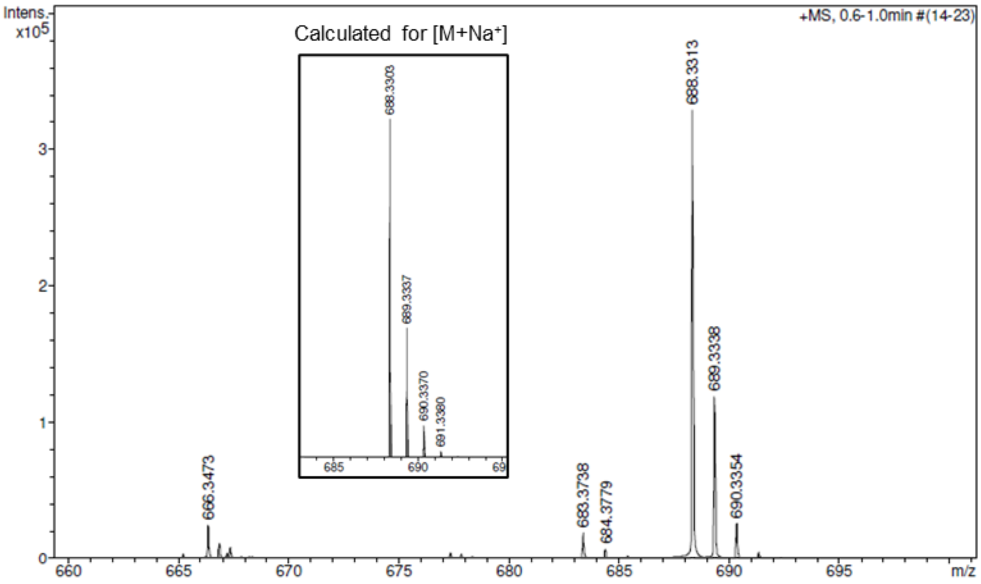

*MS-ESI of **19**.*

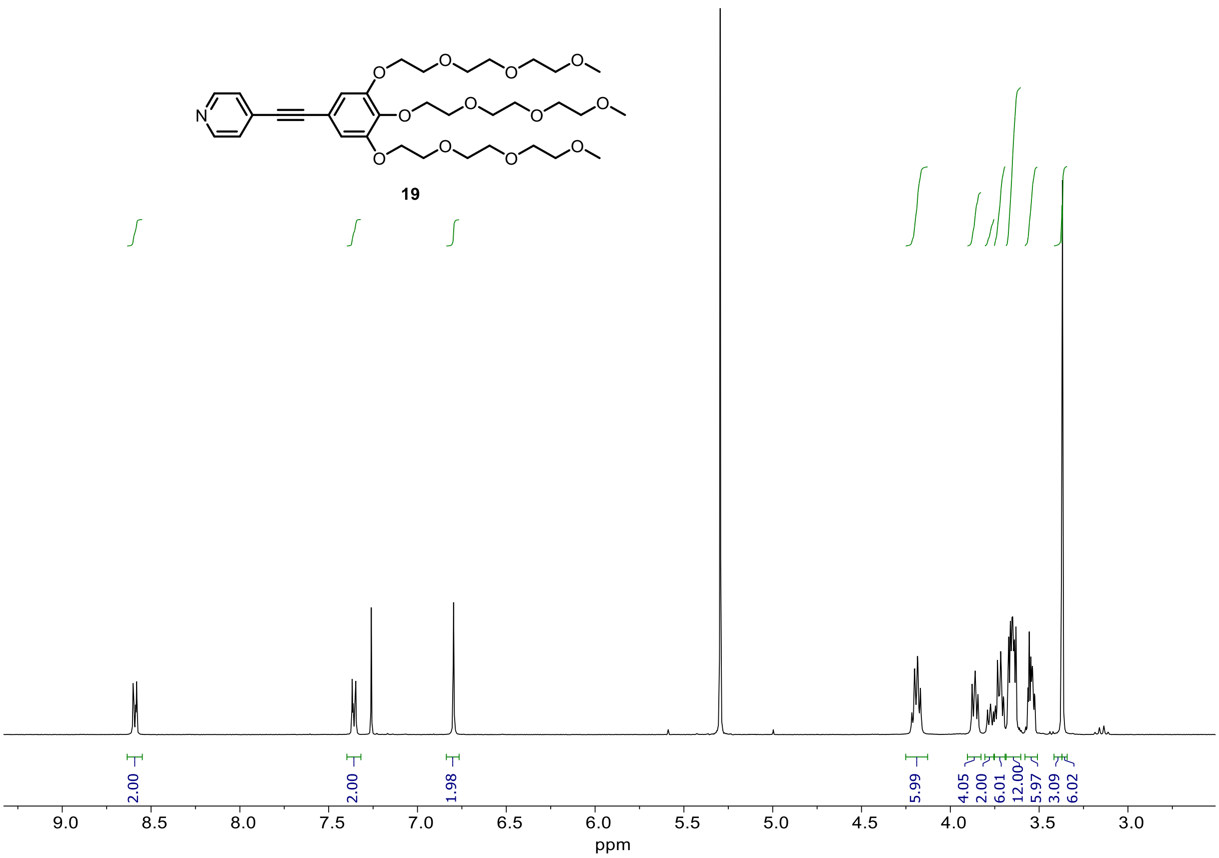

^{1}*H NMR of* ***19*** *in* $CDCl_3$*.*

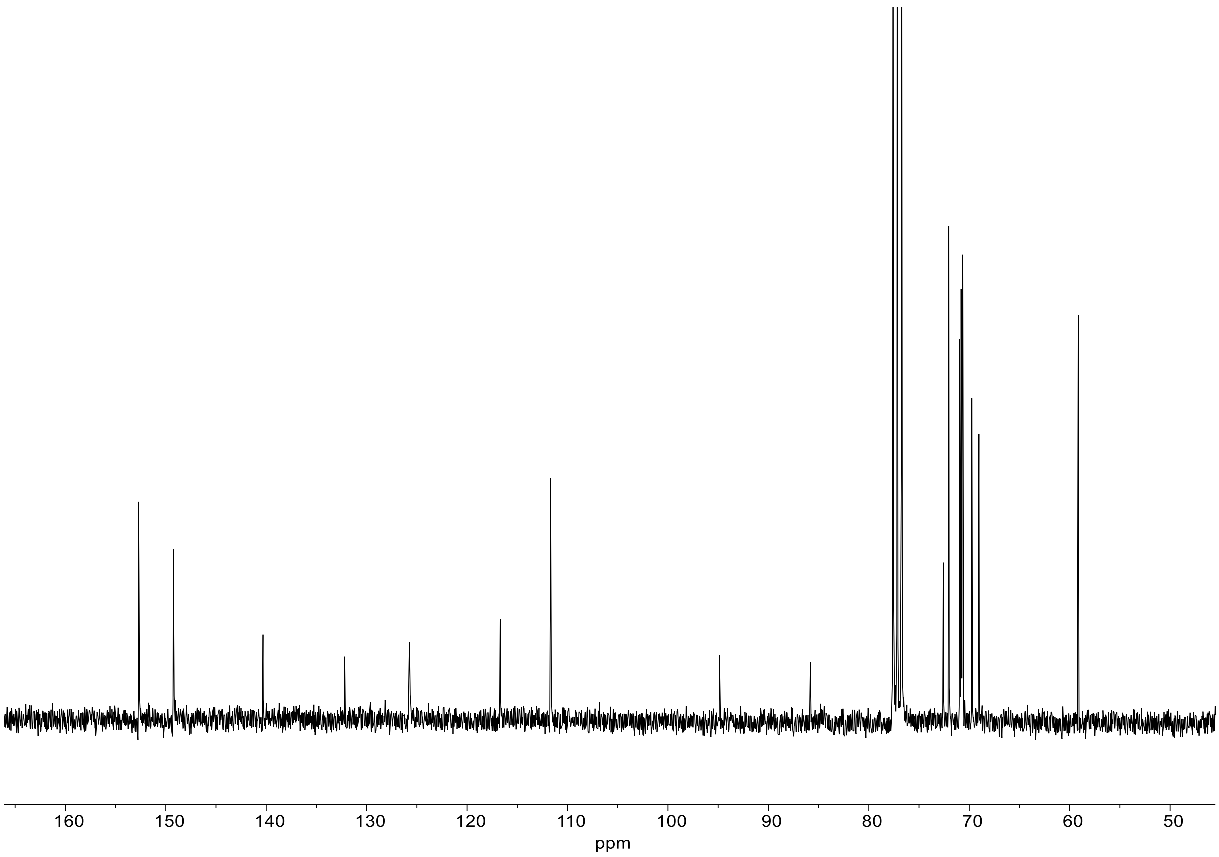

^{13}*C NMR of* ***19*** *in CDCl$_3$.*

*Synthesis of 4-((3,4,5-tris(2-(2-methoxyethoxy)ethoxy)phenyl)ethynyl)pyridine (**20**):* 4-Iodopyridine (249 mg, 1.22 mmol), $Pd(PPh_3)_4$ (42.1 mg, 36.5 μmol) and CuI (7.0 mg, 36.5 μmol) in degassed NEt_3 (10 mL) were subjected to five vacuum/argon cycles and then heated to 80 °C. **17** (555 mg, 1.22 mmol) in toluene (5 mL) was added. The mixture was left to stir at 80 °C for 16 h. After evaporation of the solvent under reduced pressure, the residue was purified by column chromatography (silica, neat DCM to DCM/MeOH 97:3) to afford **20** as a pale brown oil (432 mg, 810 μmol, 67%).

MS-ESI: m/z calculated for $[C_{28}H_{39}NO_9Na]^+$ $[M+Na^+]$: 556.25170; found 556.25185.

^{1}H NMR (300 MHz, $CDCl_3$, 298 K): δ (in ppm) 8.60 (unresolved m, 2H, pyridine-*H*), 7.36 (m, 2H, pyridine-*H*), 6.79 (s, 2H, phenyl-*H*), 4.23 – 4.14 (m, 6H, OCH_2), 3.90 – 3.84 (m, 4H, OCH_2), 3.84 – 3.77 (m, 2H, OCH_2), 3.75 – 3.68 (m, 6H, OCH_2), 3.59 – 3.52 (m, 6H, OCH_2), 3.38 (s, 6H, OCH_3), 3.38 (s, 3H, OCH_3).
^{13}C NMR (75 MHz, $CDCl_3$, 298 K): δ (in ppm) 152.7, 149.8, 140.1, 131.6, 125.7, 116.9, 111.6, 94.3, 85.9, 72.7, 72.2, 72.1, 70.9, 70.7, 70.6, 69.8, 69.0, 59.2, 59.2.

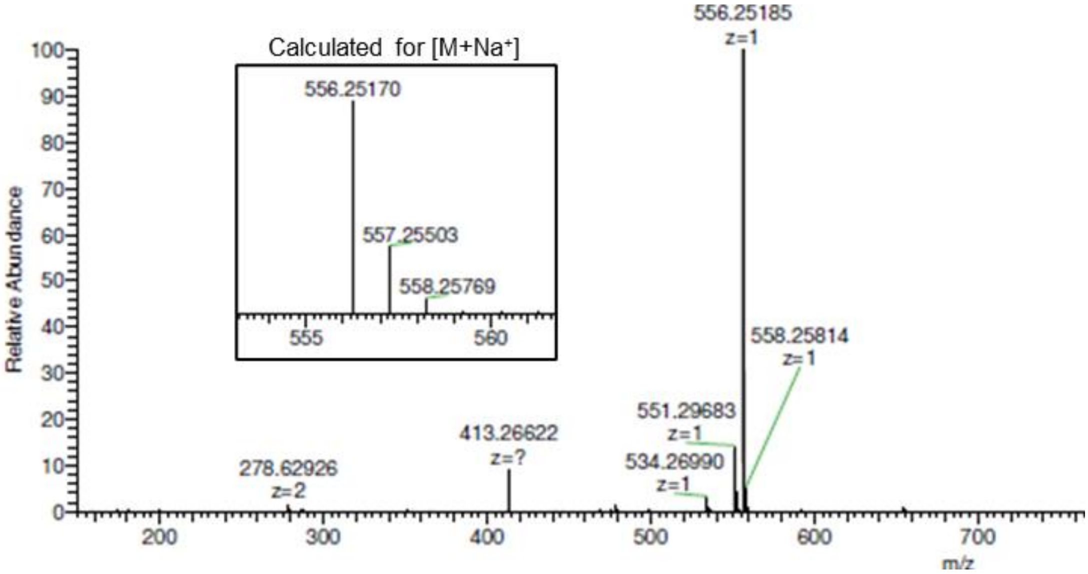

*MS-ESI of **20**.*

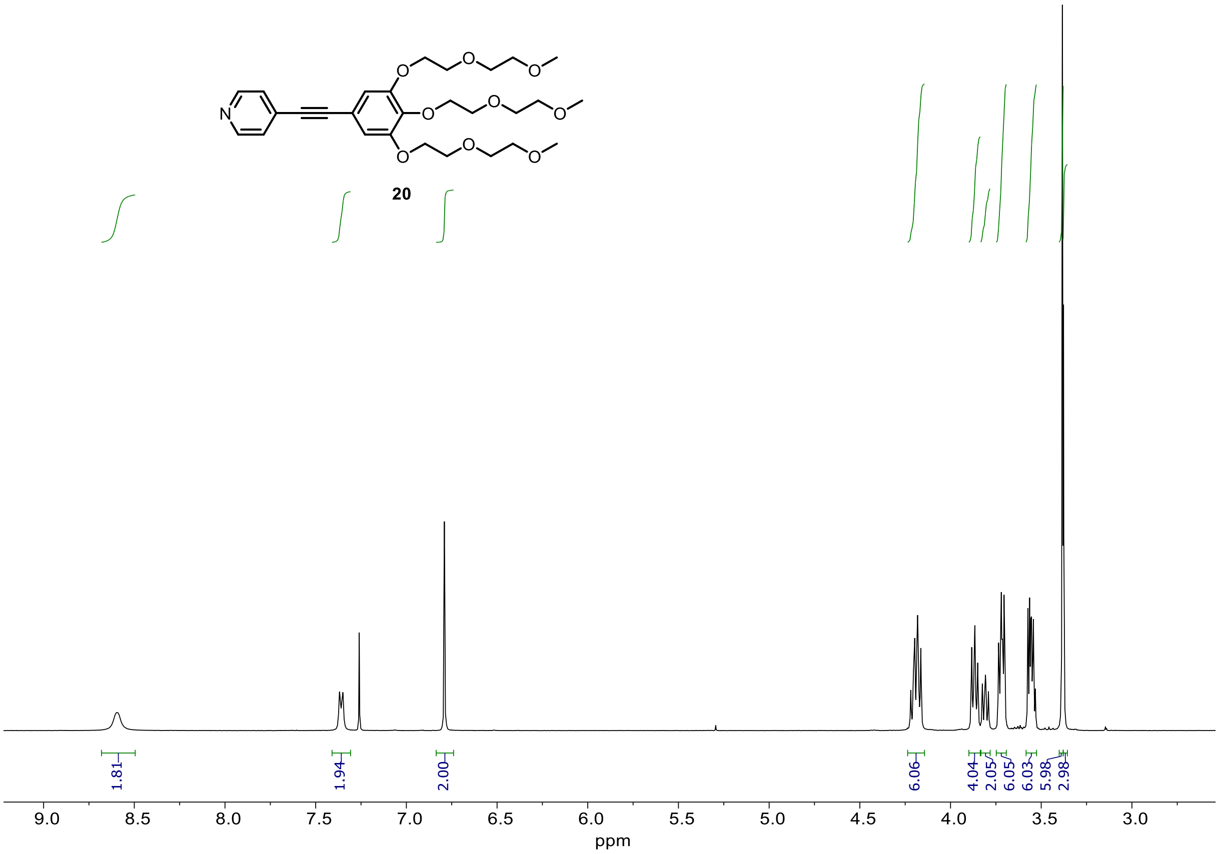

*^{1}H NMR of **20** in $CDCl_3$.*

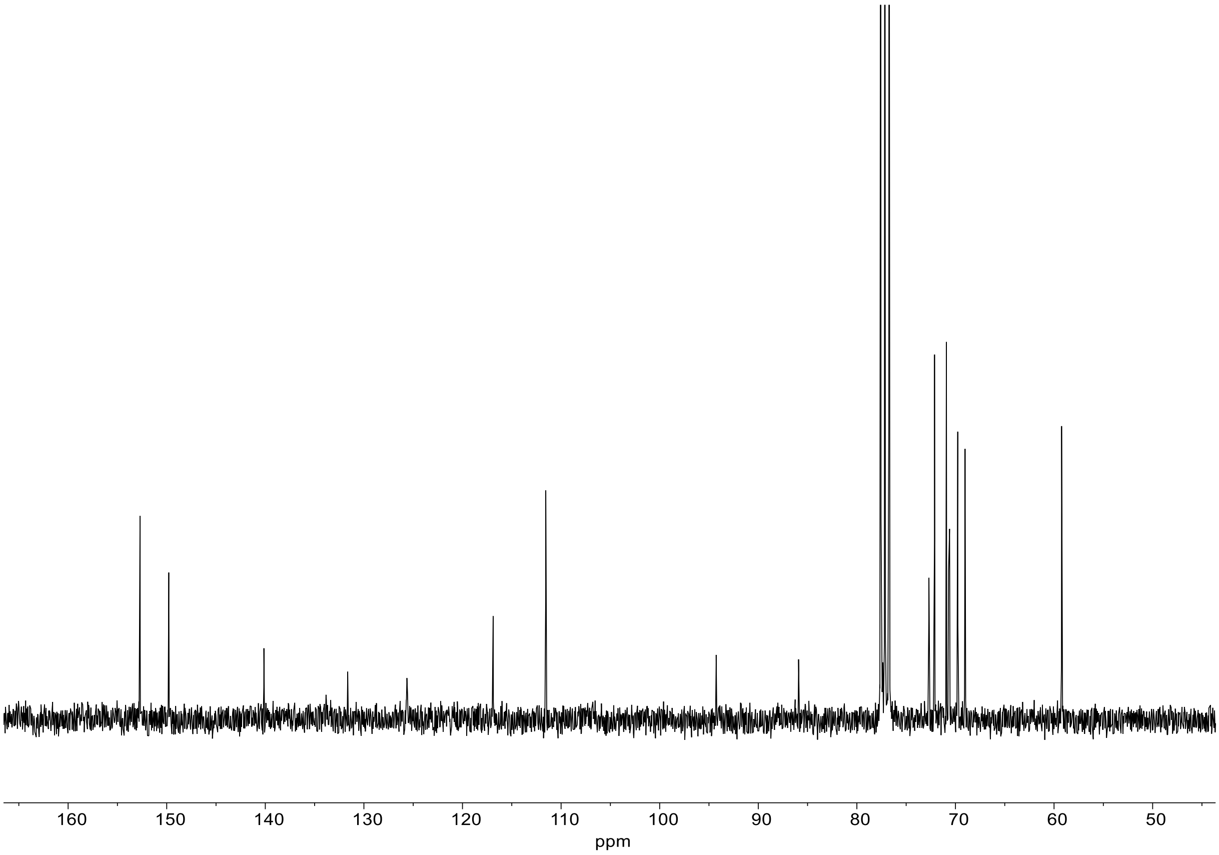

*^{13}C NMR of **20** in $CDCl_3$.*

*Synthesis of 4-((3,4,5-tris(2-methoxyethoxy)phenyl)ethynyl)pyridine (**21**):* 4-Iodopyridine (354 mg, 1.73 mmol), $Pd(PPh_3)_4$ (54.4 mg, 47.1 μmol) and CuI (9.0 mg, 47 μmol) in degassed NEt_3 (15 mL) were subjected to five vacuum/argon cycles and then heated to 80 °C. **18** (509 mg, 1.57 mmol) in toluene (10 mL) was added. The mixture was left to stir at 80 °C for 16 h. After evaporation of the solvent under reduced pressure, the residue was purified by column chromatography (silica, neat DCM to DCM/MeOH 98:2). Product **21** was obtained as pale yellow solid (571 mg, 1.42 mmol, 91%).

MS-ESI: m/z calculated for $[C_{22}H_{27}NO_6Na]^+$ $[M+Na^+]$: 424.1731; found 424.1747.

^{1}H NMR (300 MHz, $CDCl_3$, 298 K): δ (in ppm) 8.59 (unresolved m, 2H, pyridine-*H*), 7.36 (m, 2H, pyridine-*H*), 6.80 (s, 2H, phenyl-*H*), 4.21 – 4.11 (m, 6H, OCH_2), 3.80 – 3.74 (m, 4H, OCH_2), 3.74 – 3.68 (m, 2H, OCH_2), 3.45 (s, 6H, OCH_3), 3.44 (s, 3H, OCH_3).
^{13}C NMR (75 MHz, $CDCl_3$, 298 K): δ (in ppm) 152.8, 149.8, 140.4, 131.5, 125.6, 116.9, 111.9, 94.1, 85.9, 72.5, 72.0, 71.1, 69.1, 59.3, 59.0.

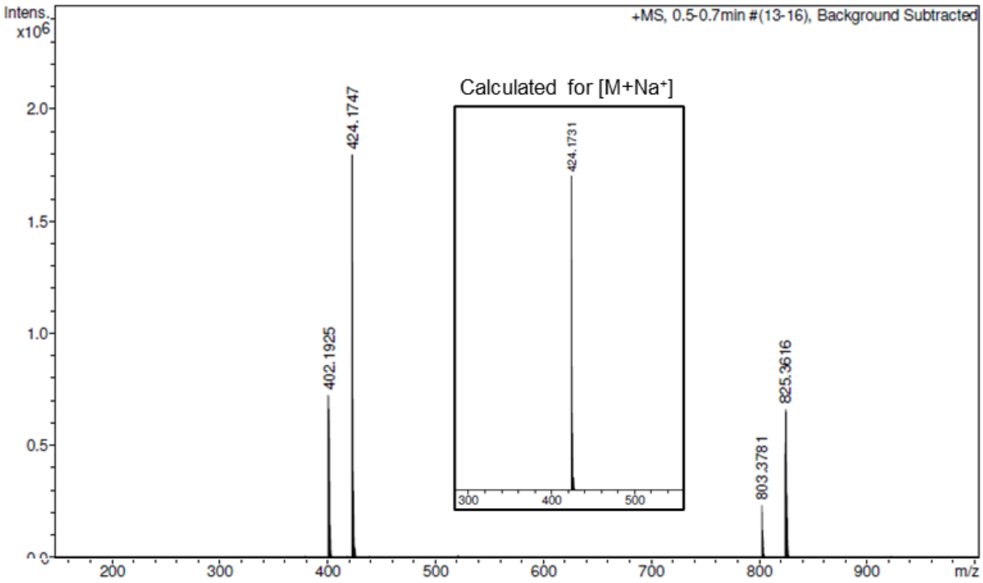

*MS-ESI of **21**.*

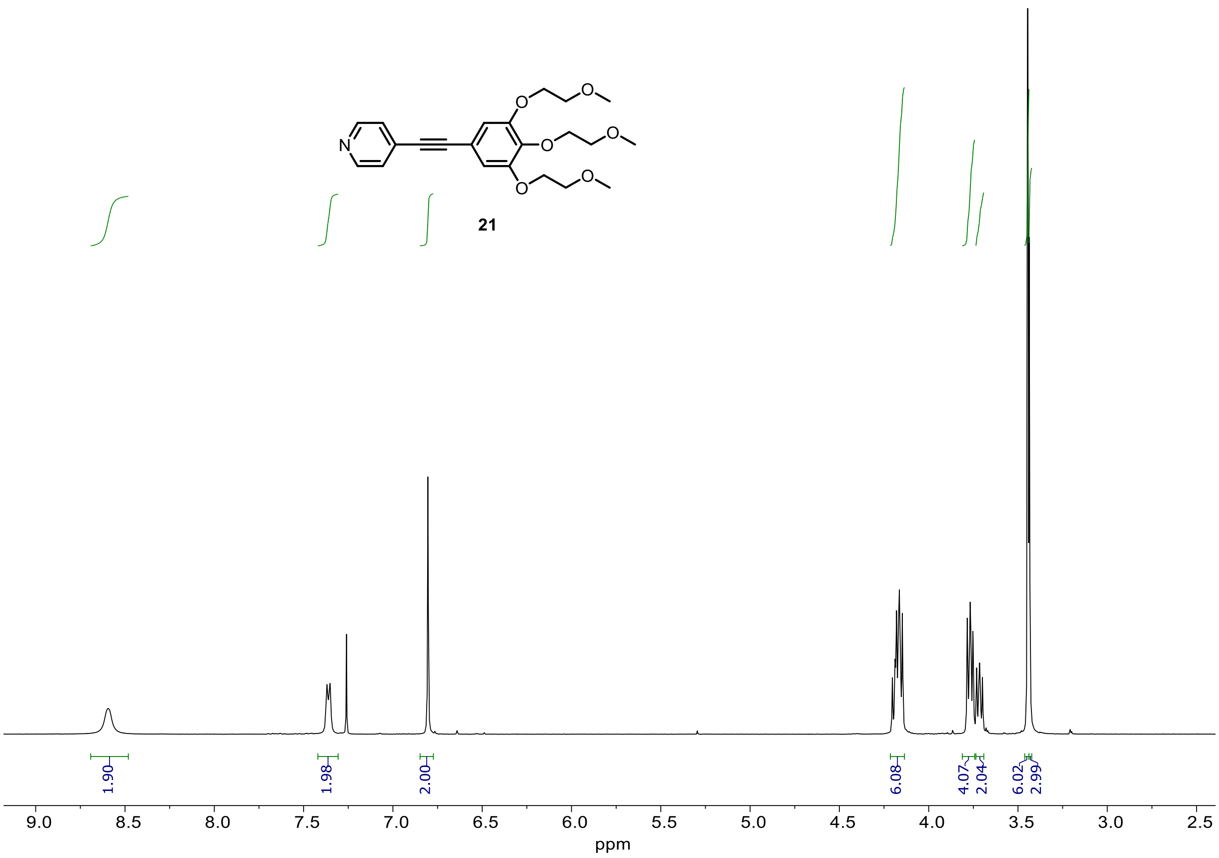

^{1}H NMR of ***21*** *in CDCl$_3$.*

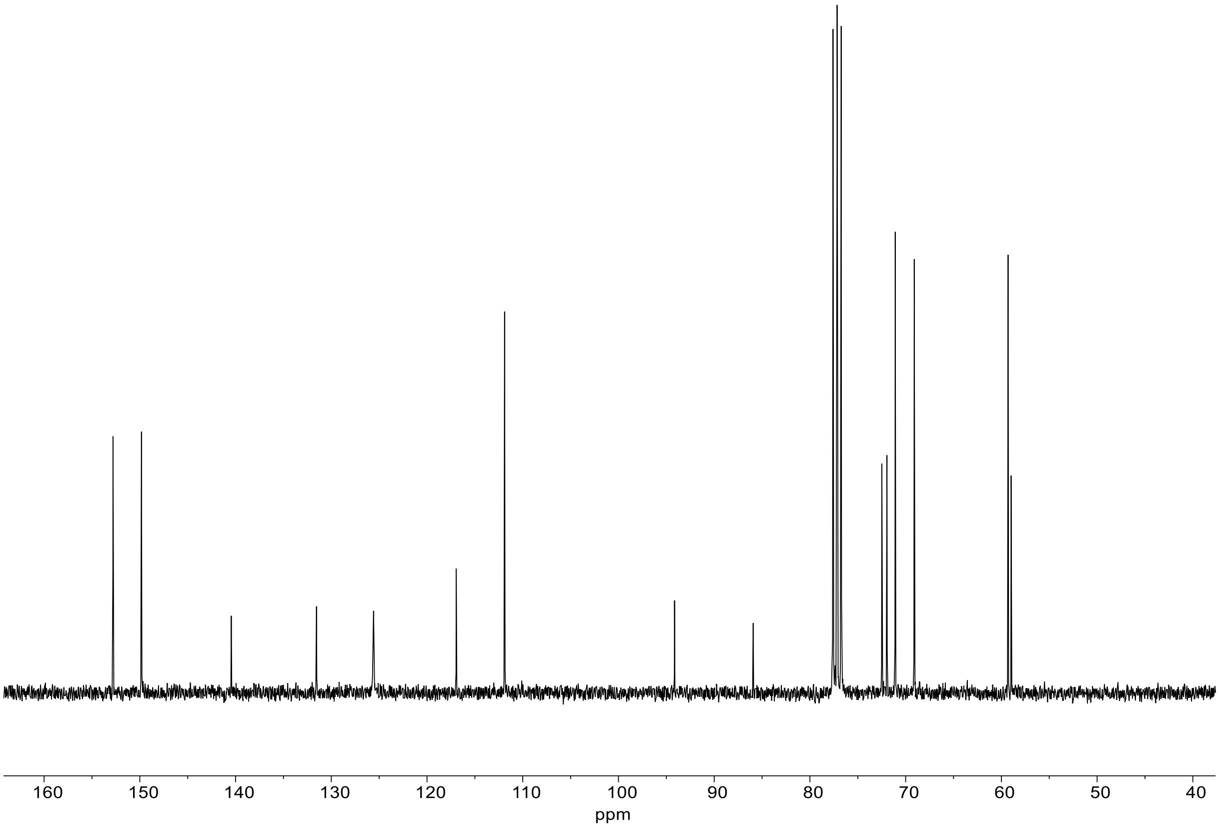

*^{13}C NMR of **21** in $CDCl_3$.*

*Synthesis of Ligand **24***:

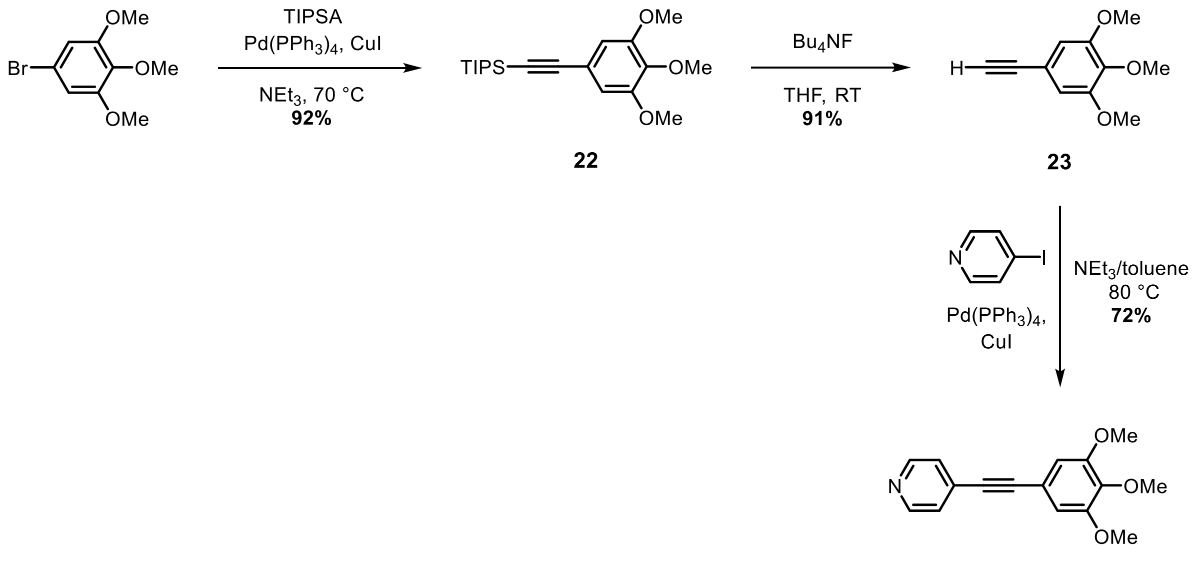

24

*Synthesis of triisopropyl((3,4,5-trimethoxyphenyl)ethynyl)silane (**22**):* 5-Bromo-1,2,3-trimethoxybenzene (1.50 g, 6.07 mmol), $Pd(PPh_3)_4$ (175 mg, 151 μmol) and CuI (28.9 mg, 151 μmol) were dissolved in degassed NEt_3 (20 mL). The mixture was subjected to five vacuum/argon cycles and heated to 80°C. While stirring the mixture, triisobutylsilylacetylene (2.21 g, 3.16 mL, 12.1 mmol) was added slowly to the reaction and the mixture was left to stir at 80 °C for 16 h. After cooling to room temperature, the solvent was removed under reduced pressure. The residue was purified by column chromatography (silica, pentane/DCM 80:20 to 50:50) affording **22** as yellow crystals (1.94 g, 5.57 mmol, 92 %).

MS-ESI: m/z calculated for $[C_{20}H_{32}O_3SiNa]^+$ $[M+Na^+]$: 371.2013; found 371.2012.

^{1}H NMR (300 MHz, $CDCl_3$, 298 K): δ (in ppm) 6.69 (s, 2H, phenyl-*H*), 3.86 (s, 6H, OCH_3), 3.84 (s, 3H, OCH_3), 1.13 (s, 21H, C*H*, CH_3).
^{13}C NMR (75 MHz, $CDCl_3$, 298 K): δ (in ppm) 153.1, 139.1, 118.7, 109.3, 107.1, 89.6, 61.1, 56.3, 18.8, 11.5.

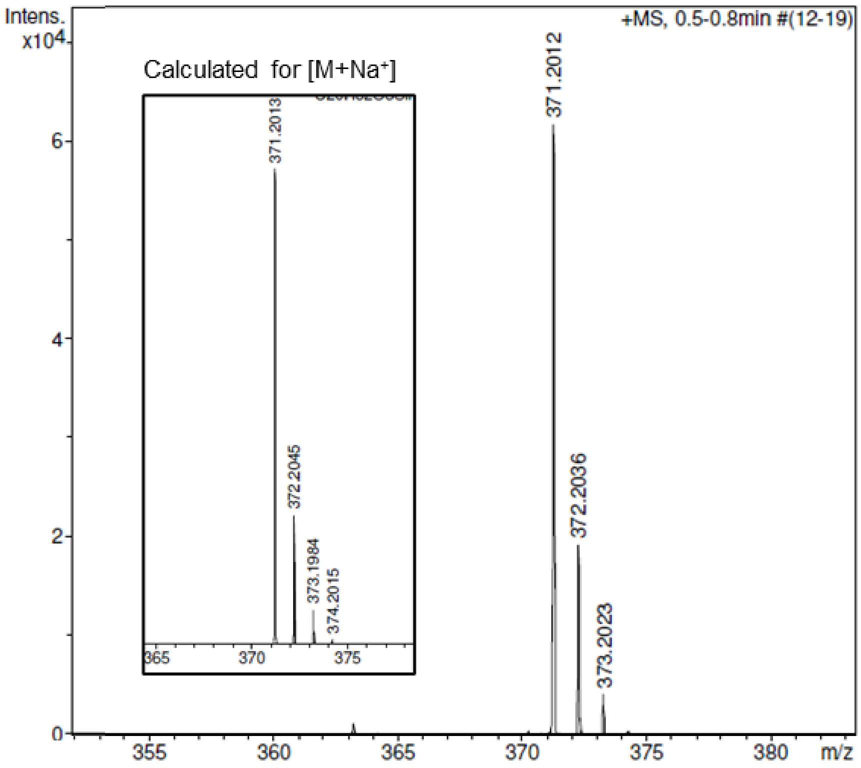

*MS-ESI of **22**.*

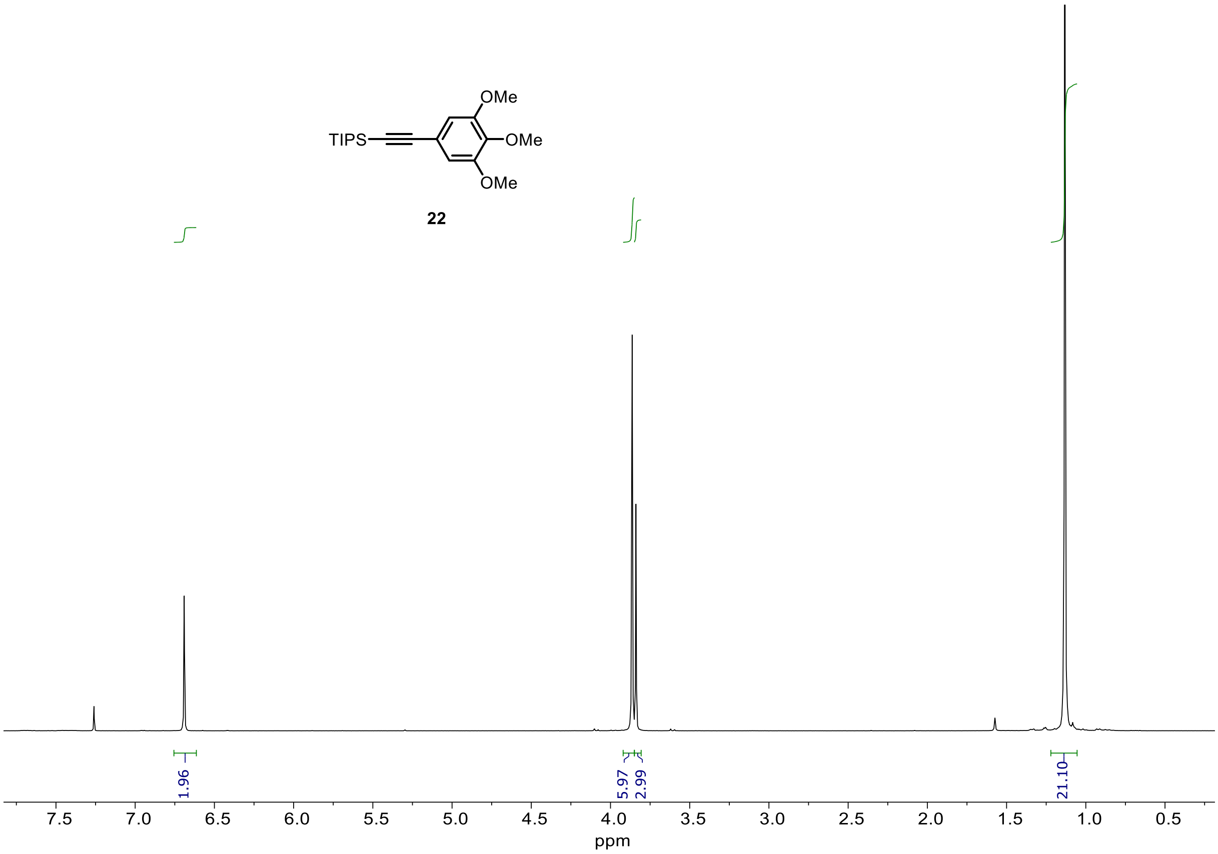

*^{1}H NMR of **22** in $CDCl_3$.*

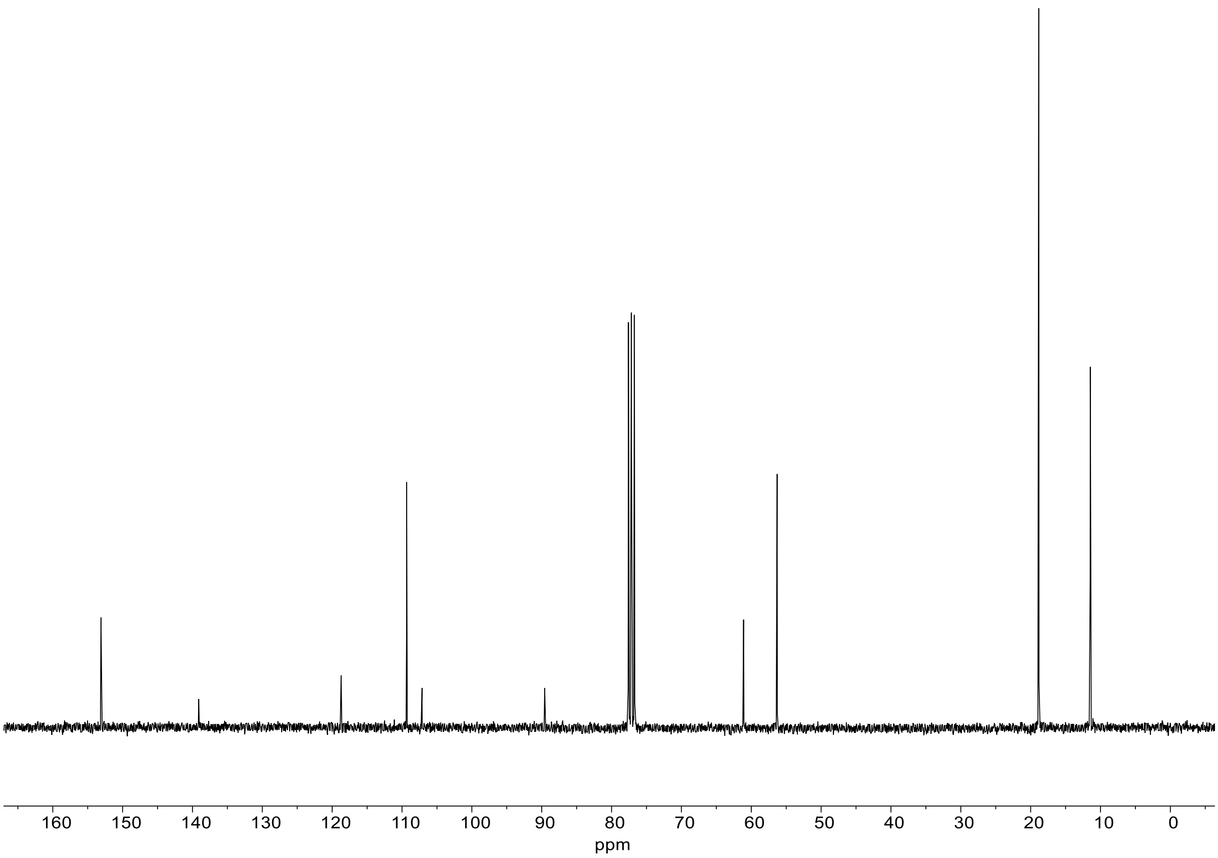

*^{13}C NMR of **22** in $CDCl_3$.*

*Synthesis of 5-ethynyl-1,2,3-trimethoxybenzene (**23**):* A tetrabutylammonium fluoride solution (1M in THF, 8.27 mL, 8.27 mmol) was added to a mixture of **22** (1.92 g, 5.51 mmol) in THF (50 mL) at room temperature. After stirring for 16 h the reaction mixture was concentrated under reduced pressure. DCM (80 mL) was added and the solution was washed with water (3 × 30 mL) and brine (30 mL). The organic layer was dried over Na_2SO_4 and the solvent was removed under reduced pressure. The residue was purified by column chromatography (silica, pentane/DCM 67:33 to 50:50) affording **23** as a yellow solid (968 mg, 5.04 mmol, 91%).

MS-ESI: m/z calculated for $[C_{11}H_{12}O_3Na]^+$ $[M+Na^+]$: 215.0679; found 215.0685.

^{1}H NMR (300 MHz, $CDCl_3$, 298 K): δ (in ppm) 6.73 (s, 2H, phenyl-*H*), 3.85 (s, 9H, OCH_3), 3.03 (s, 1H, CC*H*).
^{13}C NMR (75 MHz, $CDCl_3$, 298 K): δ (in ppm) 153.1, 139.3, 117.1, 109.4, 83.8, 76.4, 61.1, 56.3.

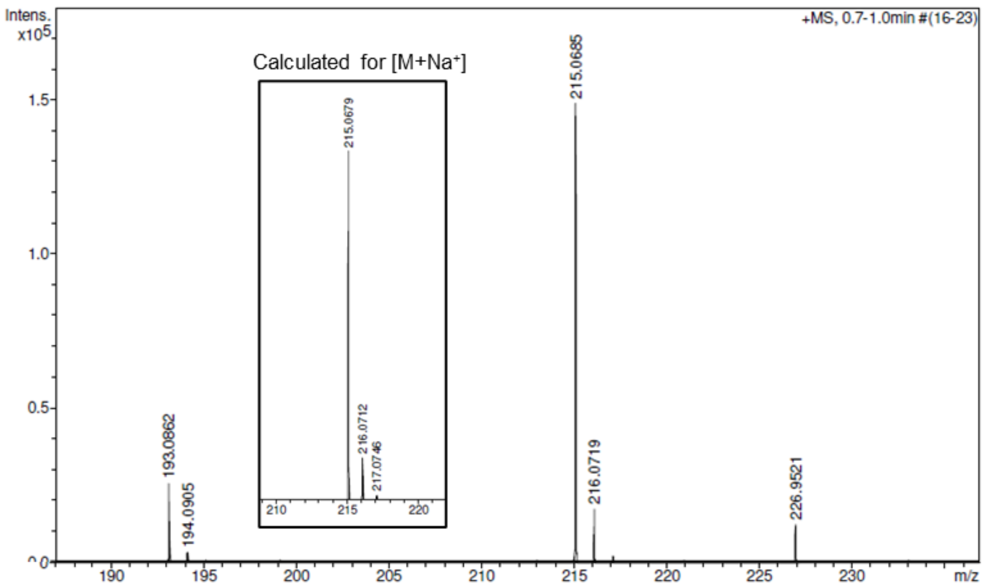

*MS-ESI of **23**.*

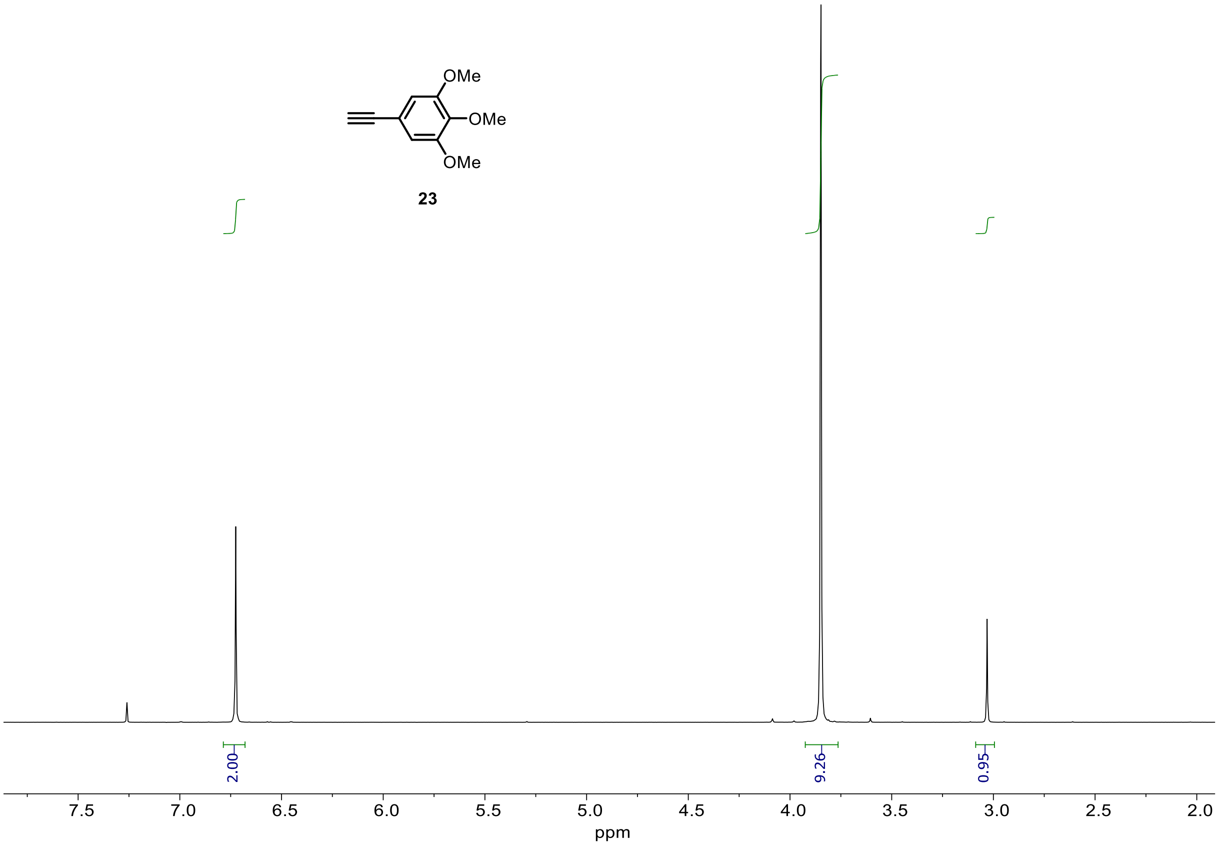

*^{1}H NMR of **23** in $CDCl_3$.*

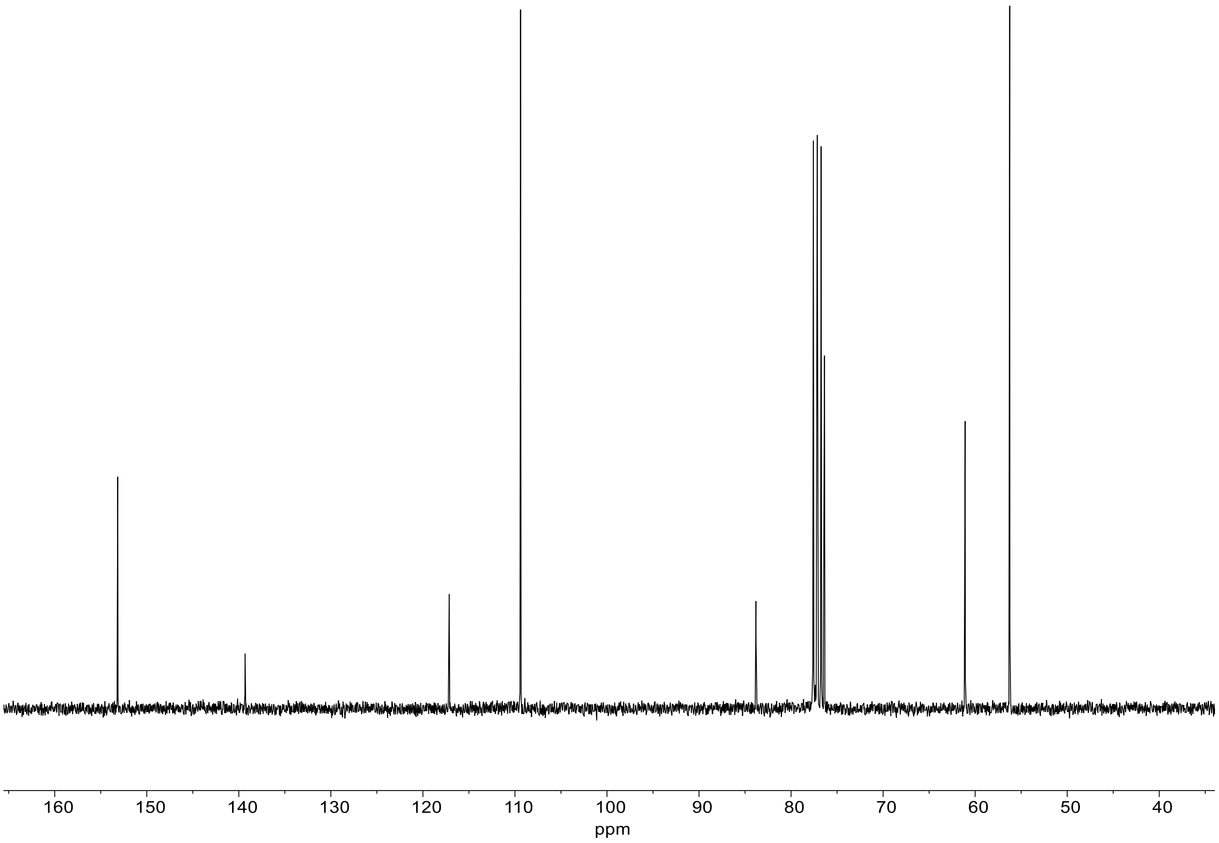

*^{13}C NMR of **23** in $CDCl_3$.*

*Synthesis of 4-((3,4,5-trimethoxyphenyl)ethynyl)pyridine (**24**):* 4-Iodopyridine (1.06 g, 5.17 mmol), $Pd(PPh_3)_4$ (114 mg, 98.4 *µ*mol) and CuI (18.8 mg, 98.4 *µ*mol) were placed in a Schlenk tube and degassed NEt_3 (20 mL) was added. The mixture was subjected to five vacuum/argon cycles. A solution of compound **23** (946 mg, 4.92 mmol) in degassed toluene (10 mL) was added slowly to the reaction mixture at 80 °C while stirring. The reaction was left to stir at 80 °C for 16 h. The solvent was removed, and the residue was purified by column chromatography (silica, pentane/DCM 80:20 to neat DCM) to yield **24** as pale yellow solid (957 mg, 3.55 mmol, 72%).

MS-ESI: m/z calculated for $[C_{16}H_{15}NO_3H]^+$ $[M+H^+]$: 270.1125; found 270.1128.

^{1}H NMR (300 MHz, $CDCl_3$, 298 K): *δ* (in ppm) 8.61 (unresolved m, 2H, pyridine-*H*), 7.38 (m, 2H, pyridine-*H*), 6.78 (s, 2H, phenyl-*H*), 3.88 (s, 6H, OCH_3), 3.88 (s, 3H, OCH_3).
^{13}C NMR (75 MHz, $CDCl_3$, 298 K): *δ* (in ppm) 153.3, 149.8, 139.7, 131.5, 125.7, 117.1, 109.2, 94.3, 85.9, 61.1, 56.3.

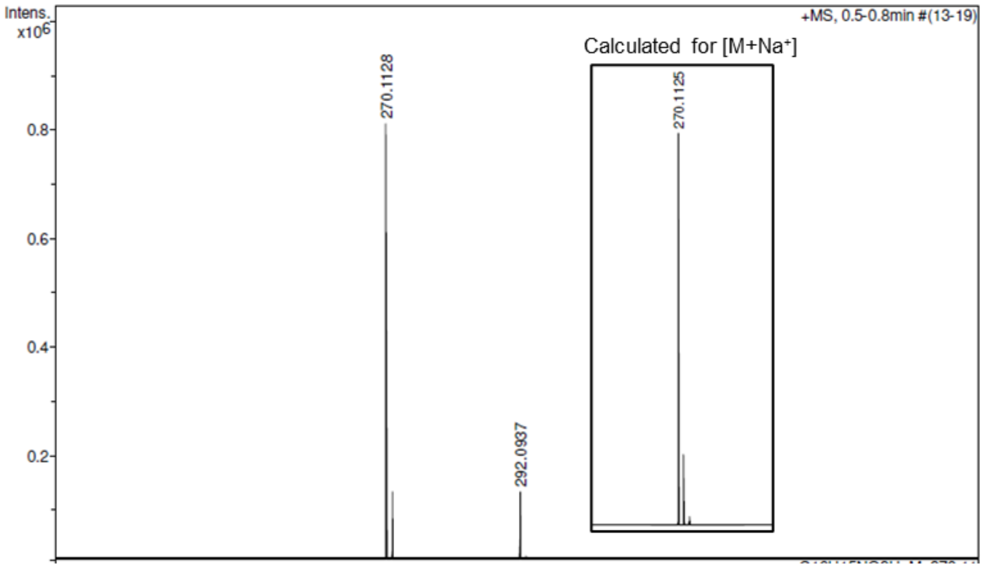

*MS-ESI of **24**.*

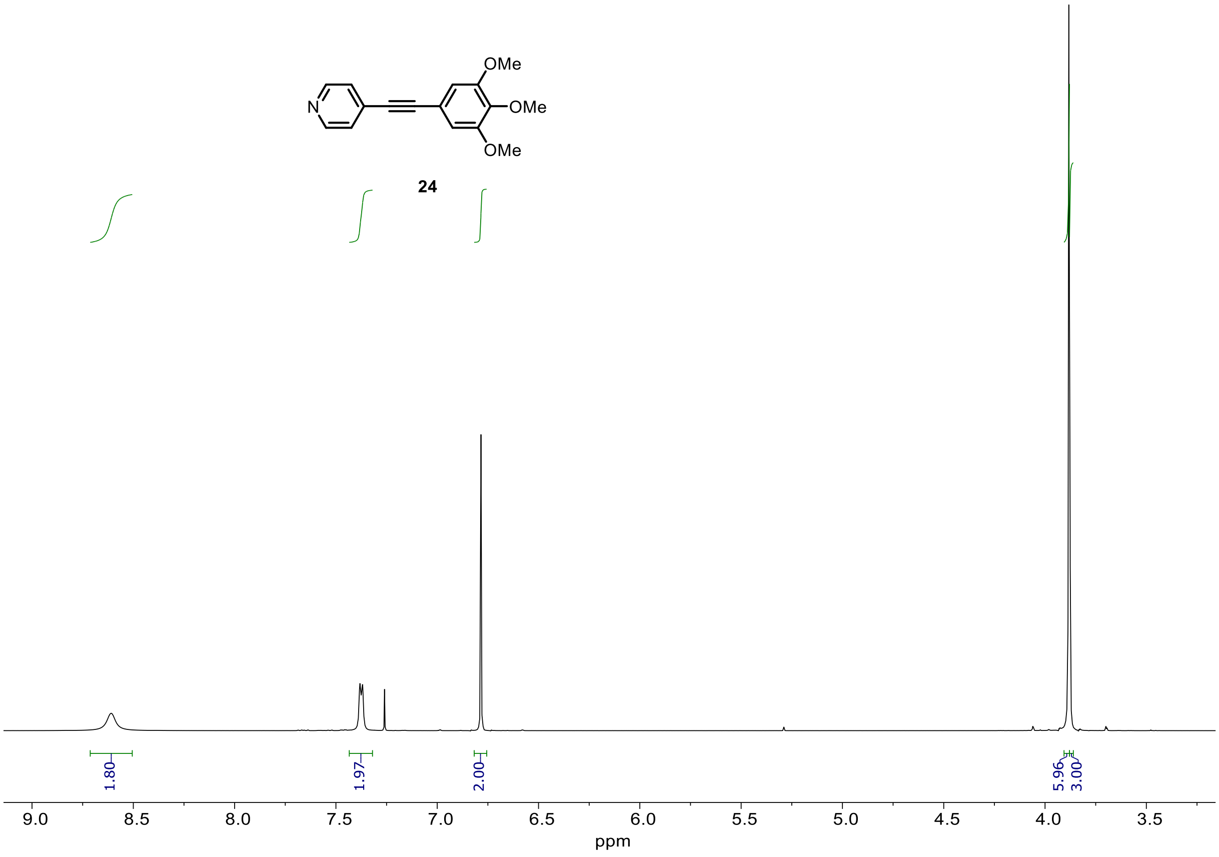

*^{1}H NMR of **24** in $CDCl_3$.*

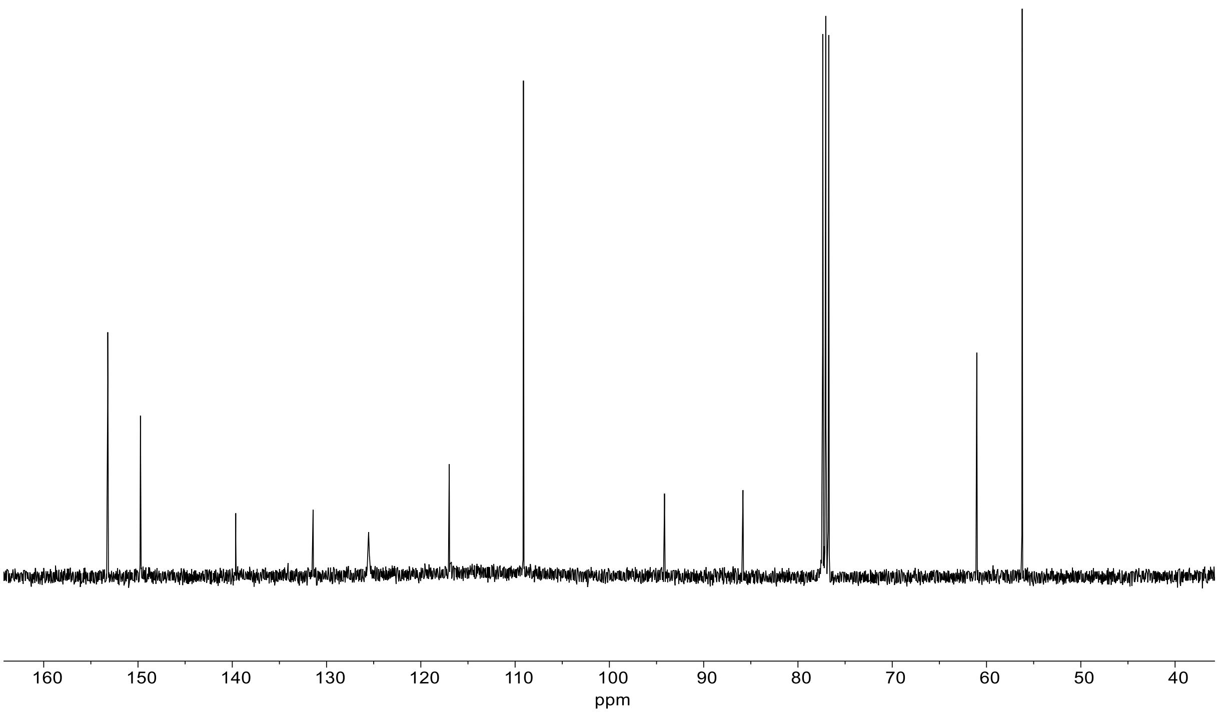

*^{13}C NMR of **24** in $CDCl_3$.*

*Synthesis of complexes **1**-**4***:

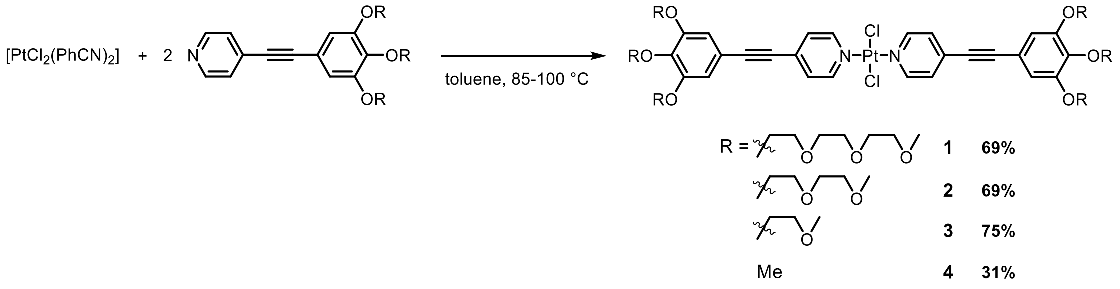

*Synthesis of **1**:* A mixture of $PtCl_2(PhCN)_2$ (42.9 mg, 90.9 *μ*mol) and **19** (110 mg, 165 *μ*mol) in degassed benzene (15 mL) was subjected to five vacuum/argon cycles and then stirred at 85 °C for 2 days. After evaporation of the solvent under reduced pressure, the residue was purified by column chromatography (alumina N act III, Et_2O/EtOAc/MeOH 74:24:2). For further purification, the resulting solid was diluted in DCM and precipitated with Et_2O. Product **1** was obtained as a yellow solid (91.7 mg, 57.4 *μ*mol, 69%).

Elemental analysis: calculated for $C_{68}H_{102}N_2O_{24}Cl_2Pt$: C 51.13, H 6.44, N 1.75; found: C 51.04, H 6.44, N 1.67.

^{1}H NMR (400 MHz, CD_2Cl_2, 298 K): δ (in ppm) 8.83 (m, 4H, pyridine-*H*), 7.38 (m, 4H, pyridine-*H*), 6.86 (s, 4H, phenyl-*H*), 4.20 – 4.14 (m, 12H, OCH_2), 3.87 – 3.82 (m, 8H, OCH_2), 3.78 – 3.74 (m, 4H, OCH_2), 3.72 – 3.55 (m, 36H, OCH_2), 3.52 – 3.46 (m, 12H, OCH_2), 3.33 (s, 6H, OCH_3), 3.33 (s, 12H, OCH_3).

^{13}C NMR (150.9 MHz, CD_2Cl_2, 298 K): δ (in ppm) 153.6, 153.2, 141.2, 134.8, 127.4, 116.4, 112.0, 99.0, 85.2, 73.1, 72.5, 71.4, 71.2, 71.1, 71.1, 71.0, 71.0, 70.1, 69.5, 59.2.

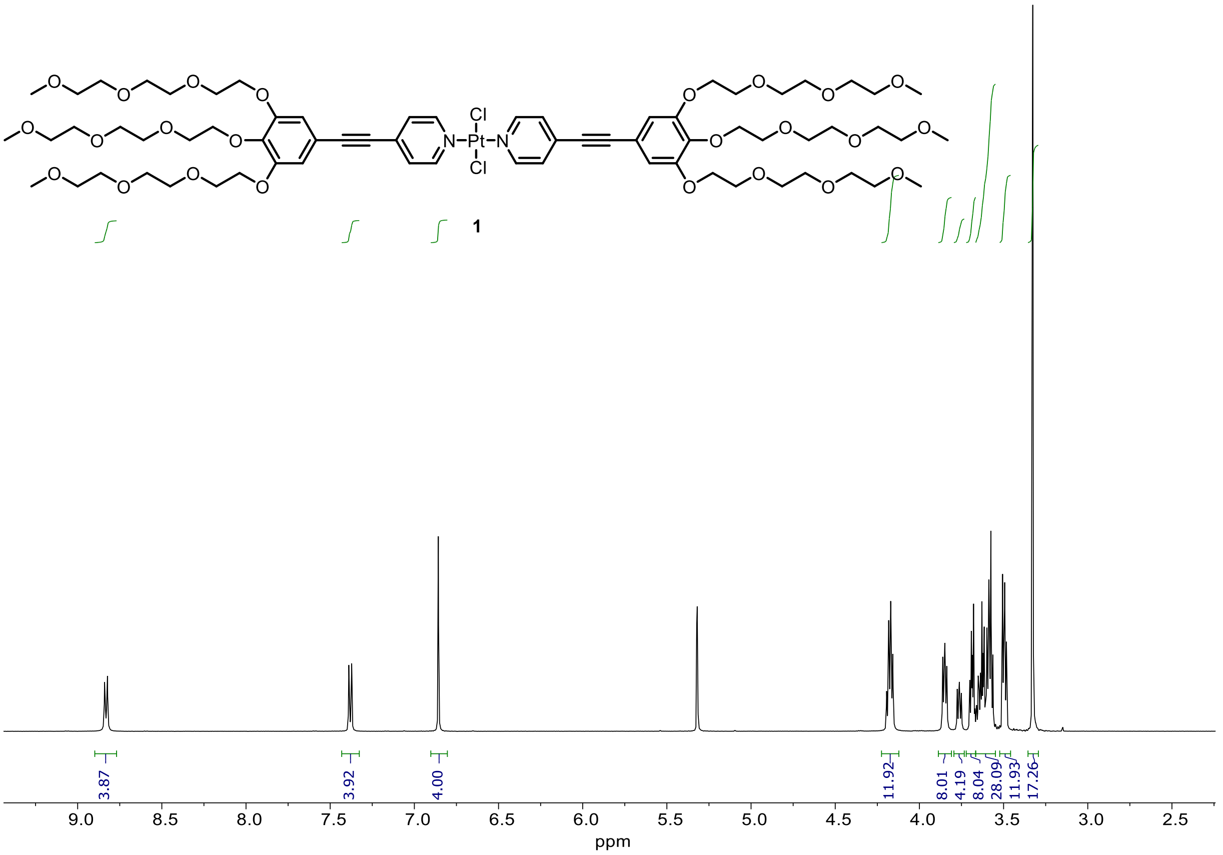

*^{1}H NMR of **1** in CD_2Cl_2.*

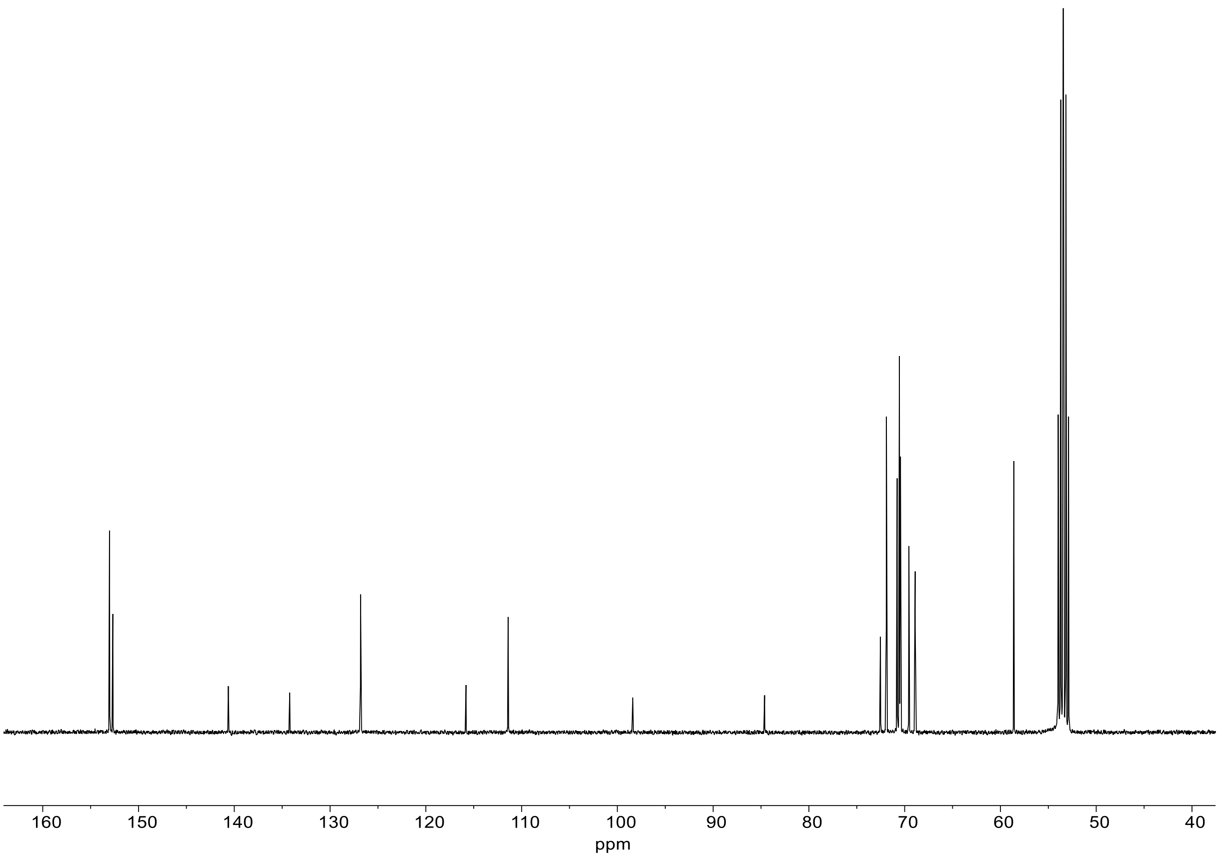

*^{13}C NMR of **1** in CD_2Cl_2.*

*Synthesis of **2**:* A mixture of $PtCl_2(PhCN)_2$ (69.7 mg, 148 μmol) and **20** (150 mg, 281 μmol) in degassed toluene (15 mL) was subjected to five vacuum/argon cycles. The reaction was then left to stir at 100 °C for 3 days. After evaporation of the solvent under reduced pressure, the residue was purified by column chromatography (silica, Et_2O/EtOAc/MeOH 49:49:2). For further purification, the resulting solid was diluted in DCM and precipitated with Et_2O. Product **2** was obtained as a yellow solid (130.0 mg, 97.5 μmol, 69%).

Elemental analysis: calculated for $C_{56}H_{78}N_2Cl_2O_{18}Pt$: C 50.45, H 5.90, N 2.10; found: C 50.38, H 5.86, N 1.81.

^{1}H NMR (300 MHz, CD_2Cl_2, 298 K): δ (in ppm) 8.83 (m, 4H, pyridine-*H*), 7.38 (m, 4H, pyridine-*H*), 6.85 (s, 4H, phenyl-*H*), 4.22 – 4.11 (m, 12H, OCH_2), 3.88 – 3.80 (m, 8H, OCH_2), 3.79 – 3.72 (m, 4H, OCH_2), 3.71 – 3.60 (m, 12H, OCH_2), 3.56 – 3.48 (m, 12H, OCH_2), 3.34 (s, 12H, OCH_3), 3.33 (s, 6H, OCH_3).

^{13}C NMR (75 MHz, CD_2Cl_2, 298 K): δ (in ppm) 153.4, 153.0, 140.9, 134.6, 127.2, 116.2, 111.6, 98.7, 85.0, 72.9, 72.3, 72.3, 71.0, 70.9, 70.7, 69.9, 69.2, 59.1, 59.0.

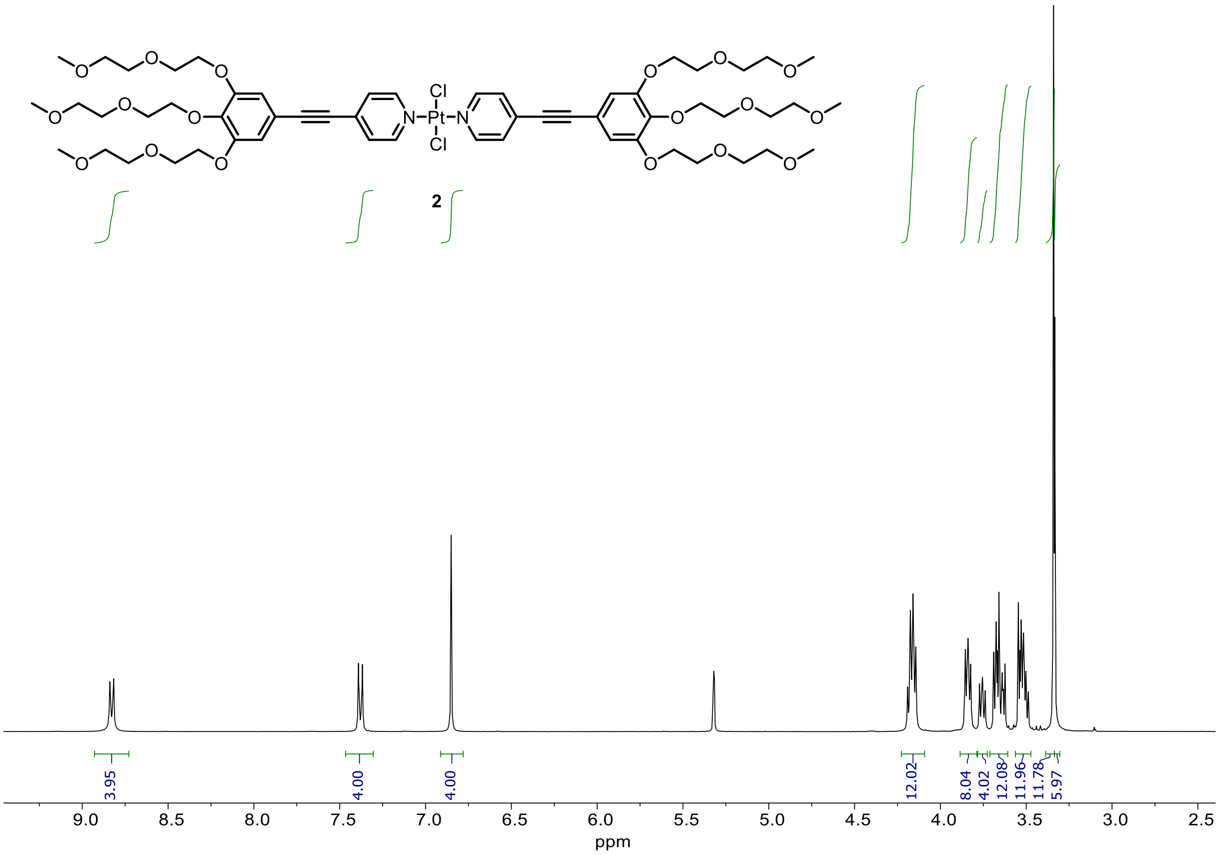

*^{1}H NMR of **2** in CD_2Cl_2.*

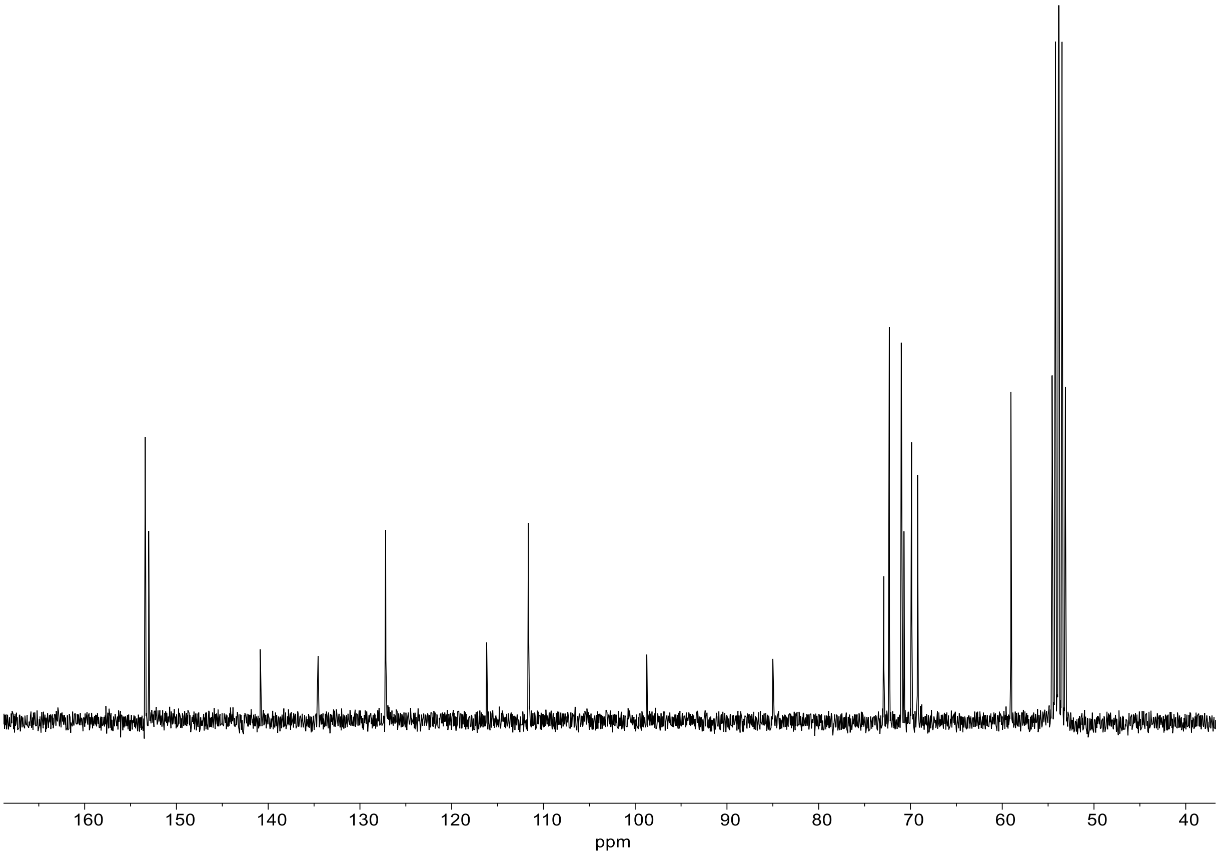

*^{13}C NMR of **2** in CD_2Cl_2.*

*Synthesis of **3**:* A mixture of $PtCl_2(PhCN)_2$ (61.8 mg, 131 μmol) and **21** (100 mg, 249 μmol) in degassed toluene (10 mL) was subjected to five vacuum/argon cycles. The reaction was then left to stir at 100 °C for 3 days. After evaporation of the solvent under reduced pressure the residue was purified by column chromatography (silica, DCM/Et_2O/MeOH 70:29:1). For further purification, the resulting solid was diluted in DCM and precipitated with Et_2O. Product **3** was obtained as yellow solid (99.3 mg, 92.9 μmol, 69%).

Elemental analysis: calculated for $C_{44}H_{54}N_2Cl_2O_{12}Pt$: C 49.44, H 5.09, N 2.62; found: C 49.37, H 4.99, N 2.47.

^{1}H NMR (400 MHz, CD_2Cl_2, 298 K): δ (in ppm) 8.83 (m, 4 H, pyridine-*H*), 7.38 (m, 4 H, pyridine-*H*), 6.84 (s, 4 H, phenyl-*H*), 4.17 – 4.12 (m, 12 H, OCH_2), 3.78 – 3.73 (m, 8 H, OCH_2), 3.69 – 3.65 (m, 4 H, OCH_2), 3.42 (s, 12 H, OCH_3), 3.39 (s, 6 H, OCH_3).
^{13}C NMR (101 MHz, CD_2Cl_2, 298 K): δ (in ppm) 153.4, 153.1, 140.9, 134.6, 127.2, 116.3, 111.6, 98.7, 85.0, 72.8, 72.3, 71.3, 69.1, 59.2, 58.9.

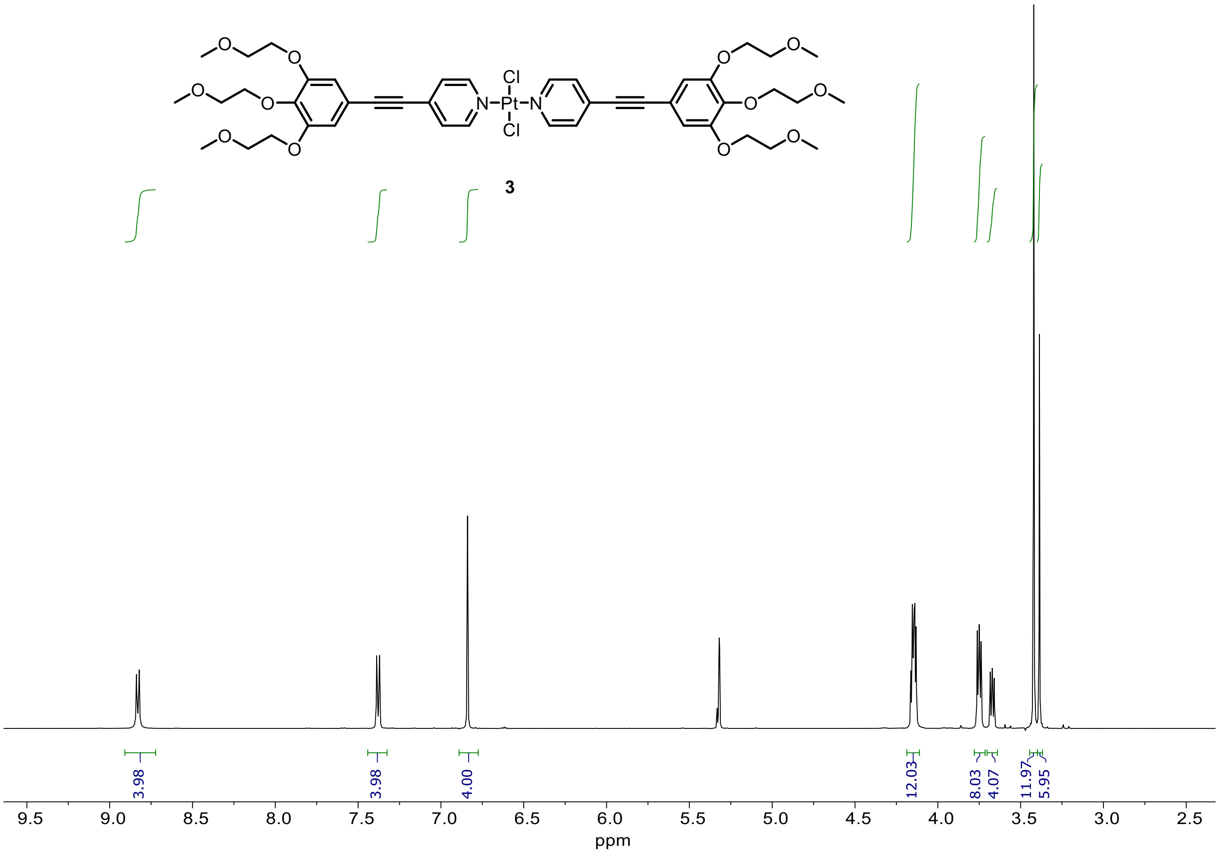

*^{1}H NMR of **3** in CD_2Cl_2.*

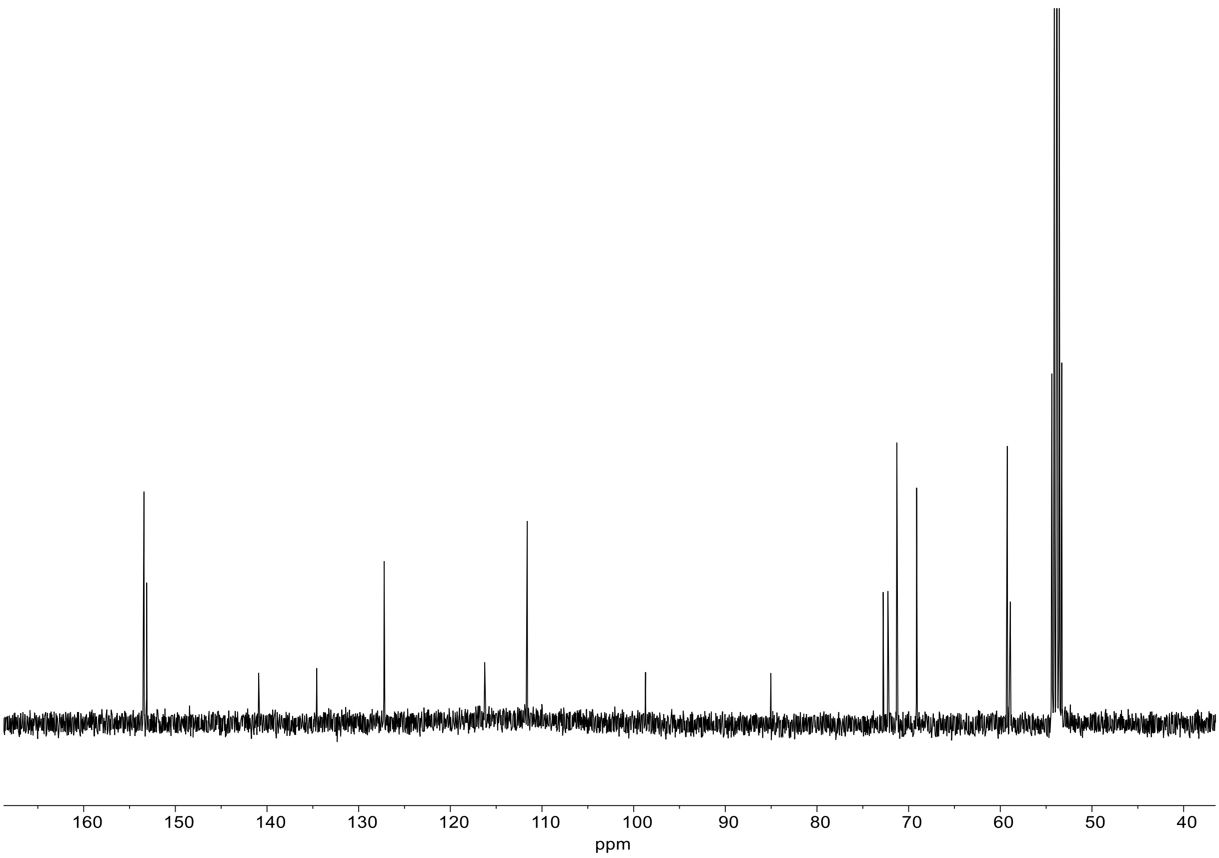

*^{13}C NMR of **3** in CD_2Cl_2.*

*Synthesis of **4**:* A mixture of $PtCl_2(PhCN)_2$ (92.1 mg, 195 μmol) and **24** (100 mg, 371 μmol) were dissolved in degassed toluene (15 mL). The mixture was subjected to five vacuum/argon cycles and afterwards stirred at 100 °C for 5 days. After evaporation of the solvent under reduced pressure, the residue was purified by column chromatography (silica, neat DCM to DCM/MeOH 98:2). For further purification, the resulting solid was diluted in DCM and precipitated with Et_2O. Product **4** was obtained as yellow solid (46.8 mg, 58.2 μmol, 31%).

Elemental analysis: calculated for $C_{32}H_{30}N_2Cl_2O_6Pt$: C 47.77, H 3.76, N 3.48; found: C 47.61, H 3.74, N 3.18.

MS-ESI: m/z calculated for $[C_{32}H_{30}Cl_2N_2O_6PtNa]^+$ $[M+Na^+]$: 826.10264; found: 826.10310.

MALDI-TOF (DCTB): m/z calculated for $[C_{32}H_{30}Cl_2N_2O_6Pt]^+$: 804.11; found 804.00.

^1H NMR (300 MHz, CD_2Cl_2, 298 K): δ (in ppm) 8.84 (m, 4H, pyridine-*H*), 7.39 (m, 4H, pyridine-*H*), 6.84 (s, 4H, phenyl-*H*), 3.87 (s, 12H, OCH_3), 3.82 (s, 6H, OCH_3).

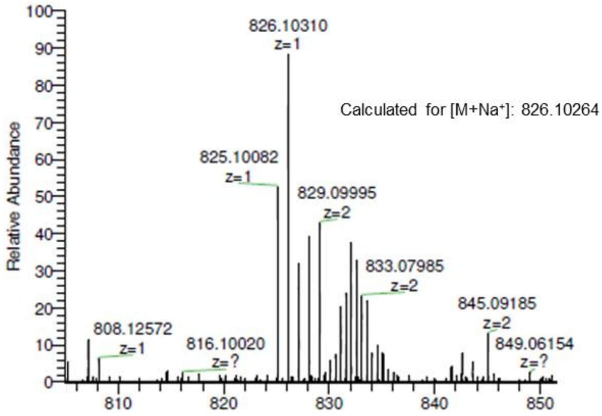

*MS-ESI of **4**.*

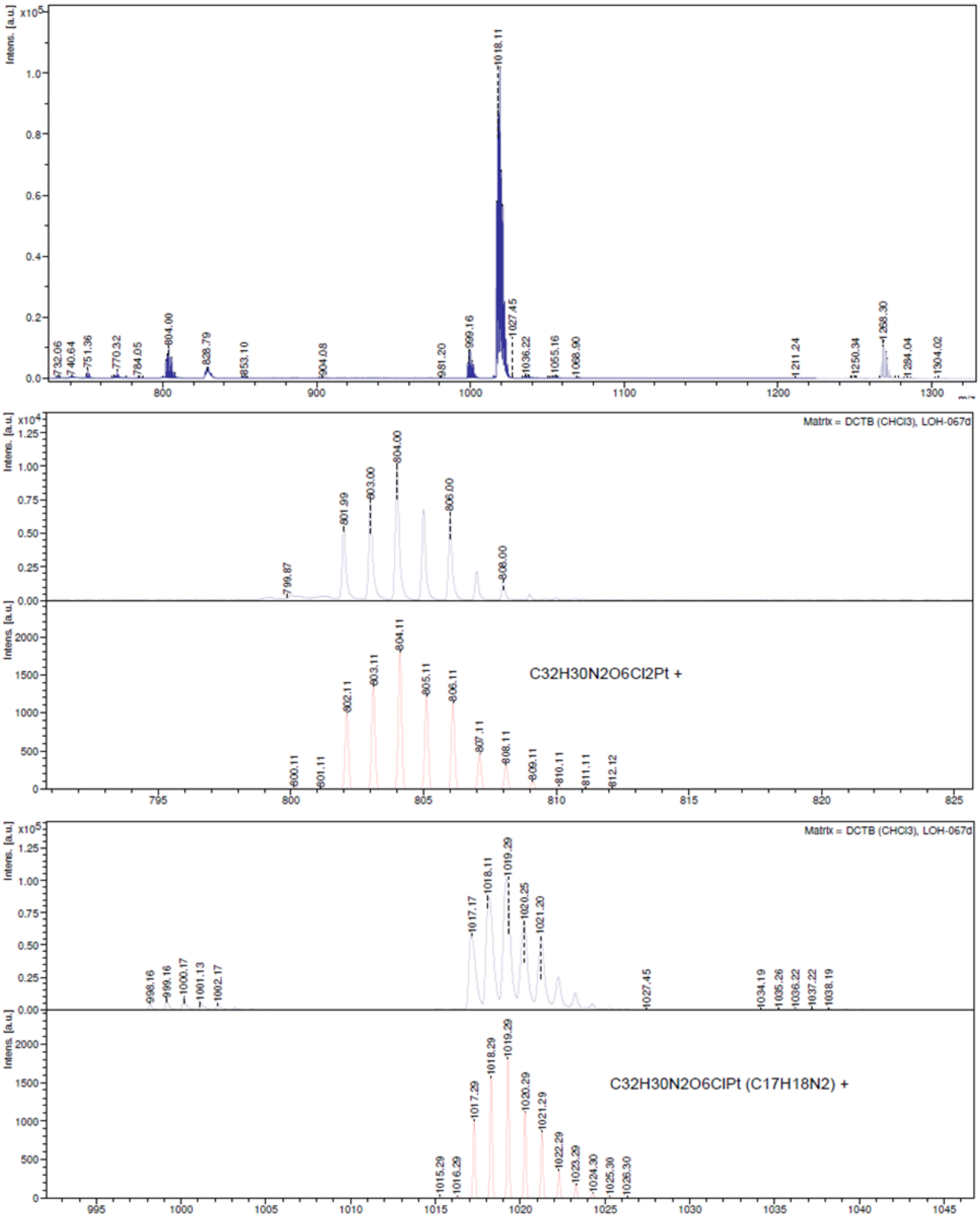

*MALDI-TOF of **4**.*

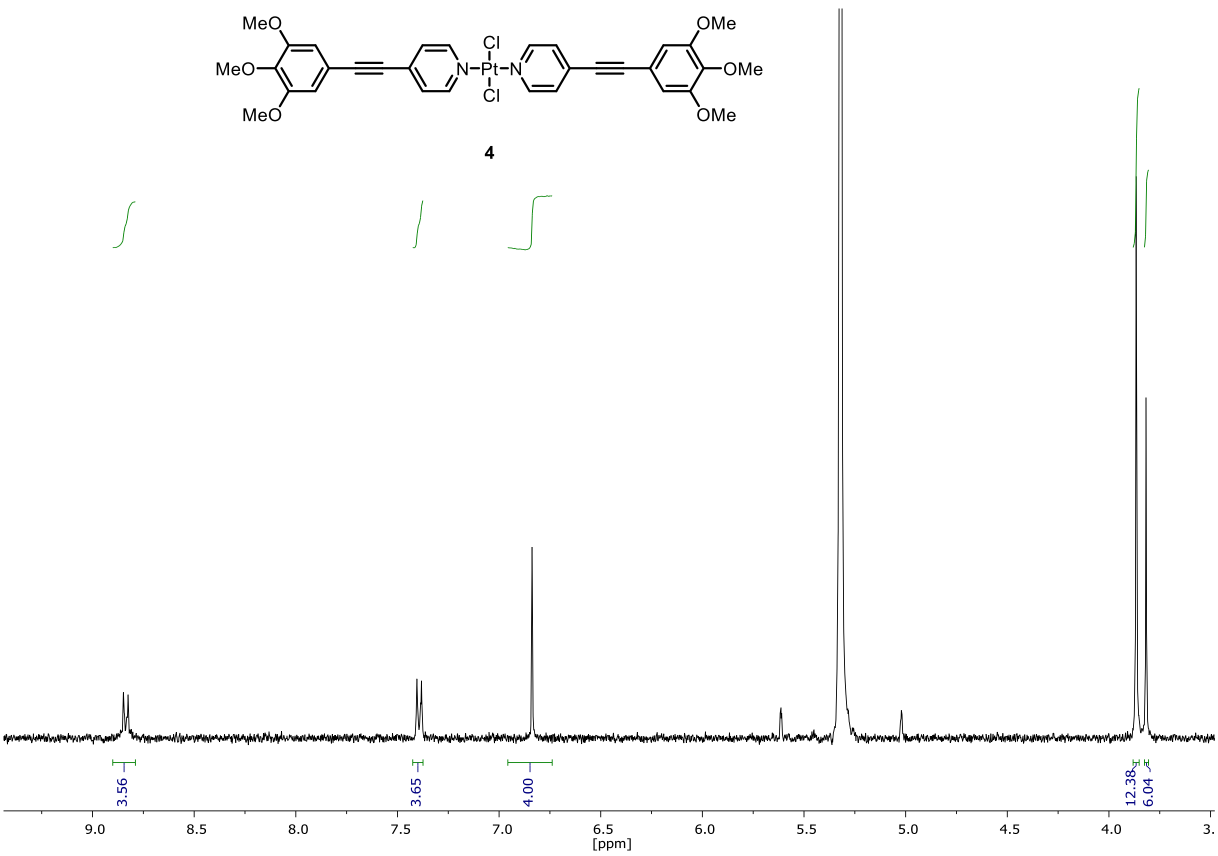

*^{1}H NMR of **4** in CD_2Cl_2.*

B. Crystal Structure Analysis

***X-Ray diffraction*:** Data sets for compounds **1** and **2** data sets were collected with a Bruker APEX II CCD diffractometer. Programs used: data collection: APEX3 V2016.1-0 [6]; cell refinement: SAINT V8.37A[7]; data reduction: SAINT V8.37A[7]; absorption correction, SADABS V2014/7[8]; structure solution *SHELXT-2015*[9]; structure refinement *SHELXL-2015*[10]. For compound **3**, data sets were collected with a Nonius Kappa CCD diffractometer. Programs used: data collection, COLLECT[11]; data reduction Denzo-SMN[12]; absorption correction, Denzo[13]; structure solution *SHELXT-2015*[9]; structure refinement *SHELXL-2015*[10] and graphics, *XP*[14]. *R*-values are given for observed reflections, and wR^2 values are given for all reflections.

Exceptions and special features: For compound **3**, all ethylene glycol groups are disordered over two positions. Several restraints (SADI, SAME, ISOR and SIMU) were used in order to improve refinement stability.

***X-ray crystal structure analysis of 1*:** A colorless plate-like specimen of $C_{68}H_{102}Cl_2N_2O_{24}Pt$, approximate dimensions 0.040 mm x 0.160 mm x 0.200 mm, was used for the X-ray crystallographic analysis. The X-ray intensity data were measured. The integration of the data using a triclinic unit cell yielded a total of 31238 reflections to a maximum θ angle of 68.11° (0.83 Å resolution), of which 6521 were independent (average redundancy 4.790, completeness = 97.6%, R_{int} = 3.64%, R_{sig} = 2.73%) and 6497 (99.63%) were greater than $2\sigma(F^2)$. The final cell constants of a = 9.8543(2) Å, b = 12.7135(3) Å, c = 15.7043(3) Å, α = 101.5470(10)°, β = 103.6920(10)°, γ = 98.9700(10)°, volume = 1829.67(7) $Å^3$, are based upon the refinement of the XYZ-centroids of reflections above 20 σ(I). Data were corrected for absorption effects using the multi-scan method (SADABS). The calculated minimum and maximum transmission coefficients (based on crystal size) are 0.4410 and 0.8280. The structure was solved and refined using the Bruker SHELXTL Software Package, using the space group *P*-1, with Z = 1 for the formula unit, $C_{68}H_{102}Cl_2N_2O_{24}Pt$. The final anisotropic full-matrix least-squares refinement on F^2 with 442 variables converged at R1 = 2.57%, for the observed data and wR2 = 6.52% for all data. The goodness-of-fit was 1.056. The largest peak in the final difference electron density synthesis was 1.388 $e^-/Å^3$ and the largest hole was -0.695 $e^-/Å^3$ with an RMS deviation of 0.090 $e^-/Å^3$. On the basis of the final model, the calculated density was 1.450 g/cm^3 and F(000), 828 e^-. CCDC Nr.: 2004413.

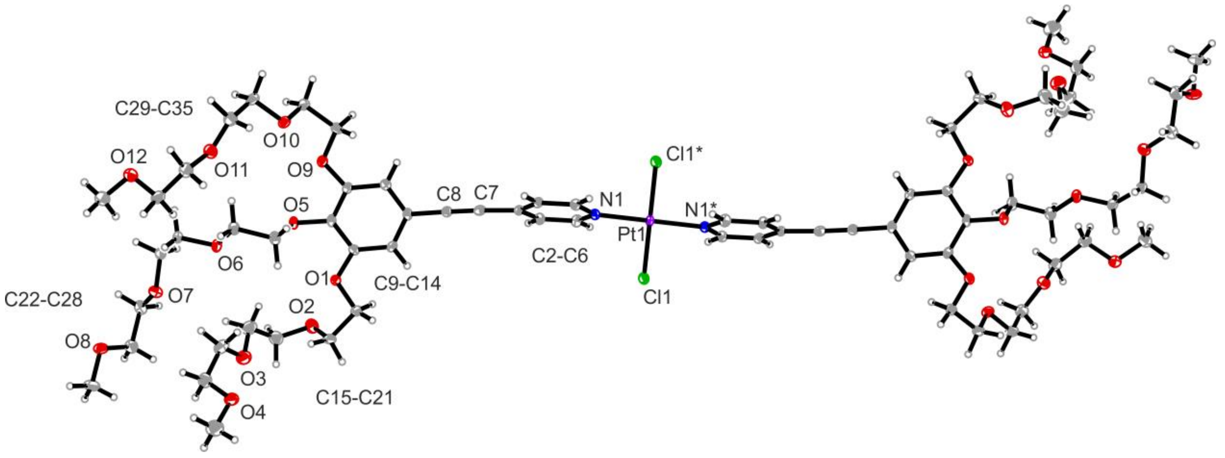

*Figure S1. Crystal structure of compound **1**. (Thermals ellipsoids are shown with 50% probability.)*

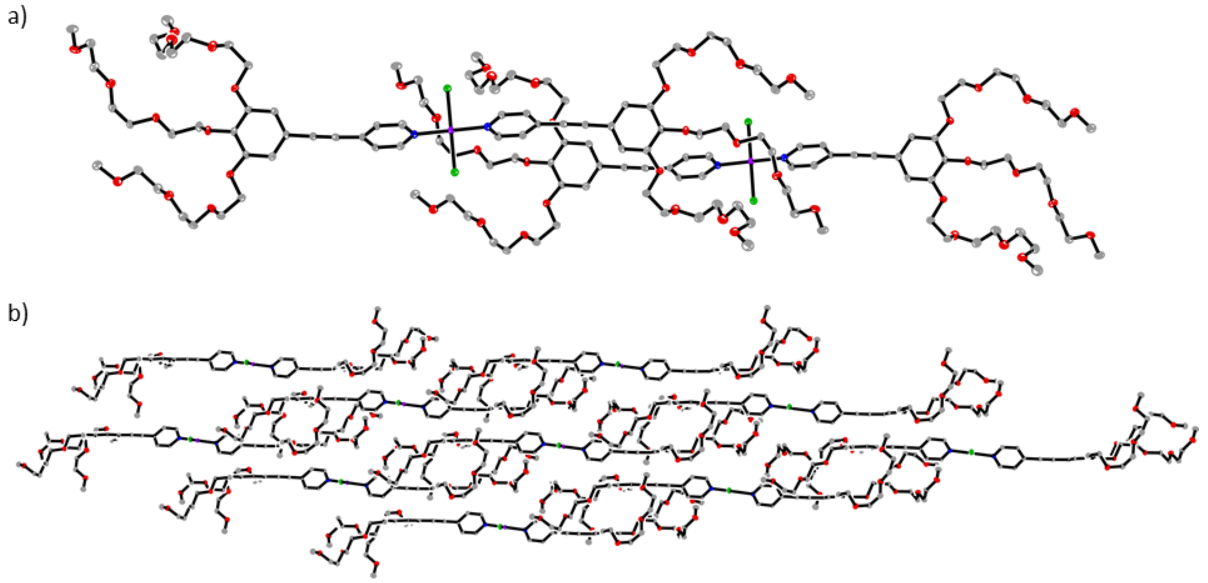

*Figure S2. a) Excerpt of the packing diagram of compound **1** presenting: a) top view of two neighboring molecules illustrating the positioning of the π-systems; b) overview of the 3D network, H atoms were omitted for clarity.*

Table S1. Non-covalent intermolecular interactions in compound **1** (Å and deg)

D-H···*A*	*d*(*D*-H)	*d*(H···*A*)	*d*(*D*···*A*)	∠(*DHA*)
C3-H3···O1[#1]	0.95	2.58	3.287(3)	131.4
C3-H3···O5[#1]	0.95	2.31	3.187(3)	153.0
C5-H5···O7[#2]	0.95	2.56	3.321(3)	136.9
C6-H6···O8[#2]	0.95	2.43	3.161(3)	133.8
C14-H14···O4[#2]	0.95	2.41	3.336(3)	165.7
C25-H25A···O8[#3]	0.99	2.55	3.450(3)	150.8
C25-H25B···Pt1[#4]	0.99	2.91	3.810(3)	151.9
C33-H33A···O12[#5]	0.99	2.62	3.609(4)	172.8
Pt1···Pt1[#6] (shortest)			9.854	

Symmetry transformations used to generate equivalent atoms: [#1] -x, -y+1, -z+1; [#2] -x+1, -y+1, -z+1; [#3] -x+1, -y, -z; [#4] x, y-1, z-1; [#5] -x+2, -y, -z+1; [#6] x+1, y, z.

***X-ray crystal structure analysis of 2*:** A pale yellow plate-like specimen of $C_{56}H_{78}Cl_2N_2O_{18}Pt$, approximate dimensions 0.020 mm x 0.080 mm x 0.140 mm, was used for the X-ray crystallographic analysis. The X-ray intensity data were measured. The integration of the data using a triclinic unit cell yielded a total of 20160 reflections to a maximum θ angle of 66.72° (0.84 Å resolution), of which 5121 were independent (average redundancy 3.937, completeness = 99.1%, R_{int} = 8.74%, R_{sig} = 7.85%) and 4704 (91.86%) were greater than 2σ(F^2). The final cell constants of a = 8.1261(3) Å, b = 9.7186(4) Å, c = 18.5258(8) Å, α = 88.278(3)°, β = 84.834(3)°, γ = 87.432(3)°, volume = 1455.16(10) $Å^3$, are based upon the refinement of the XYZ-centroids of reflections above 20 σ(I). Data were corrected for absorption effects using the multi-scan method (SADABS). The calculated minimum and maximum transmission coefficients (based on crystal size) are 0.4890 and 0.8900. The structure was solved and refined using the Bruker SHELXTL Software Package, using the space group *P*-1, with Z = 1 for the formula unit, $C_{56}H_{78}Cl_2N_2O_{18}Pt$. The final anisotropic full-matrix least-squares refinement on F^2 with 361 variables converged at R1 = 4.88%, for the observed data and wR2 = 11.39% for all data. The goodness-of-fit was 1.117. The largest peak in the final difference electron density synthesis was 1.072 $e^-/Å^3$ and the largest hole was -1.491 $e^-/Å^3$ with an RMS deviation of 0.155 $e^-/Å^3$. On the basis of the final model, the calculated density was 1.521 g/cm^3 and F(000), 684 e^-. CCDC Nr.: 2004414.

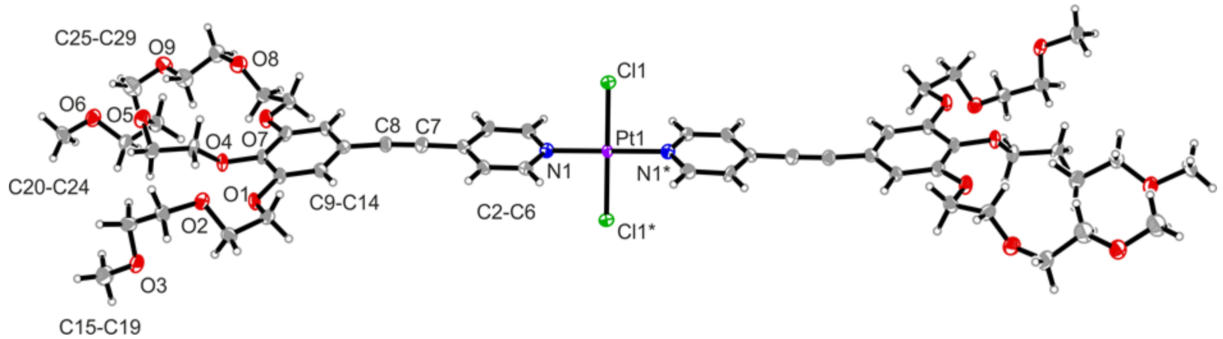

*Figure S3. Crystal structure of compound **2**. (Thermals ellipsoids are shown with 50% probability.)*

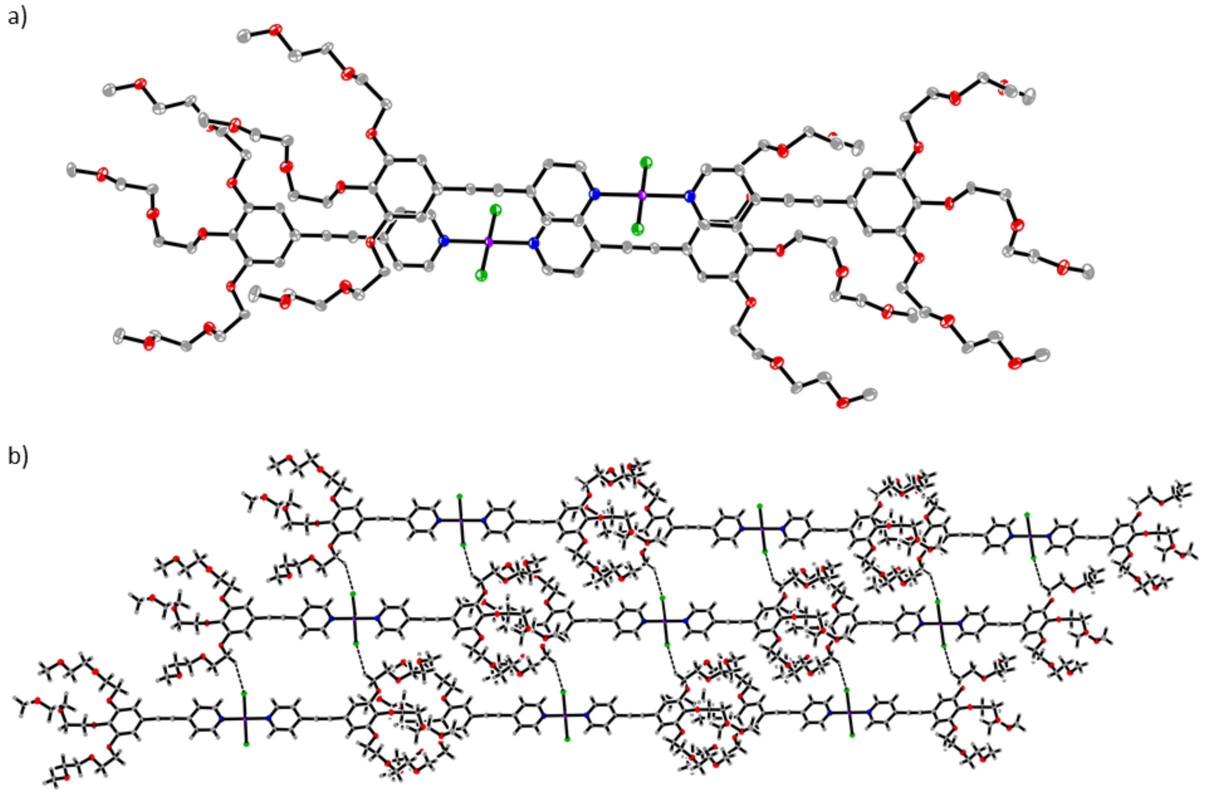

*Figure S4. Excerpt of the packing diagram of compound **2** presenting a) top view of two neighboring molecules illustrating the positioning of the π-systems; b) CH···Cl interactions between the dietylene glycol groups and adjacent Cl atoms.*

Table S2. Non-covalent intermolecular interactions in compound **2** (Å and deg).

D-H···*A*	*d*(*D*-H)	*d*(H···*A*)	*d*(*D*···*A*)	∠(*D*H*A*)
C5-H5···O7[#1]	0.95	2.45	3.352(7)	157.5
C15-H15B···Cl1[#2]	0.99	2.84	3.648(6)	139.8
C22-H22B···O8[#3]	0.99	2.59	3.493(8)	151.6
C25-H25A···O5[#4]	0.99	2.44	3.420(8)	171.4
C15-H15A···C3[#5]	0.99	2.90	3.725(9)	141.4
C24-H24A···*C*g2[#7]	0.99	2.73	3.529	138.2
*C*g1···*C*g1[#6]			3.208	
Pt1···Pt1[#5]			8.126	

Symmetry transformations used to generate equivalent atoms: [#1] -x+1, -y, -z+1; [#2] x+1, y+1, z; [#3] -x+2, -y, -z+1; [#4] x-1, y, z; [#5] x+1, y, z; [#6] -x, -y, -z+2; [#7] –x+2, -y+1, -z+1. *C*g1 is the centroid involving part of the pyridine ring containing C2 and C3 atoms; *C*g2 is the centroid involving part of the phenyl ring containing C12, C13 and O4 atoms.

X-ray crystal structure analysis of 3**:** A colorless plate-like specimen of $C_{44}H_{54}Cl_2N_2O_{12}Pt$, approximate dimensions 0.020 mm x 0.050 mm x 0.300 mm, was used for the X-ray crystallographic analysis. The X-ray intensity data were measured. The integration of the data using a monoclinic unit cell yielded a total of 4620 reflections to a maximum θ angle of 26.36° (0.80 Å resolution), of which 4620 were independent (average redundancy 1.000, completeness = 99.4%, R_{sig} = 2.55%) and 3832 (82.94%) were greater than 2σ(F^2). The final cell constants of a = 7.3395(2) Å, b = 10.1884(4) Å, c = 30.3908(8) Å, β = 92.2430(10)°, volume = 2270.81(12) $Å^3$, are based upon the refinement of the XYZ-centroids of reflections above 20 σ(I). Data were corrected for absorption effects using the multi-scan method (SADABS). The calculated minimum and maximum transmission coefficients (based on crystal size) are 0.4400 and 0.9370. The structure was solved and refined using the Bruker SHELXTL Software Package, using the space group $P2_1/n$, with Z = 2 for the formula unit, $C_{44}H_{54}Cl_2N_2O_{12}Pt$. The final anisotropic full-matrix least-squares refinement on F^2 with 394 variables converged at R1 = 3.09%, for the observed data and wR2 = 6.53% for all data. The goodness-of-fit was 1.059. The largest peak in the final difference electron density synthesis was 0.553 $e^-/Å^3$ and the largest hole was -0.645 $e^-/Å^3$ with an RMS deviation of 0.084 $e^-/Å^3$. On the basis of the final model, the calculated density was 1.563 g/cm^3 and F(000), 1080 e^-. CCDC Nr.: 2004415.

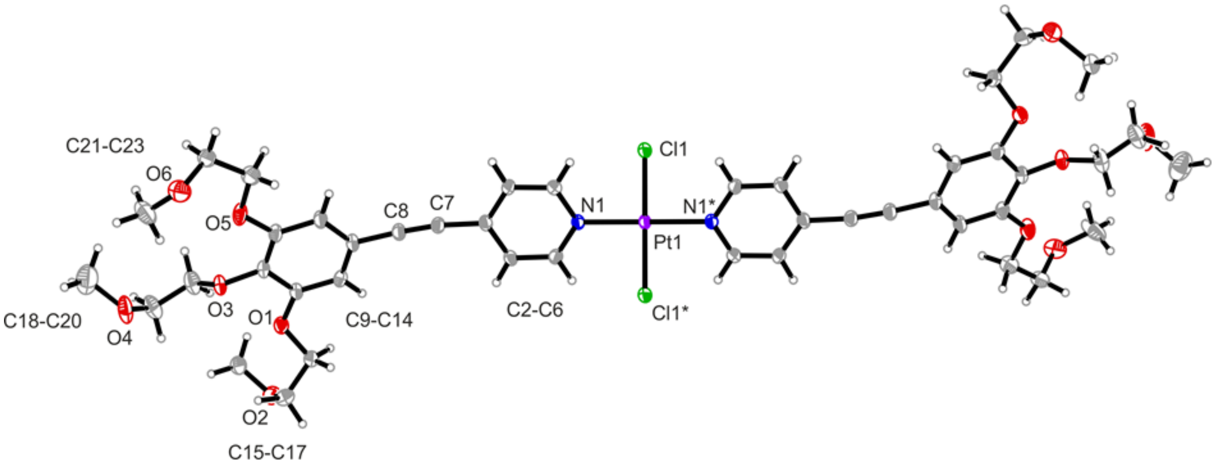

Figure S5. Crystal structure of compound **3**. (Thermals ellipsoids are shown with 30% probability.)

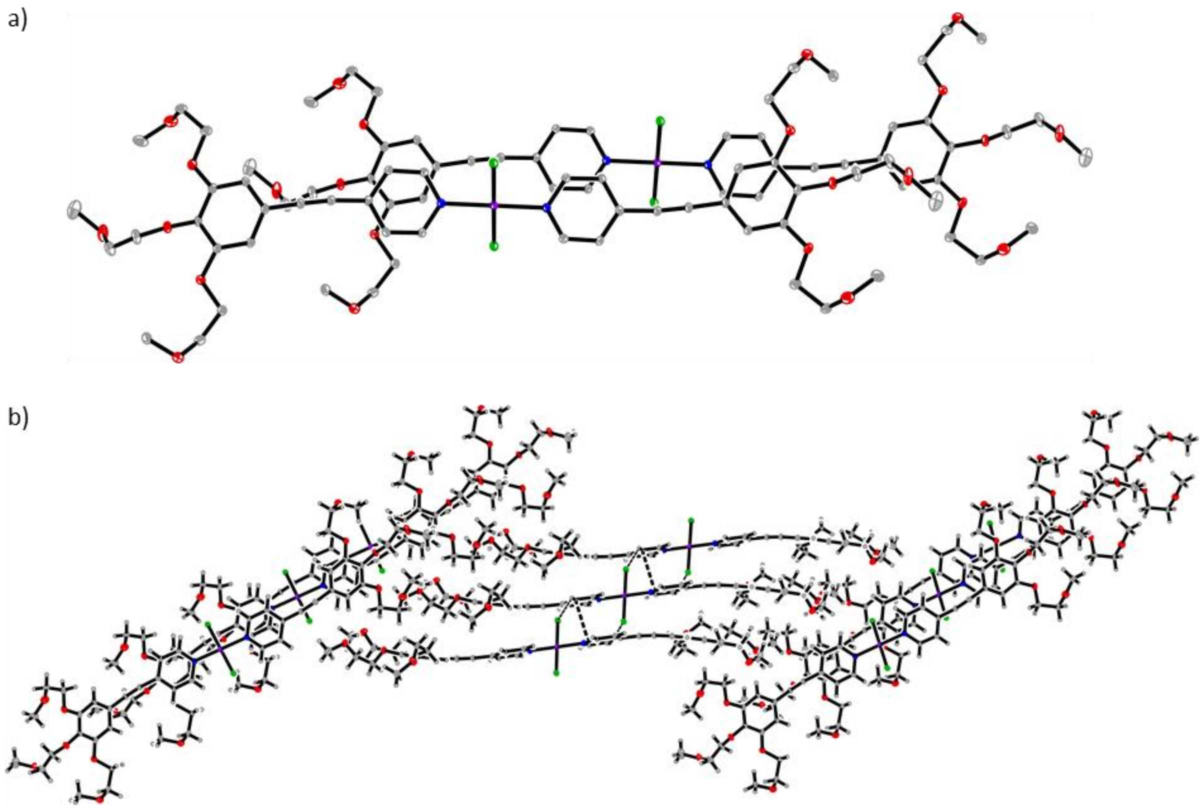

Figure S6. Excerpt of the packing diagram of compound **3** presenting a) top view of two neighboring molecules illustrating the positioning of the π-systems; b) the zigzag 2D network and the interaction mode between the ethylene glycol chains.

Table S3. Non-covalent intermolecular interactions in compound **3** (Å and deg).

D-H···*A*	*d*(*D*-H)	*d*(H···*A*)	*d*(*D*···*A*)	∠(*DHA*)
C3-H3···Cl1[#1]	0.95	2.86	3.622(4)	137.7
C23-H23B···C8[#2]	0.98	2.87	3.734(9)	147.8
C2-H2···O2[#3]	0.95	2.68	3.435(14)	136.3
C21-H21A···O3[#4]	0.99	2.43	3.018(9)	117.4
C22-H22A···*C*g2[#4]	0.99	2.41	3.187(8)	133.9
C15-H15A···O2[#5]	0.99	2.64	3.510(9)	146.0
*C*g1···*C*g1[#4]			3.405	
Pt1···Pt1[#6]			7.339	

Symmetry transformations used to generate equivalent atoms: [#1] -x+2, -y+1, -z; [#2] -x+7/2, y+1/2, -z+1/2; [#3] -x+3, -y+2, -z; [#4] -x+7/2, y-1/2, -z+1/2; [#5] -x+4, -y+2, -z; [#6] -1+x, y, z. *C*g1 is the centroid involving part of the pyridine ring containing C2, C3 and N1 atoms; *C*g2 is the centroid involving part of the phenyl ring containing C11, C12 and O1, O3 atoms.

C. Solid state NMR

Methods:

Solid-state ^{1}H MAS NMR spectra were collected on a BRUKER DSX 500 spectrometer operating at 11.7 T. A commercial Bruker 2.5 mm H/X/Y triple resonance MAS probe and 2.5 mm o.d. ZrO_2 rotors were used, employing MAS frequencies between 25.0 and 27.778 kHz. Radio-frequency nutation frequencies and ^{1}H chemical shifts were referenced on Adamantane (1.85 ppm)[13] and the magic angle was set using KBr[14] before each measurement. The $\pi/2$ pulse length for ^{1}H excitation was 2.5 µs. Double-quantum (DQ) recoupling was achieved using the Back-to-Back (BaBa) pulse sequence,[15] where exact rotor-synchronization was achieved via external triggering at the beginning of the excitation and reconversion blocks. Additionally, a z-filter of one rotor period was added. Excitation periods of one and four rotor periods were chosen. Relaxation delays were determined via the saturation-recovery method. For the ^{1}H MAS NMR spectra 4 scans were acquired, while the 2D ^{1}H-^{1}H double-quantum single-quantum (DQ-SQ) NMR correlation spectra were acquired using 32 scans for a total of 64 rotor-synchronized t_1-increments. Phase-sensitive 2D NMR datasets were obtained using the STATES-TPPI method.[16]

$^{13}C\{^{1}H\}$ CP/MAS and $^{13}C\{^{1}H\}$ Heteronuclear Correlation (HETCOR) NMR spectra of **1** were acquired on a BRUKER AVANCE III spectrometer operating at 7.04 T (^{1}H: 300 MHz, ^{13}C: 75.4 MHz). A commercial BRUKER 4 mm H/X double resonance WVT MAS probe and 4 mm o.d. ZrO_2 rotors were used, with MAS frequencies between 10.0 kHz and 12.5 kHz. ^{1}H and ^{13}C chemical shifts were referenced on adamantane (^{1}H: 1.85 ppm, ^{13}C: 38.5 ppm/29.5 ppm)[17]; radio-frequency nutation frequencies were calibrated directly on each sample and the magic angle was set using KBr[14] before each measurement. $^{13}C\{^{1}H\}$ CP/MAS and 2D $^{13}C\{^{1}H\}$ HETCOR NMR spectra of **2**, **3**, and **4** were acquired on a BRUKER AVANCE DSX spectrometer operating at 9.4 T (^{1}H: 400 MHz, ^{13}C: 100.4 MHz). A commercial BRUKER 4 mm H/X/Y triple resonance DVT MAS probe and 4 mm o.d. ZrO_2 rotors were used, with MAS frequencies between 10.0 kHz and 12.5 kHz. ^{1}H and ^{13}C chemical shifts were referenced on Adamantane (^{1}H: 1.85 ppm, ^{13}C: 38.5 ppm/29.5 ppm)[17]; radio-frequency (RF) nutation frequencies were calibrated directly on each sample and the magic angle was set using KBr[14] before each measurement.

^{1}H RF nutation frequencies of 62.5 kHz and ^{13}C RF nutation frequencies of ca. 50 kHz were used for the cross-polarization, 80 kHz was used during swfTPPM high-power ^{1}H decoupling[18]. CP/MAS experiments were performed using the ramped-amplitude CP

experiment with a 70-100% amplitude ramp on the ^{13}C channel, with the ^{13}C ramp nutation-frequencies optimized on each sample. CP-contact times were varied for different experiments and are indicated for each spectrum.

To further increase selectivity, several HETCOR spectra were acquired using the LG-CP technique. LG-CP differs from regular CP (or Hartmann-Hahn CP) in that the spin-lock pulse on the ^{1}H channel employs a frequency offset according to the Lee-Goldberg condition, thereby supressing the ^{1}H-^{1}H dipolar couplings and prohibiting polarization shuttling between ^{1}H nuclei. This results in a more selective signal transfer, albeit at the cost of overall signal intensity. FSLG-decoupling was used to enhance resolution of the indirect ^{1}H-dimension during HETCOR experiments with decoupling ^{1}H RF frequencies between 83.3 and 100 kHz, Phase-sensitive 2D NMR data were obtained using the STATES-TPPI method.[16]. A Gaussian apodization function (GB = 0.1, LB = -10 Hz) was used in the F2 dimension, and the QSINE (SSB = 5) window function was applied to the F1 dimension of all HETCOR spectra.

Supplementary solid-state NMR data:

2D ^{1}H-^{1}H DQ-SQ correlation NMR

In addition, two sets of cross-correlations are observed, one originating from two inequivalent protons at the pyridine moiety (δ_{DQ}= ~15 ppm), and one set corresponding to aromatic protons coupling to ethylene protons (δ_{DQ}= 10-12 ppm). The former correlations are almost identical in **1**, **2**, and **3**, while the latter differ in relative intensities throughout the sample set. Since the oligoethylene glycol groups are attached to the OPE backbone right next to proton position **c** (δ_{SQ}= ~7 ppm), cross-correlations between the ethylene protons and proton **c** are always expected to occur. However, due to the large *intra*-molecular distance between protons **a** and **b** to the oligoethylene groups, any cross-correlations between these protons must be caused by packing effects. Unfortunately, the small chemical shift difference between **b** and **c** in **1**, **2**, and **3** prohibits any further interpretation, as the two contributions cannot be disentangled. Furthermore, the cross-correlations are likely affected by strong multi-spin effects caused by the dense spin network of the ethylene glycol, precluding any meaningful interpretation of the varying intensities. In addition, the aromatic protons **a** and **b** are strongly coupled, resulting in dipolar truncation effects [8,25].

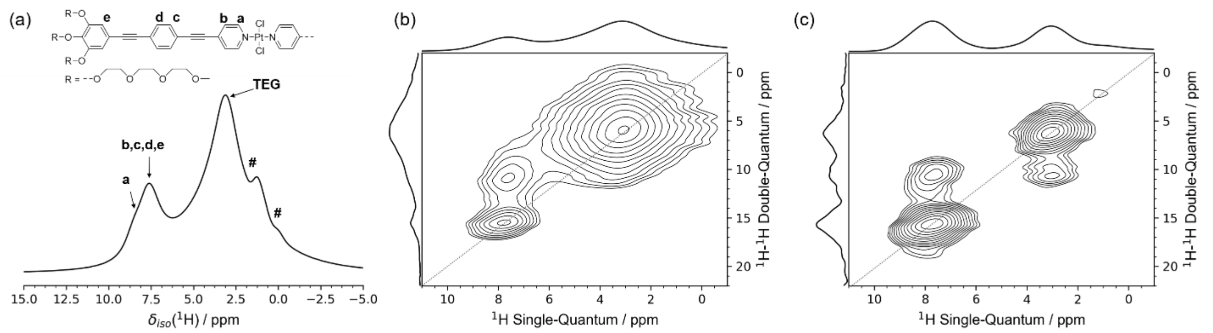

Figure S7. (a) ^{1}H MAS NMR of **5** with schematic showing the signal assignment on top. (b) 2D ^{1}H-^{1}H DQ-SQ NMR spectrum of **5** acquired using a single (b) and four (c) rotor periods of BaBa DQ recoupling. TEG designates the signal of the triethylene glycol side chains, and # denote signals of residual solvent molecules.

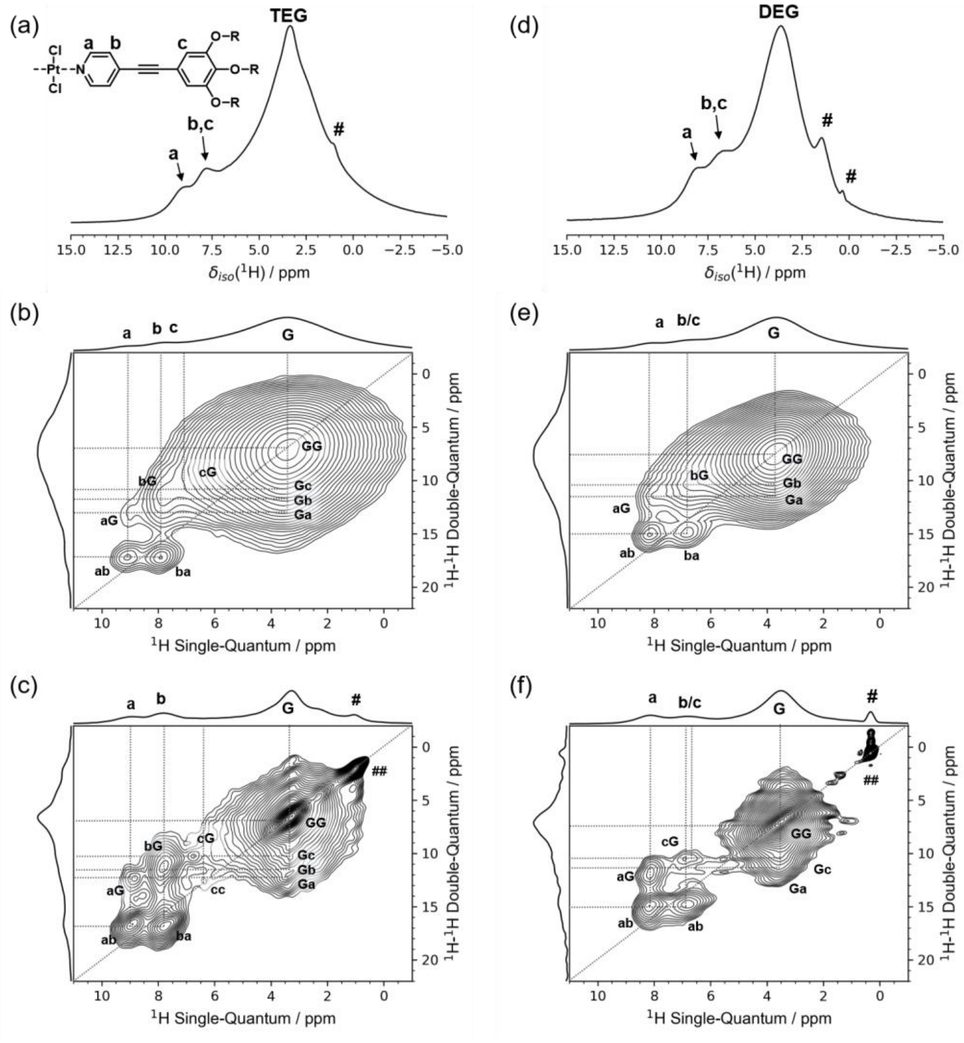

Figure S8. Left column shows the (a) ^{1}H MAS NMR of **1** with a schematic showing the signal assignment on top. TEG refers to the triethylene glycol sidechains, # denotes trapped residual solvent. Below are the ^{1}H-^{1}H DQ-SQ 2D NMR spectrum of **1** acquired using a single (b) and four (c) rotor periods of BaBa-DQ recoupling. The letter G denotes the ^{1}H signals associated with glycol CH_2. Right column shows the results for **2** using the same experiments; DEG denotes the ditheylene glycol protons.

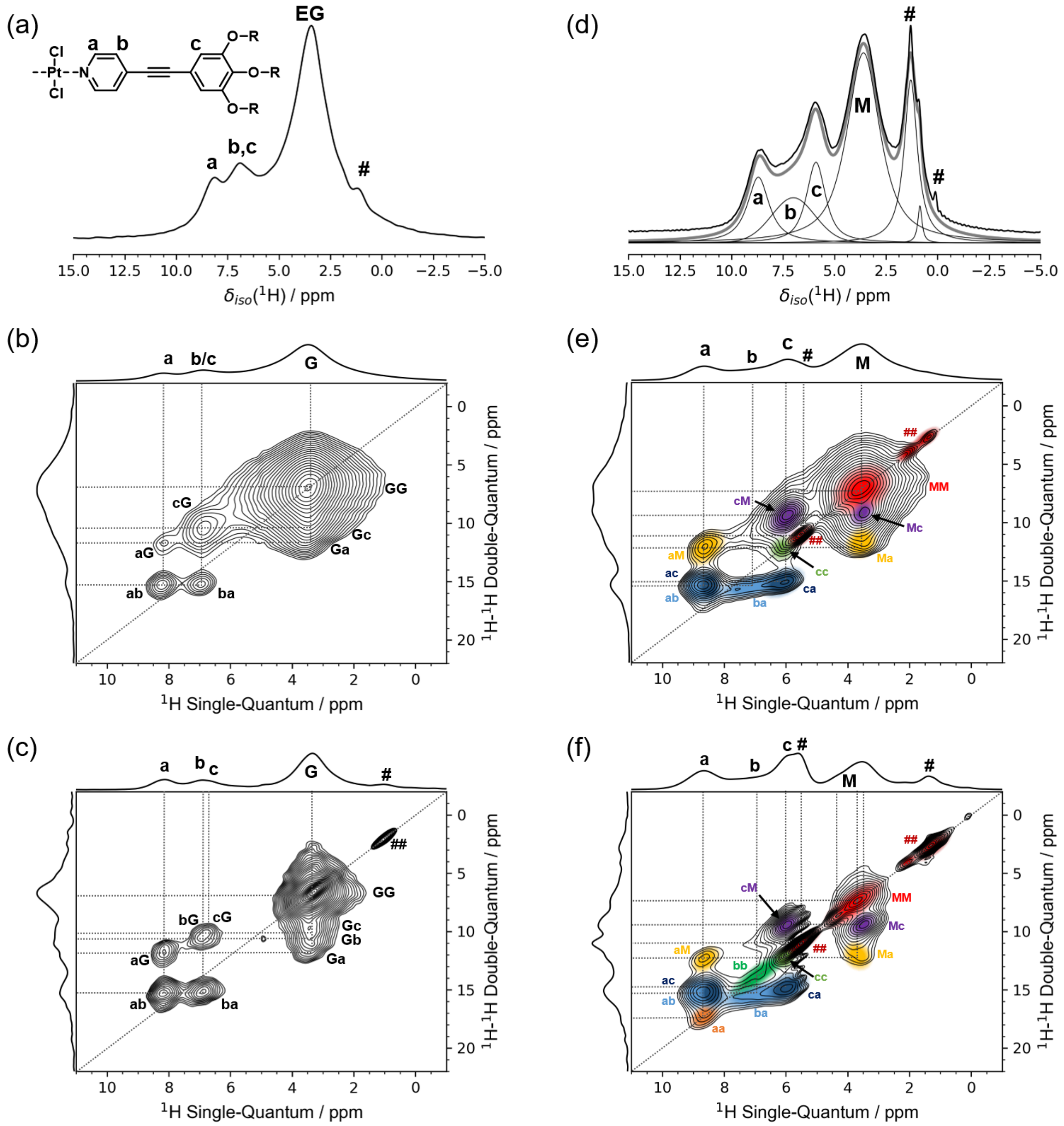

Figure S9. Left column shows the (a) ^{1}H MAS NMR of **3** with a schematic showing the signal assignment on top. EG refers to the ethylene glycol sidechains, # denotes trapped residual solvent. Below are the 2D ^{1}H-^{1}H DQ-SQ NMR spectrum of **3** acquired using a single (b) and four (c) rotor periods of BaBa DQ recoupling. The letter G denotes the ^{1}H signals associated with glycol CH_2. Right column shows the results for **4** of the same experiments, with M denoting the methoxy protons.

$^{13}C\{^{1}H\}$ CP/MAS NMR

$^{13}C\{^{1}H\}$ cross polarization (CP/MAS) and heteronuclear correlation (HETCOR):

The CP/MAS involves a polarization transfer via the direct ^{1}H-^{13}C dipolar coupling prior to acquiring the ^{13}C spectrum for signal enhancement. By varying the polarization transfer time ('contact time'), shorter and longer internuclear distances can be probed. This can be further used to correlate the ^{1}H chemical shift to the ^{13}C chemical shift, resulting in a two-dimensional experiment (HETCOR). Using LG-CP instead of regular CP leads to a slower build-up of signal, though the suppression of ^{1}H-^{1}H interactions during transfer yields a more selective transfer aiding in distinguishing between spatially close and distant C-H pairs.

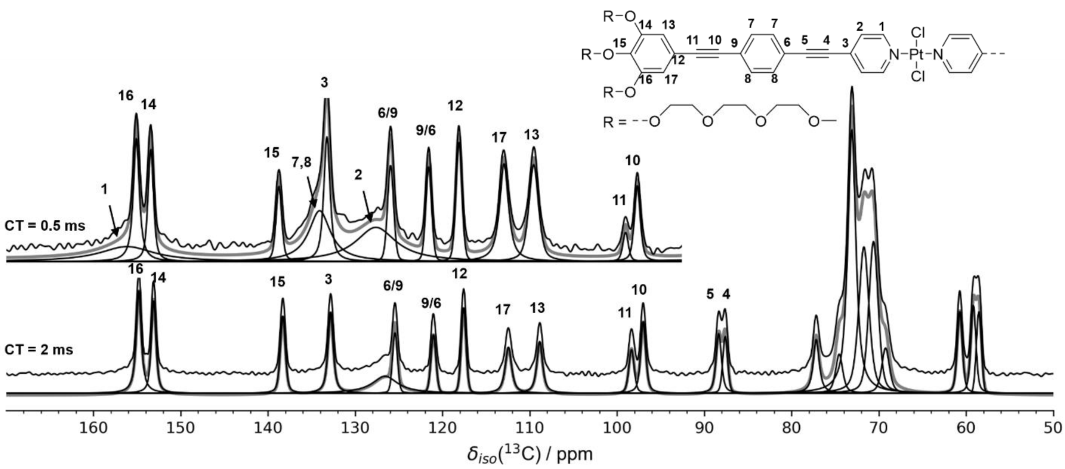

Figure S10. $^{13}C\{^{1}H\}$ CP/MAS NMR spectra of **5** acquired at 7.05 T and 12.5 kHz MAS. Contact times are indicated at the lower left corner. Also shown is the spectral deconvolution and peak assignment for the aromatic core of the ligand. Note that due to differences in packing, carbons 7 & 8, 14 & 16, and 13 & 17 are inequivalent in the solid state.

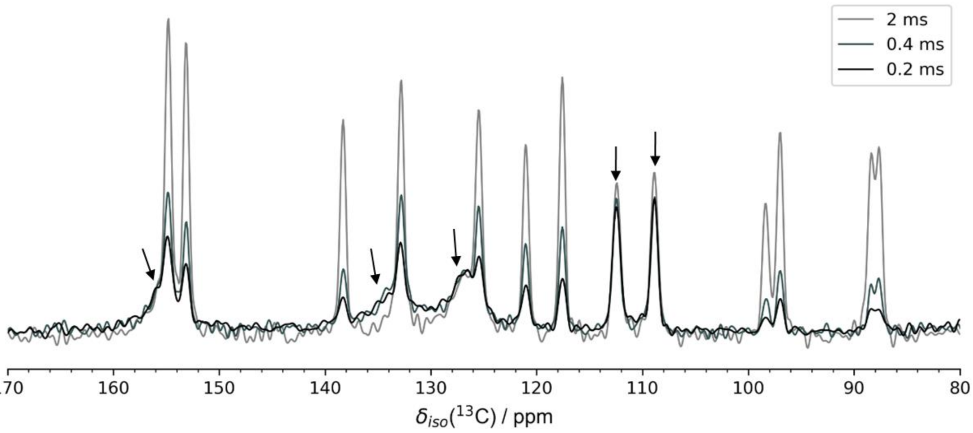

Figure S11. $^{13}C\{^{1}H\}$ CP/MAS NMR spectra of **5**. The CP contact time was varied between 0.2 ms (black line), 0.4 ms (dark gray), and 2 ms (light gray). Measured at 7.05 T, at 12.5 kHz MAS.

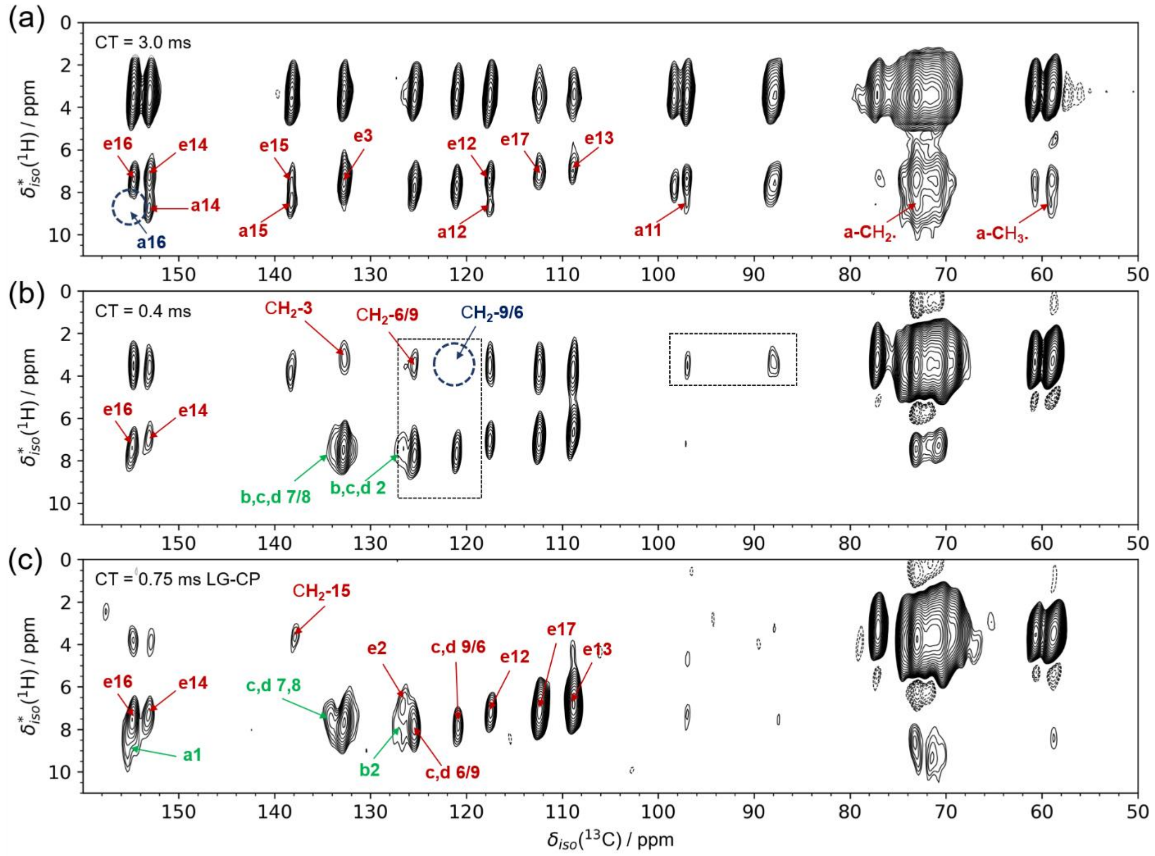

Figure S12. 2D ^{13}C{^{1}H} HETCOR NMR spectra of **5** using (a) 3 ms conventional CP, (b) 0.4 ms conventional CP, and (c) 0.75 ms LG-CP for the polarization transfer. Observed correlations are marked in red, expected but not observed correlations are marked in blue. The dashed boxes are discussed in the text. To maintain a sufficient S/N ratio under short CP contact times, the number of scans was increased from 32 (a) to 64 (b) and 128 (c) scans per increment. Red indicators mark well-resolved correlations, green indicators highlight broaden correlation peaks barely visible in this representation. Blue markers are discussed in the text.

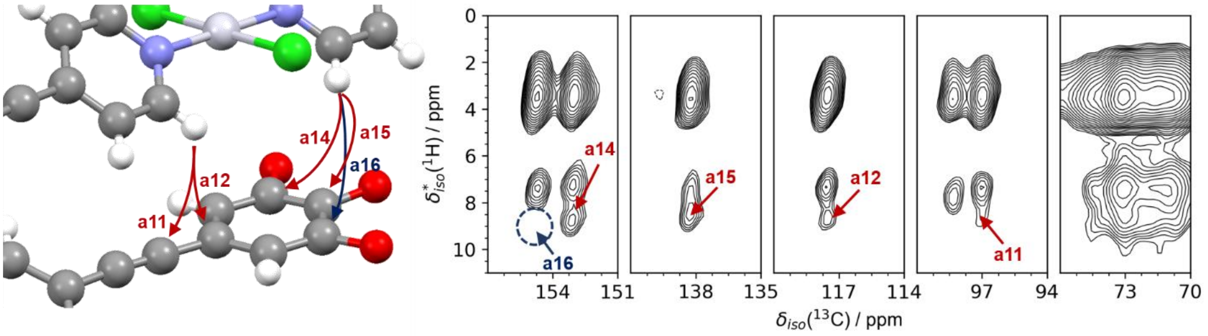

Figure S13. Left: a cutout of the crystal structure of **5**, showing the Pt-complexes center situated near the next complexes TEG groups. The associated ^{13}C{^{1}H} correlations from **Fehler! Verweisquelle konnte nicht gefunden werden.**a are shown in the right panels. Correlations between aromatic protons situated at the complexes center and TEG carbons are also present but are omitted from the cartoon for clarity.

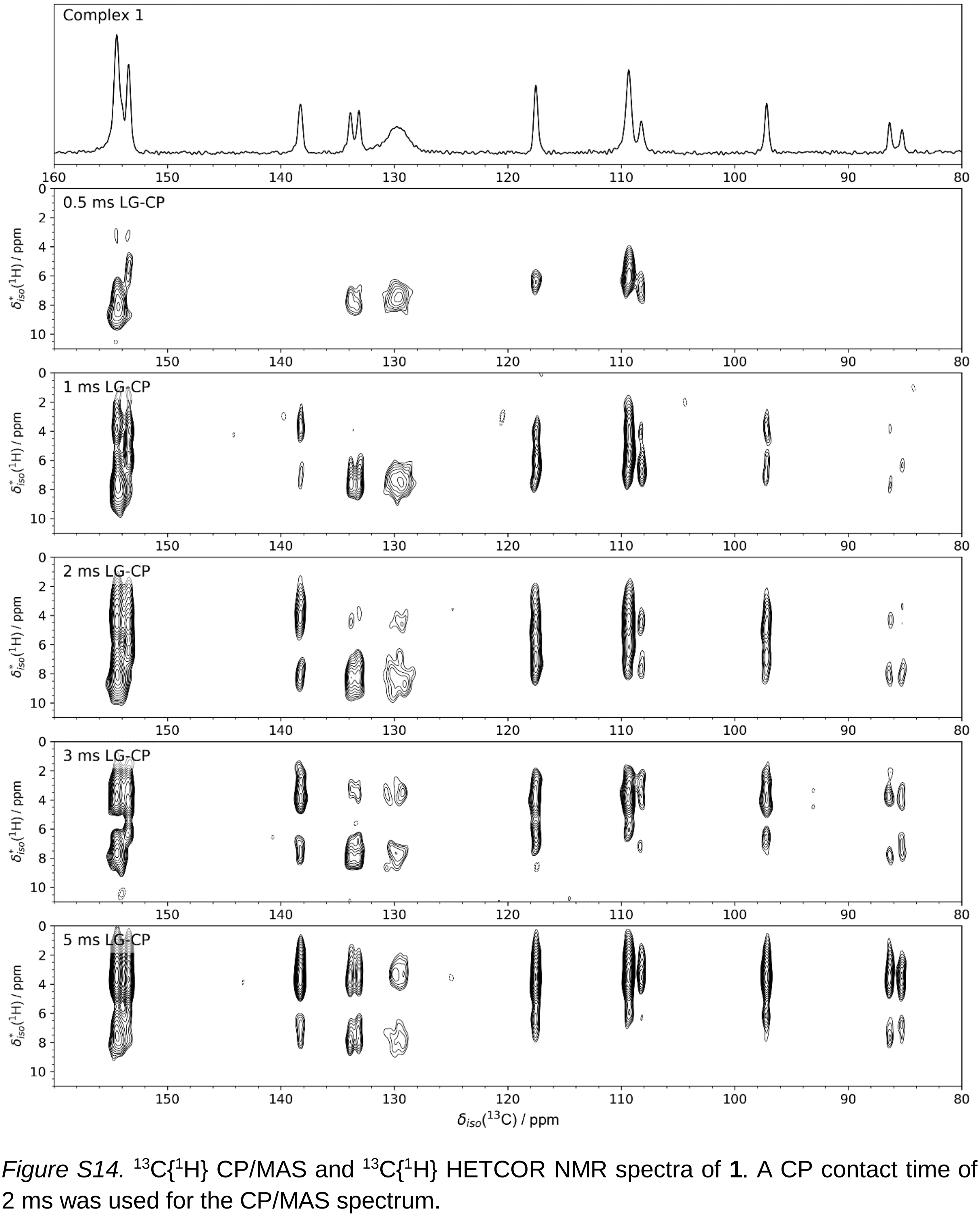

Figure S14. $^{13}C\{^1H\}$ CP/MAS and $^{13}C\{^1H\}$ HETCOR NMR spectra of **1**. A CP contact time of 2 ms was used for the CP/MAS spectrum.

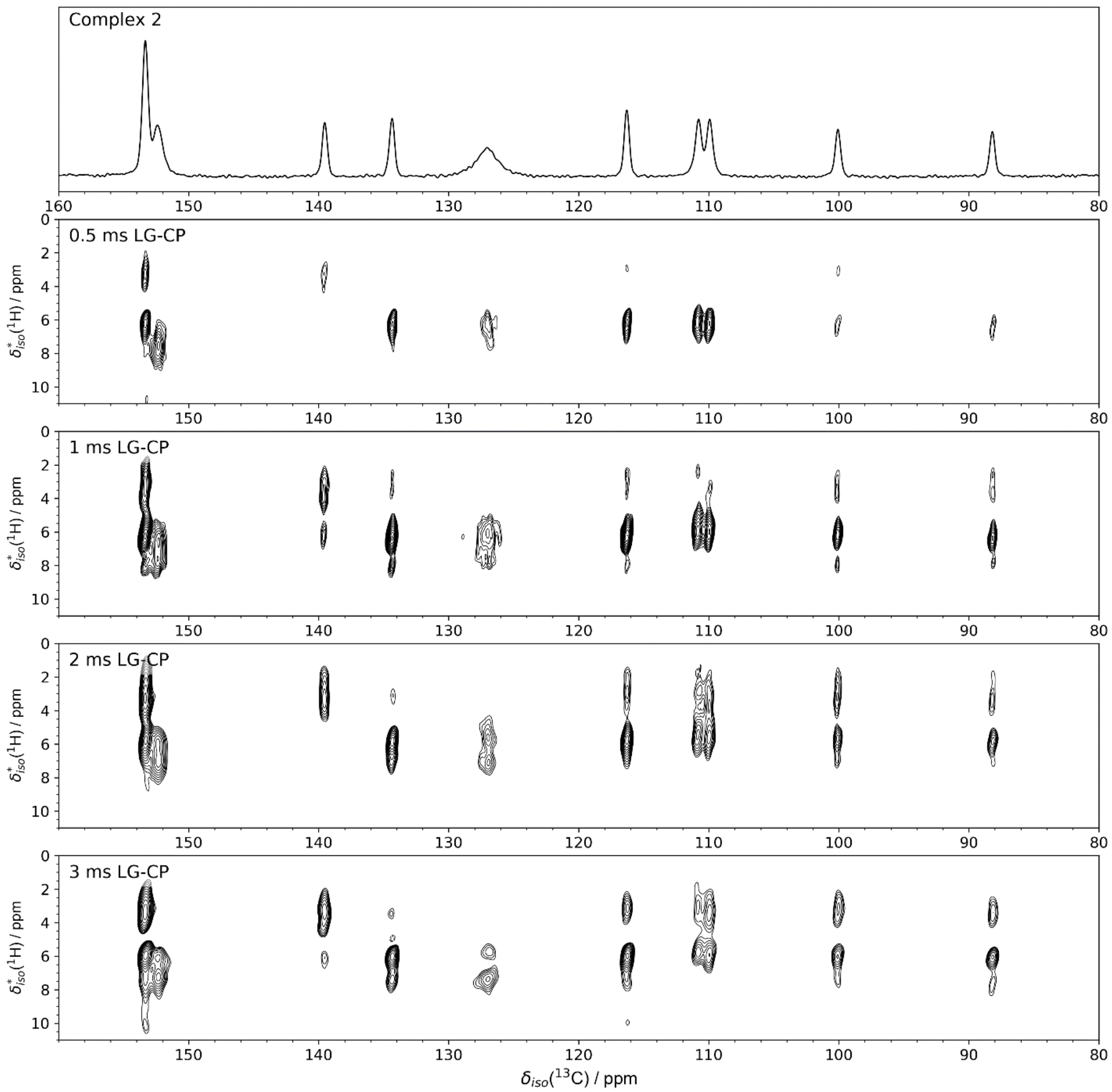

Figure S15. $^{13}C\{^1H\}$ CP/MAS and $^{13}C\{^1H\}$ HETCOR NMR spectra of **2**. A CP contact time of 2 ms was used for the CP/MAS spectrum.

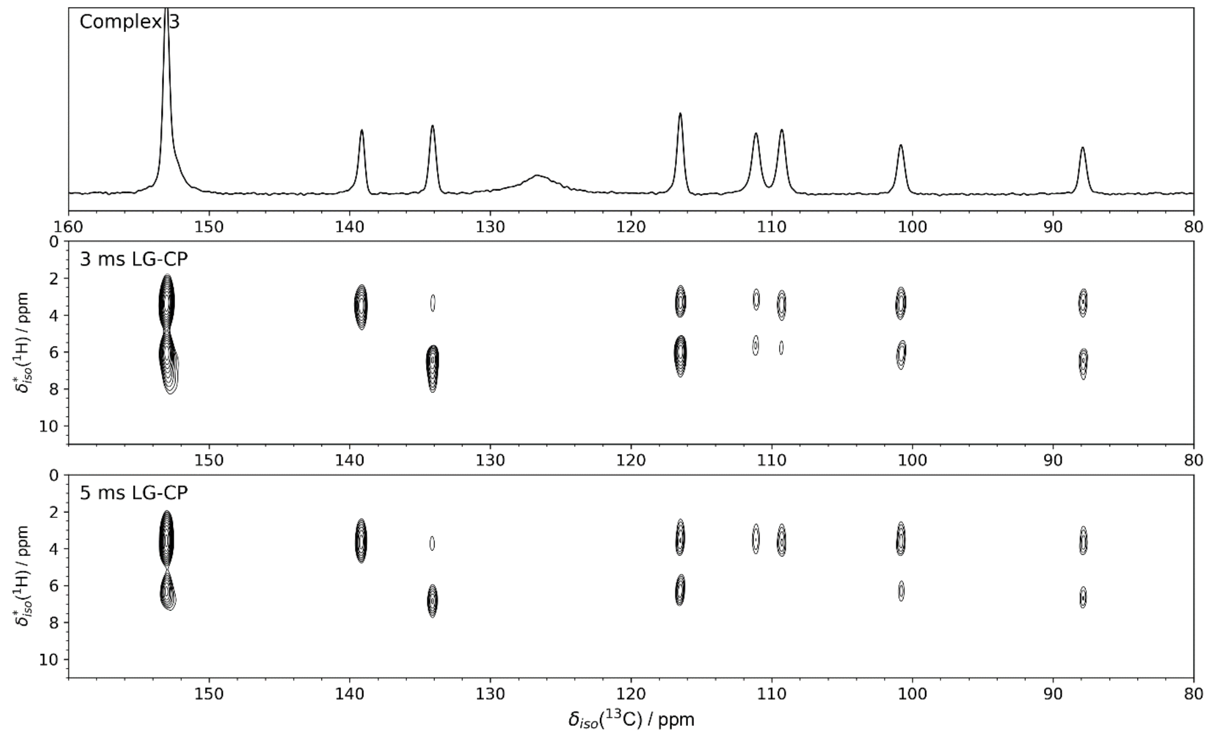

Figure S16. $^{13}C\{^1H\}$ CP/MAS and $^{13}C\{^1H\}$ FSLG-HETCOR spectra of **3**. A CP contact time of 2 ms was used for the CP/MAS spectrum.

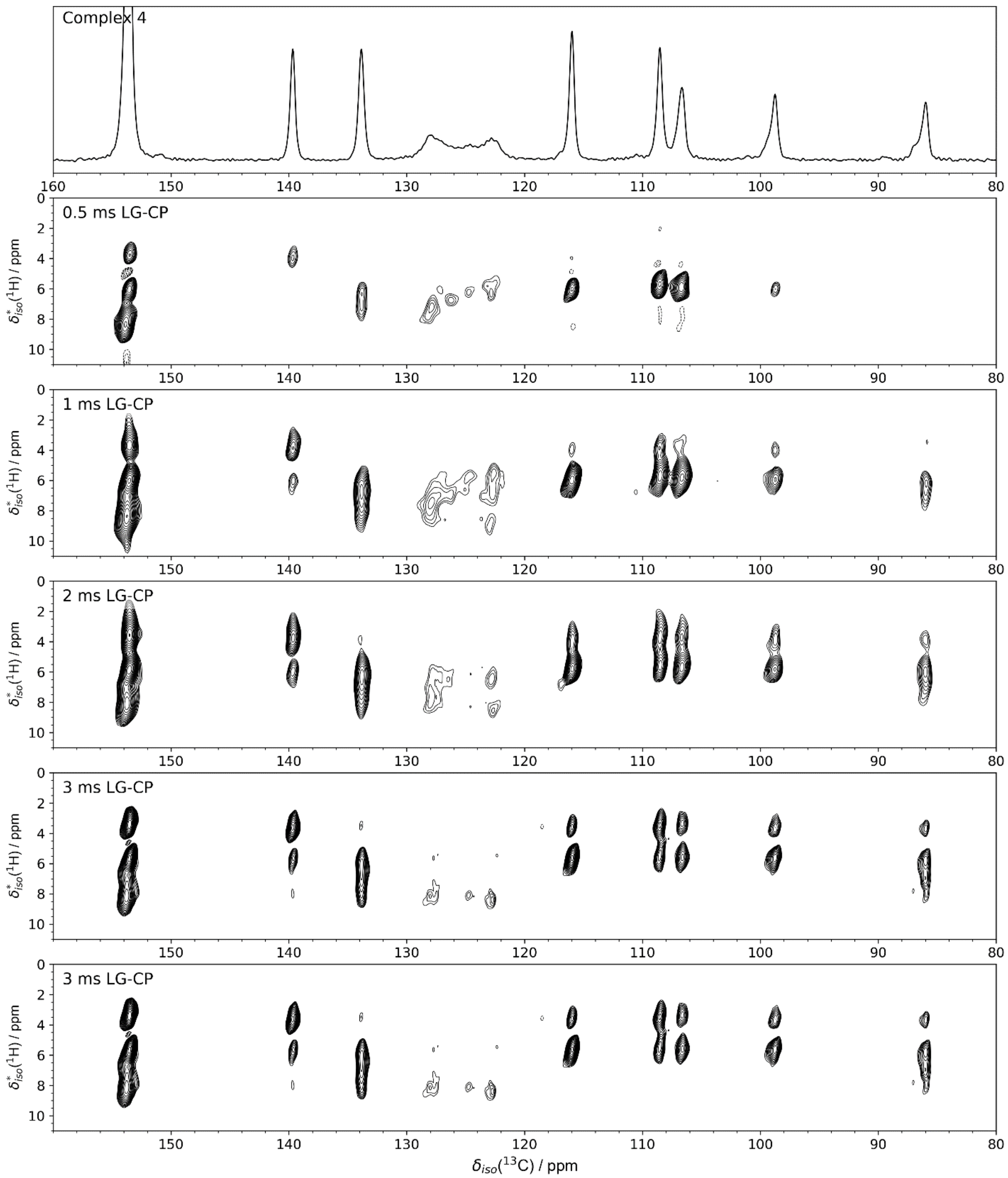

Figure S17. $^{13}C\{^1H\}$ CP/MAS and $^{13}C\{^1H\}$ FSLG-HETCOR spectra of **4**. A CP contact time of 2 ms was used for the CP/MAS spectrum.

D. XRD Analysis

Methods:

XRD diffraction analysis. Powder XRD diffraction experiments have been conducted on a ®Rigaku Smartlab X-ray diffractometer in parallel beam geometry with Cu-Kα radiation. The measurement steps were 0.01 deg with a measurement time per step of 0.6 s, using a voltage of 45 kV and a current of 190 mA. All samples used for XRD measurements were powdered samples and were obtained under identical conditions as those used for the preparation of the single crystals discussed in the main text (EtOAc/Et_2O solutions) on a glass microscope plate from Fisherbrand^{TM} with a thickness of 0.8-1 mm.

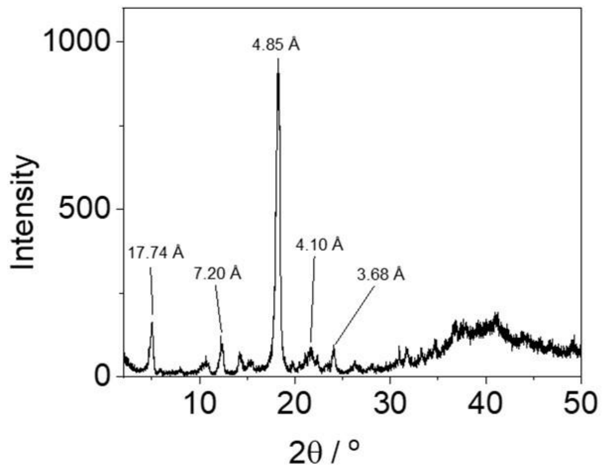

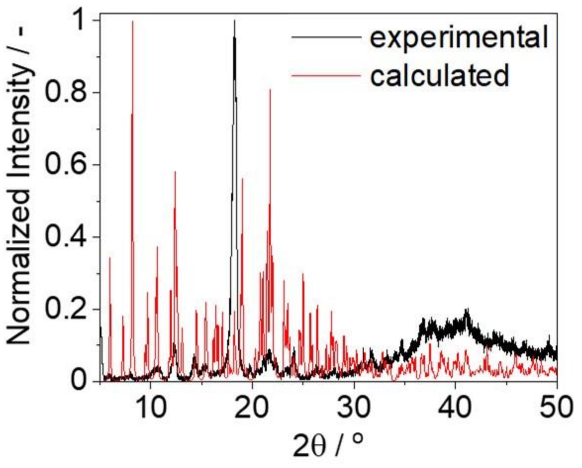

Figure S18. XRD pattern (λ = 1.5406 Å) of a thin film of **1** between 2 and 50° (left) and comparison with the predicted XRD pattern calculated based on the single crystal data (right).

XRD analysis of **1** shows only a relatively small number of pronounced reflexes, in particular when comparing to the diffraction patterns of **2**-**4**. This observation can easily be explained by the lower crystallinity of the sample as the sterically demanding TEG chains cannot order in a perfect 3D assembly, during the employed precipitation protocol. This hypothesis is further supported by comparison with the predicted XRD diffraction pattern based on the corresponding single crystal data, revealing only small experimental reflexes instead of defined ones. Nevertheless, a number of reflexes corresponding to characteristic intermolecular distances can be observed. In particular the reflex corresponding to a distance of 17.74 Å can be attributed to the intermolecular Pt-Pt distance, which was reported as 18.11 Å in the single crystal and matches well with the predicted XRD pattern. This minor discrepancy can be rationalized by precipitation effects. Additionally, a pronounced reflex at 4.85 Å can be observed. Based on the comparison with the single crystal analysis we refer this distance to the intermolecular distance between one Pt molecule and the methylene unit of a neighbouring TEG chain in the 1D stack (see also Fig. 1b), which corresponds to a distance of 4.89 Å in the single crystal. Comparison with the simulated XRD pattern suggests that the experimentally observed reflex may be a combination of multiple intermolecular close contacts. Thus, the conducted assignment should be interpreted with caution. Additionally, we tentatively assign the reflex at 2Θ = 24.04° corresponding to a distance of 3.68 Å to an additional intermolecular Pt-Me distance, which was observed in the single crystal as 3.80 Å. Interestingly, the additional Pt-Pt distances corresponding to the complex 3D network of 1D stacks observed in single crystal analysis (9.85, 12.71, 14,82, 15.70 and 16.44 Å) are absent from the XRD diffraction pattern. This observation further supports the assumption of a less crystalline sample obtained

for **1** in comparison to **2**-**4**, possibly induced by the sterically demanding TEG chains, which is again supported by the simulated XRD pattern, showing intense reflexes particularly for 2Θ < 15°, whereas the experimentally observed reflexes are considerably weaker. This region corresponds to long-range order, which is less effectively formed during the precipitation protocol.

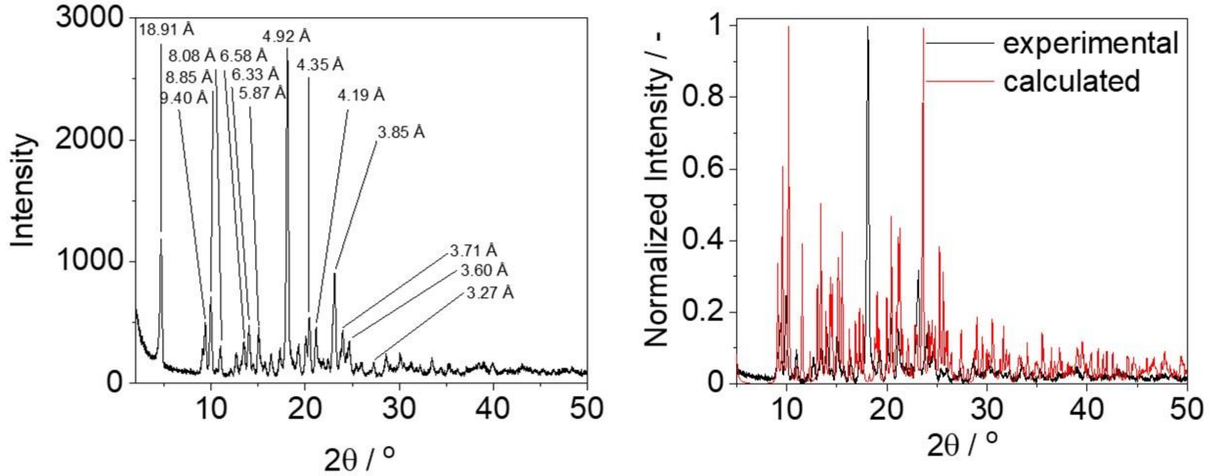

Figure S19. XRD pattern (λ = 1.5406 Å) of a thin film of **2** between 2 and 50° (left) and comparison with the predicted XRD pattern calculated based on the single crystal data (right).

XRD analysis of **2** discloses a significant increase in reflexes with a concomitant increase in the overall intensity of the observed reflexes. Based on the comparison between the results obtained from XRD and single-crystal analysis a number of similarities can be observed. However, it should also be noted that minor discrepancies can be appreciated, mainly with respect to the distances in the 1D stack as depicted in Fig. 1c (*vide infra*). This observation can be readily explained by the high internal order observed in the interdigitation of the DEG chains in **2** in the single crystal, which can hardly be achieved during a precipitation approach, again this hypothesis is supported by the simulated XRD pattern based on single crystal analysis. Yet, the 3D intermolecular distances appear to be influenced to a lesser extent as the DEG do not influence the packing in the other two dimensions quite as excessively. Based on the accordance with the single crystal analysis of **2**, we could readily attribute the reflex corresponding to distances of 18.91 and 9.40 Å to the interstrand Pt-Pt distances of 18.52 and 9.71 Å observed in the single crystal. Additionally, we attribute the reflex at 2Θ = 10.93° corresponding to a distance of 8.08 Å to the intermolecular Pt-Pt distance within the same stack. Further, we tentatively assign the reflex corresponding to a distance of 8.85 Å to the intermolecular Pt-C distance between the Pt of a 1D stack and the closest C atom of the neighbouring stack, which was found to be 8.84 Å in the single crystal. The close aromatic contacts observed in the single crystal as well as in the NMR experiments could be confirmed by XRD analysis as the reflexes corresponding to distances of 6.58 and 3.27 Å match the distance between pyridine moieties within a 1D stack in the single crystal, which were observed to be approximately 3.2 Å. A relatively sharp reflex at 4.92 Å can also only be attributed to the 1D stack as described in the main text, as the intense nature of this reflex in combination with the relatively small distance indicates the immediate environment of the Pt atoms. Based on the comparison with the single crystal analysis, we attribute this reflex to the distance between the Pt molecules and the aromatic C atoms of the neighbouring molecule, which were found to be around 4.56 Å in the single crystal. This discrepancy is further highlighted by comparison with the predicted XRD pattern as no intense reflex can be observed in this part of the pattern. Based on the minor discrepancy of this distance compared to most other intermolecular close contacts that are matching the single crystal analysis and the predicted XRD pattern rather

well, we infer that the molecules occupy a slight rotational displacement with respect to the single crystal orientation, due to imperfect intercalation of the DEG chains during the precipitation protocol. While this arrangement affects the immediate Pt environment, most intermolecular distances remain largely unaffected since the molecular stacks orient themselves identically with respect to one another as they do in the single crystal.

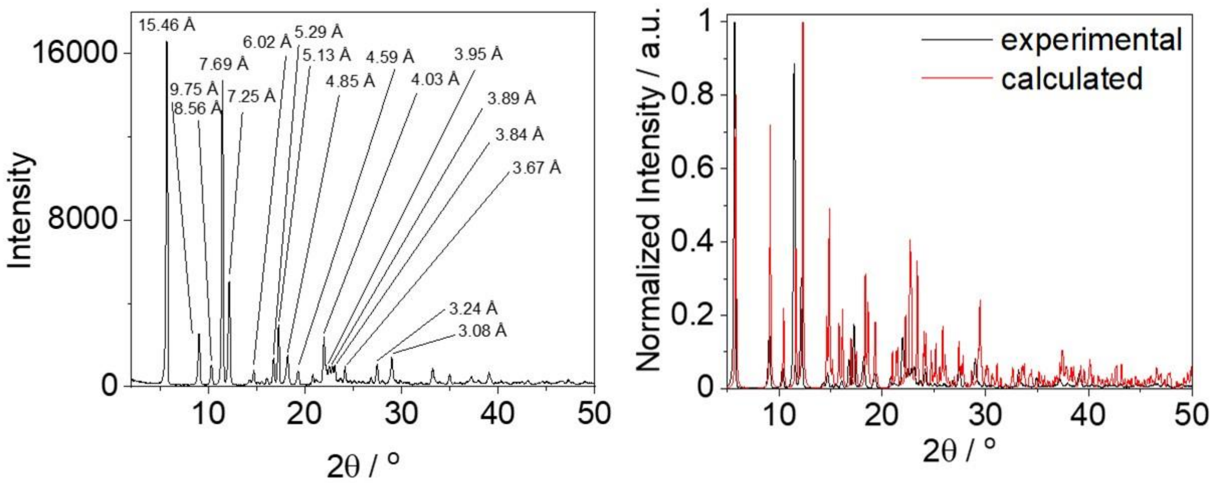

Figure S20. XRD pattern (λ = 1.5406 Å) of a thin film of **3** between 2 and 50° (left) and comparison with the predicted XRD pattern calculated based on the single crystal data (right).

XRD analysis of **3** reveals the highest overall intensity of all investigated samples, which is in line with the plethora of intermolecular interactions observed in single crystal analysis and confirmed by solid-state NMR. Further, the experimental results are in very good agreement with the predicted XRD results. In particular, we attribute the high crystallinity to the beneficial π-π stacking in combination with the relatively simple orientation of the shorter EG chains, which allows for a high degree of order even under precipitation conditions. We tentatively assign the reflexes corresponding to 15.46 and 7.69 Å to the intermolecular Pt-Pt distance within the 1D stack and the distance to the following Pt molecule respectively, as these reflexes are the most intense of the entire diffractogram and match well with the Pt-Pt distance observed for single crystal analysis (7.339 Å). Additionally, we attribute the reflex at 2Θ = 12.22°, corresponding to a distance of 7.25 Å, to the intermolecular Pt-Cl distance of the stack, while the distances of 9.75 and 8.56 Å are assigned to the intermolecular Cl-Cl distances of the 1D stack. Furthermore, the relatively short slipped arrangement of **3** allow for a plethora of close intermolecular contacts between 3.8 and 4 Å (4.03, 3.95, 3.89, 3.84 Å). These can be attributed to close contacts between the Pt atoms and the C atoms of the neighbouring molecule, which could also be observed in the single crystal (4.11, 4.06, 3.87, 3.86 Å). Ultimately, the close aromatic contacts could also be confirmed by the reflex corresponding to 3.24 Å, which matches the aromatic close contacts observed for **2** and supports the conclusions made based on solid-state NMR and single-crystal analysis.

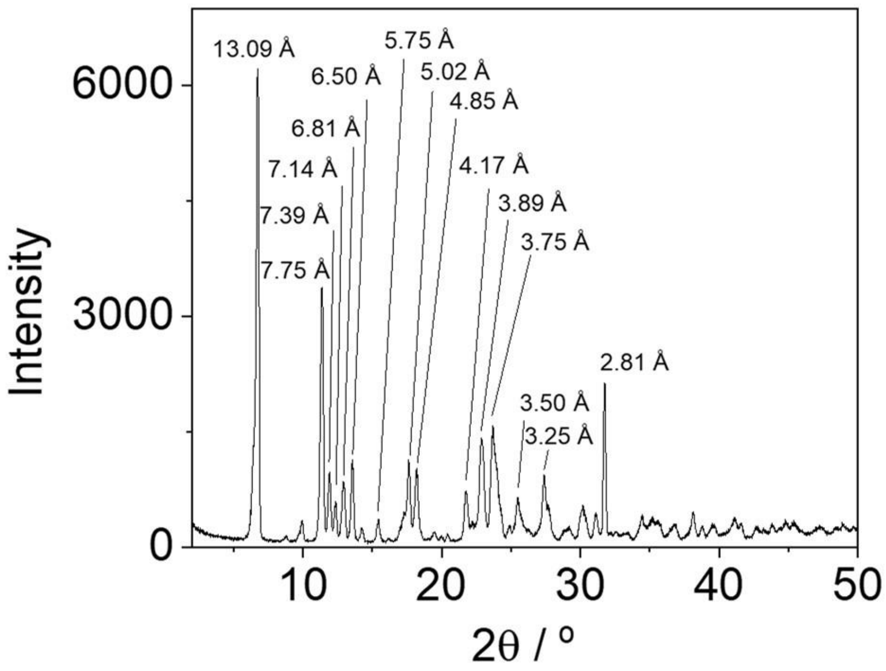

Figure S21. XRD pattern (λ = 1.5406 Å) of a thin film of **4** between 2 and 50°.

XRD analysis of **4** reveals a higher crystallinity compared to **1** but lower than that of **3**. This observation affirms the important role of the EG chain length in the self-assembly of the investigated series of complexes. Based on the results from solid-state NMR and the comparison with the structurally related compound published previously,[18] the packing has been assigned as a short-slipped, nearly parallel arrangement. Based on the intermolecular distances obtained from single-crystal analysis of **5**, we attribute the reflex at 7.75 Å to stem from Pt-Pt contacts between stacks (observed as 7.67 Å in the single crystal). Furthermore, the reflex at 6.50 and13.09 Å are also tentatively assigned to interstrand Pt-Pt distances. The reflex at 2*Θ* = 27.39°, corresponding to a distance of 3.25 Å, is assigned to the distance between the aromatic moieties, which matches the expected comparability with **2** and **3**. Notably, the intermolecular Pt-Pt distance within the 1D stack has been observed to be 4.47 Å in the single crystal of **5**, a reflex corresponding to this distance is absent from the diffractogram of **4**. However, reflexes corresponding to 4.85 and 5.02 Å are tentatively assigned to stem from the intermolecular Pt-Pt (4.85 Å) and Pt-Cl (5.02 Å) contacts, respectively. This small deviation (~0.4 Å) can be rationalized by the smaller aromatic surface of **4** compared to **5**, in combination with the torsion angle between the Cl-Pt-N and the Pt-N-C plane observed at the metal center. The increase in aromatic surface in **5** compared to **4** can overcome the steric demand of the bulky, out-of-plane Cl atoms better than the shorter aromatic surface in **4**. This can lead to a slightly distorted arrangement and a minor increase in Pt-Pt distance. Nevertheless, this minor displacement still results in an overall short slipped arrangement, which explains the absence of multiple intermolecular close contacts in solid-state NMR compared to samples of **2** and **3**. Additionally, it should be noted that multiple reflexes (see for example 2*Θ* = 27.75, 25.69, 24.18, 17.23°) exhibit a shoulder, indicating another intermolecular close contact, similar to that responsible for the main reflex. This observation could possibly suggest the existence of another packing similar to that proposed based on NMR, supporting the proposed possibility of polycrystallinity in the sample used for solid-state NMR analysis.

E. References

[1] T. Yasuda, T. Shimizu, F. Liu, G. Ungar, T. Kato, *J. Am. Chem. Soc.* **2011**, *133*, 13437–13444.

[2] a) M. Ouchi, Y. Inoue, Y. Liu, S. Nagamune, S. Nakamura, K. Wada, T. Hakushi, *Bull. Chem. Soc. Jpn.* **1990**, *63*, 1260–1262; b) R. Heathcote, J. A. S. Howell, N. Jennings, D. Cartlidge, L. Cobden, S. Coles, M. Hursthouse, *Dalton Trans.* **2007**, 1309–1315;

[3] W.-S. Li, Y. Yamamoto, T. Fukushima, A. Saeki, S. Seki, S. Tagawa, H. Masunaga, S. Sasaki, M. Takata, T. Aida, *J. Am. Chem. Soc.* **2008**, *130*, 8886–8887.

[4] S. Chen, X. Huang, S. Decurtins, C. Albrecht, S.-X. Liu, *Polyhedron* **2017**, *134*, 287–294.

[5] D. Faye, H. Zhang, J.-P. Lefevre, J. Bell, J. A. Delaire, I. Leray, *Photochem. Photobiol. Sci.* **2012**, *11*, 1737–1743.

[6] *APEX3;* Bruker AXS Inc, Madison, Wisconsin, USA, **2016**.

[7] *SAINT;* Bruker AXS Inc, Madison, Wisconsin, USA, **2015**.

[8] *SADABS;* Bruker AXS Inc, Madison, Wisconsin, USA, **2015**.

[9] G. M. Sheldrick, *Acta Cryst. Sect. A* **2015**, *71*, 3–8.

[10] G. M. Sheldrick, *Acta Cryst. Sect. C* **2015**, *71*, 3–8.

[11] R. W. W. Hooft, Nonius B. V, *COLLECT*: Program for Collecting Data on CCD Area Detectors, Delft, Niederlande, **1998**.

[12] Z. Otwinowski, W. Minor, *Methods Enzymol.* **1997**, *276*, 307–326.

[13] Z. Otwinowski, D. Borek, W. Majewski, W. Minor, *Acta Cryst. Sect. A* **2003**, *59*, 228–234.

[14] *XP – Interactive molecular graphics;* Bruker AXS Inc, Madison, Wisconsin, USA, **1998**.

[15] J. C. C. Chan, R. Tycko, *J. Chem. Phys.* **2003**, *118*, 8378–8389.

[16] L. A. O’Dell, R. W. Schurko, *Chem. Phys. Lett.* **2008**, *464*, 97–102.

[17] K. J. Harris, A. Lupulescu, B. E. G. Lucier, L. Frydman, R. W. Schurko, *J. Magn. Reson.* **2012**, *224*, 38–47.

[18] N. K. Allampally, M. J. Mayoral, S. Chansai, M. C. Lagunas, C. Hardacre, V. Stepanenko, R. Q. Albuquerque, G. Fernández, *Chem. Eur. J.* **2016**, *22*, 7810–7816.
